# Metal‐only Lewis Pairs of Rhodium with *s*, *p* and *d*‐Block Metals

**DOI:** 10.1002/chem.202003167

**Published:** 2020-11-09

**Authors:** Sonia Bajo, Macarena G. Alférez, María M. Alcaide, Joaquín López‐Serrano, Jesús Campos

**Affiliations:** ^1^ Instituto de Investigaciones Químicas (IIQ) Departamento de Química Inorgánica and Centro de Innovación en Química Avanzada (ORFEO-CINQA) Consejo Superior de Investigaciones Científicas (CSIC) University of Sevilla Avenida Américo Vespucio 49 41092 Sevilla Spain

**Keywords:** bimetallic compounds, dative bond, metal–metal bond, metal-only Lewis pair, rhodium NMR

## Abstract

Metal‐only Lewis pairs (MOLPs) in which the two metal fragments are solely connected by a dative M→M bond represent privileged architectures to acquire fundamental understanding of bimetallic bonding. This has important implications in many catalytic processes or supramolecular systems that rely on synergistic effects between two metals. However, a systematic experimental/computational approach on a well‐defined class of compounds is lacking. Here we report a family of MOLPs constructed around the Rh^I^ precursor [(*η* ^5^‐C_5_Me_5_)Rh(PMe_3_)_2_] (**1**) with a series of *s*, *p* and *d*‐block metals, mostly from the main group elements, and investigate their bonding by computational means. Among the new MOLPs, we have structurally characterized those formed by dative bonding between **1** and MgMeBr, AlMe_3_, GeCl_2_, SnCl_2_, ZnMe_2_ and Zn(C_6_F_5_)_2,_ as well as spectroscopically identified the ones resulting from coordination to MBAr_F_ (M=Na, Li; BAr_F_^−^=[B(C_6_H_2_‐3,5‐(CF_3_)_2_)_4_]^−^) and CuCl. Some of these compounds represent unique examples of bimetallic structures, such as the first unambiguous cases of Rh→Mg dative bonding or base‐free rhodium bound germylene and stannylene species. Multinuclear NMR spectroscopy, including ^103^Rh NMR, is used to probe the formation of Rh→M bonds. A comprehensive theoretical analysis of those provides clear trends. As anticipated, greater bond covalency is found for the more electronegative acids, whereas ionic character dominates for the least electronegative nuclei, though some degree of electron sharing is identified in all cases.

## Graphical abstract

**A new family is born**: We report a family of metal‐only Lewis pairs based on the basic Rh^I^ compound [(*η* ^5^‐C_5_Me_5_)Rh(PMe_3_)_2_] with a series of *s*, *p* and *d*‐block metal fragments. We provide the structural characterization of those based on Ge, Sn, Al and Mg, as well as two Rh^I^→Zn^II^ adducts. Our combined experimental/computational approach provides valuable insights into the Rh→M dative bonding.

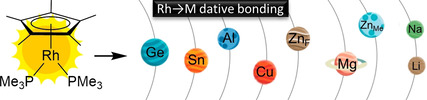

## Introduction

The unambiguous recognition of M−M bonding within the determination of the Mn_2_(CO)_10_ structure^[1]^ was a landmark discovery in transition metal chemistry and set the grounds for exciting developments in the field of polynuclear molecular compounds.^[2]^ Only a few years later, the existence of multiple bonding between metals was demonstrated by Cotton and co‐workers in [Re_2_Cl_8_],[^2^, ^3^] shattering at the same time the common belief of a maximum bond order of three, as seen in the *p*‐block. The area of metal‐to‐metal bonded compounds has discontinuously evolved since then, in a path teemed with milestones that include, to cite some paradigmatic examples, the first quintuple‐bonded dimetallic structure [Cr_2_{C_6_H_3_‐2,6‐Dip_2_}_2_]^[4]^ (Dip=C_6_H_3_‐2,6‐*i*Pr_2_) or the M^I^ dimers [Zn_2_Cp*_2_]^[5]^ (Cp*=[*η* ^5^‐C_5_Me_5_]^−^) and [Mg_2_(^Dip^Nacnac)_2_]^[6]^ (^Dip^Nacnac=[(DipNCMe_2_)_2_CH]^−^) with a M^I^−M^I^ bond.

A fascinating class of metal–metal bonded complexes that is receiving growing attention are those with M→M dative bonds, also referred as metal‐only Lewis pairs (MOLPs).^[7]^ Although noticed earlier,^[8]^ the first authoritative report on such a species dates back to 1967, when Nowell and Russell elucidated the solid‐state structure of [(*η* ^5^‐C_5_H_5_)(CO)_2_Co→HgCl_2_].^[9]^ Numerous studies based on a wide variety of transition metals were later disclosed, particularly during the last decade.^[10]^ Apart from the fundamental appeal of these species, the interest on their study is at the heart of transition metal reactivity. The basicity of a transition metal site is important for small molecule coordination (e.g. borane binding in borylation processes),^[11]^ as well as during oxidative addition reactions. In turn, the latter are elementary steps present in most catalytic cycles, as noticed from early reports.^[12]^ Thus, a better understanding of transition metal basicity (i.e. through the examination of metal‐only Lewis pairs)^[13]^ may provide important information to be assimilated by bond activation and catalysis research.

In addition, bimetallic dative bonding has implications in many catalytic processes that involve the participation of two metal fragments of contrasting electronic nature. For instance, a series of studies on Pd‐catalyzed Negishi and Sonogashira cross‐coupling reactions revealed the impact on catalytic performance of bimetallic Lewis acid‐base interactions between an electron rich Pd^II^ center and acidic Zn^II^ or Cu^I^ fragments.^[14]^ Unsupported MOLP compounds have also proved competent in the activation of a variety of E−H bonds (E=H, X, N, O) in which their individual monometallic constituents revealed themselves inactive.^[15]^ The incorporation of acidic metals or metalloids as σ‐acceptors Z‐type ligands in MOLP‐type structures permits structural and electronic modulation of the basic metal site,^[16]^ whereas the strength of the M→M dative bonding in thermally induced^[17]^ metal‐only frustrated Lewis pairs deeply impacts the reactivity and catalytic performance of the latter systems.^[18]^ In addition, metal‐to‐metal dative bonding has important implications in supramolecular and molecular engineering,^[19]^ as well as in host–guest chemistry.^[20]^

With all this in mind, it becomes obvious that a deep understanding of the nature of metal‐to‐metal bond in these molecular compounds and supramolecular aggregations will have an important impact in a range of areas. In fact, this has been a matter of intense debate, which is not surprising considering the set of bonding components that may be involved (i.e. ionic, covalent, dative, dispersion…). As such, unsupported systems in which the bond between the two metals is the sole force holding the two fragments together constitute ideal motifs to study, since other factors that may obscure bonding analysis are excluded. In their original report, Nowell and Russell postulated that [(*η* ^5^‐C_5_H_5_)(CO)_2_Co→HgCl_2_] could be considered a metallic Lewis acid–base adduct,^[9]^ as lately proposed for many other systems,[^18a^, ^21^] including those based on *d* ^8^‐*d* ^10^ interactions (referred to the last filled subshell of the bonding metals).^[22]^ An alternative description proposed by Pyykkö implies dispersion forces as the main component of the bimetallic bonding.^[23]^ However, more recent computational work speaks in favor of the former assumption, revealing that dispersion forces contribute to a lesser extent in these type of systems compared to the role of electrostatic and orbital interactions.^[24]^

**Figure 1:**
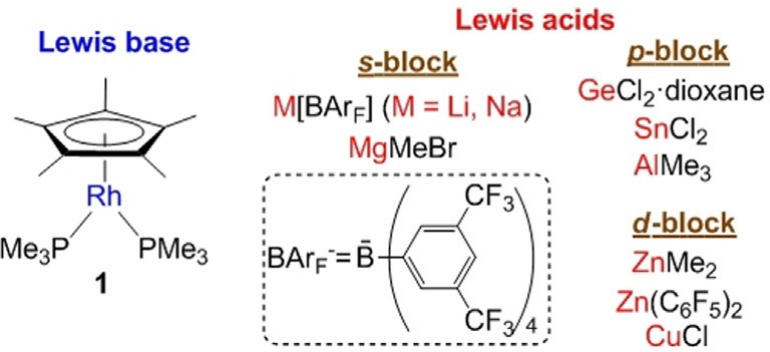
Metal Lewis basic (blue) and acidic (red) fragments employed in this work to access metal‐only Lewis pairs (MOLPs).

Most studies have either focused on the synthesis and structural characterization of a group of several MOLPs or on the computational analysis of previously reported bimetallic architectures of this kind. However, a more comprehensive and combined experimental/computational approach on a family of unsupported MOLPs is lacking. With this aim, we have selected the electron rich Rh^I^ compound [(*η* ^5^‐C_5_Me_5_)Rh(PMe_3_)_2_]^[25]^ (**1**) as a Lewis base to investigate a variety of MOLPs generated by its combination with well‐known metallic and metalloid Lewis acids (Figure 1). We provide not only the spectroscopic (including ^103^Rh NMR) and structural characterization of these uncommon compounds, but also a computational analysis of their Rh^I^→M bonding.

## Results and Discussion

The precise choice of **1** as the Lewis base to design MOLPs was made on the basis of several features: (i) the basic behavior of **1** has already been well established;^[25]^ (ii) PMe_3_ ligands enhance the nucleophilicity^[13a]^ of the Rh^I^ site compared to its more widely explored carbonyl analogue [(*η* ^5^‐C_5_Me_5_)Rh(CO)_2_];^[26]^ (iii) the robustness of (*η* ^5^‐C_5_Me_5_) ligand prevents undesired reactivity recorded for its unsubstituted (*η* ^5^‐C_5_H_5_) analogue;^[27]^ (iv) as a neutral Lewis base, its combination with neutral acids will minimize the ionic and electrostatic components of the Rh^I^→M bond; (v) as a pentacoordinated 18‐electron species, insertion reactions into polar bonds of the Lewis acid, or the formation of intermediate alkyl or hydride bridging species^[28]^ that would cloud analysis of the Rh^I^→M bond, will be less favored; and (vi) ^103^Rh is NMR active (*I*=1/2, 100 % abundant). With all this in mind, we have combined **1** with a variety of main group metal precursors as Lewis acids. With the exception of CuCl, we avoided the extensive use of transition metal electrophiles to circumvent more complex bonding pictures on grounds of their available *d* orbitals.

### Synthesis of Rh^I^ MOLPs with *s*‐Block Acids

The number of compounds exhibiting metalophilic interactions between transition and alkali metals is abundant.^[29]^ Systems that show identical or even reduced M−M bond lengths compared to the sum of their corresponding covalent atomic radii^[30]^ presumably present some degree of bond covalency. Although this is relatively common in the case of lithium,^[31]^ examples of its heavier congener sodium are less profuse.^[32]^ Considering rhodium, the weak interaction of square planar [RhCl_4_]^3−^ with a naked Na^+^ cation has been analyzed by computational means as the result of orbital overlapping.^[22a]^ The solid‐state structure of [Na(thf)_3_][Rh(*η* ^4^‐cod)Rh(P_3_Mes_3_)] reveals a short Rh−Na bond length of 3.105(2) Å,^[33]^ only slightly elongated with respect to the sum of their covalent radii (3.08 Å).^[30]^ As anticipated, support for covalent bonding was inferred from theoretical studies. It is important to remark that this type of Lewis acid–base interaction with alkali metals may promote interconversion between structural conformations in transition metal complexes,^[34]^ in turn a powerful tool for designing molecular machines.^[35]^

**Scheme 1:**
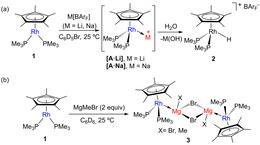
Synthesis of metal‐only Lewis pairs by combination of **1** and *s*‐block metal precursors (a) M[BAr_F_] (M=Li, Na) and (b) MgMeBr. Rapid formation of hydride **2** in wet solvents in all MOLPs reported herein was ascertained by the appearance of a low‐frequency ^1^H NMR signal recorded at −13.35 ppm (^2^ *J*_HP_=23, ^1^ *J*_HRh_=35 Hz).

We decided to explore the possibility of accessing unsupported MOLPs containing lithium and sodium cations. To prevent artificial elongation of the Rh→M bond due to steric repulsion,^[32b]^ we focused on lithium and sodium salts of the low‐coordinating tetrakis(3,5‐bis(trifluoromethyl)phenyl)borate anion (BAr_F_^−^), while using non‐coordinating solvents. Addition of either one equivalent of NaBAr_f_ or LiBAr_f_ to bromobenzene solutions of **1** result in immediate color darkening. Complete consumption of **1** is evinced by a pronounced decrease in the ^1^ *J*_PRh_ coupling constant of around 80 Hz (**[A⋅Li]**: 130 Hz; **[A⋅Na]**: 138 Hz; *c.f*. **1**: ^1^ *J*_PRh_=216 Hz), a distinctive feature that applies to all MOLPs prepared herein (see Table 2 below). Another common observation is the shift towards slightly lower frequencies of the ^1^H NMR signal associated to the pentamethylcyclopentadienyl ring, which resonates at 2.16 ppm for compound **1** (*c.f*. **[A⋅Li]** : *δ*=1.61 ppm; **[A⋅Na]**: *δ*=1.67 ppm). We hypothesize compounds **[A⋅Li]** and **[A⋅Na]** to be the targeted alkali MOLPs (Scheme 1 a), whose existence is further supported by computational means (vide infra), though weak *η* ^5^‐coordination to the empty face of the Cp* ligand cannot be ruled out. At this stage, we defer a definitive proposal due to the lack of structural data. All our attempts to grow single crystals of these species were unsuccessful. We recovered in all cases either crystalline M[BAr_F_] (M=Li, Na), which may illustrate the weakness of the Rh→Li/Na interaction, or observed the formation of the corresponding Rh^III^ hydride [(*η* ^5^‐C_5_Me_5_)Rh(PMe_3_)_2_H][BAr_F_]^[24]^ (**2**), the latter formed due to the presence of adventitious water. Analogous cooperative reactivity has been reported for other MOLPs based on [Pt(P*t*Bu_3_)_2_]. [^14a^, ^14b^] For further validation, compound **2** could be independently synthesized by addition of equimolar amounts of ammonium salts to **1** and it has been utilized as a benchmark species to investigate the bonding.

**Figure 2:**
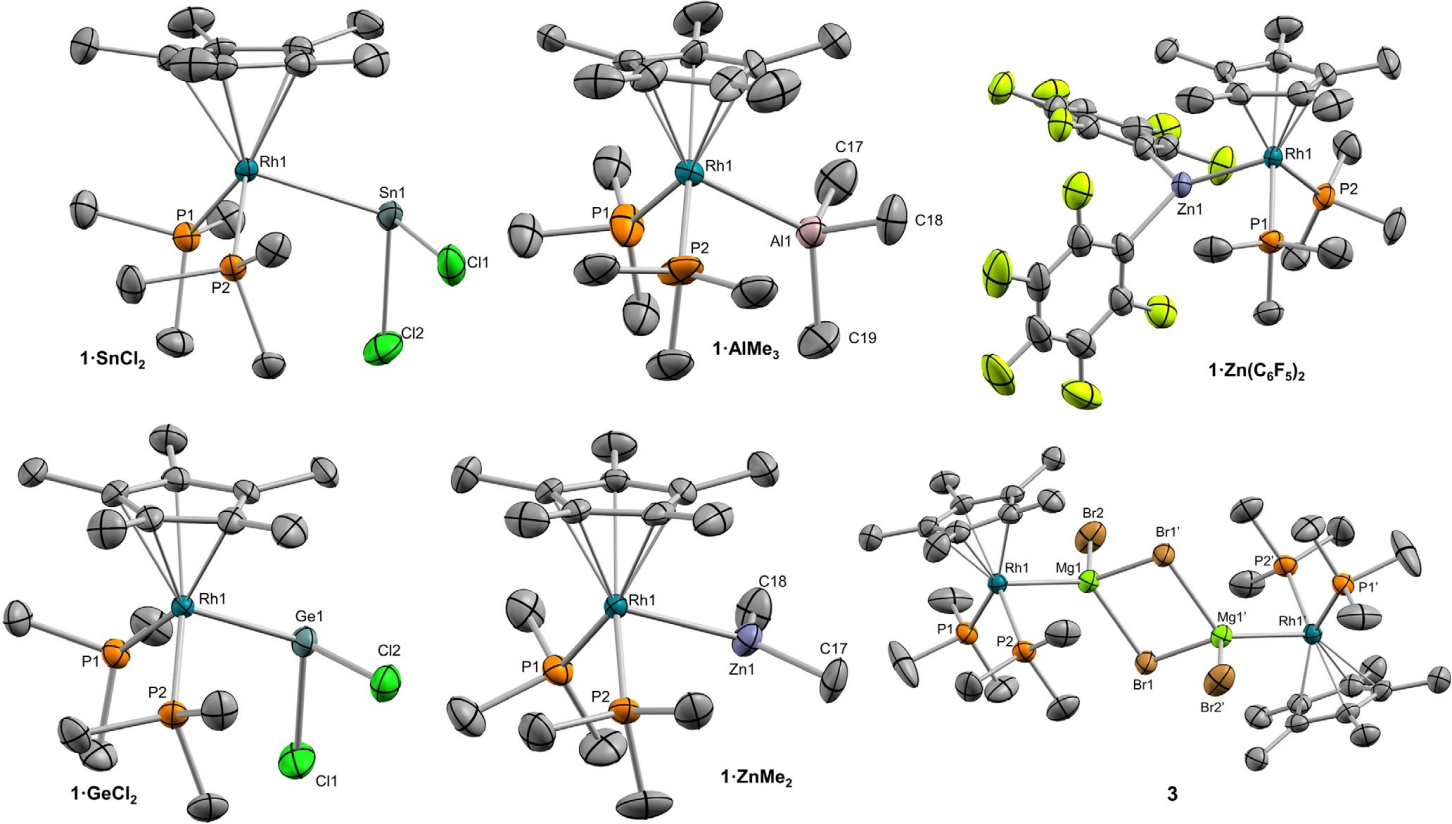
ORTEP diagram of compounds **1⋅SnCl_2_**, **1⋅AlMe_3_**, **1⋅Zn(C_6_F_5_)_2_**, **1⋅GeCl_2_**,**1⋅ZnMe_2_** and **3**; for the sake of clarity hydrogen atoms and solvent molecules are excluded, while thermal ellipsoids are set at 50 % probability.

As noted earlier we aimed to access MOLPs by combining neutral fragments, aside from the prior Li^+^ and Na^+^ exceptions, to reduce the electrostatic component of the metal‐to‐metal bond. Reaction of **1** with two equivalents of the Grignard reagent MgMeBr readily yielded a new species **3** (Scheme 1 b) characterized by a sharp decrease of the ^1^ *J*_PRh_ coupling constant to 172 Hz, along with shifts of the ^31^P{^1^H} (*δ*=−10.2 ppm) and pentamethylcyclopentadienyl ^1^H (*δ*=1.87 ppm) NMR signals towards lower frequencies. Despite the high instability of **3**, single crystals suitable for X‐ray diffraction studies were grown from diluted benzene solutions and revealed the dimeric structure [(*η* ^5^‐C_5_Me_5_)(PMe_3_)_2_Rh→Mg(Me_x_Br_1−*x*_)(*μ*‐Br)]_2_ (Figure 2) in which the methyl group bound to magnesium is mostly exchanged by a bromide nucleus^[36]^ (Me:Br with 15:85 occupancies). Using an equimolar amount of the Grignard reagent did not provide full conversion of **1**, whereas the addition of MgBr_2_ or MgMe_2_ to access a MOLP without substitutional disorder proved unsuccessful, partly due to solubility issues.

As expected, MOLP **3** adopts a piano‐stool conformation after coordination of the Lewis acid. The Rh−Mg bond length accounts for 2.651(3) Å, shortened by ca. 0.2 Å with respect to the sum of the covalent radii (2.83 Å),^[30]^ thus indicative of bond covalency (vide infra). Two other parameters, namely *d*_rel_ ^[7]^ (0.94) and fsr (formal shortness ratio)^[37]^ (1.01) (Table 1), defined as the ratio between the M−M bond distance and the sum of either the covalent radii or the metallic radii, respectively, underpin this assumption. The most relevant geometric parameters for the X‐ray diffraction structures reported in this work are depicted in Table 1. It is worth of note that this exotic structure is the first unambiguous example of an unsupported Rh−Mg bond, since the only prior related example contains a metal hydride that exhibits some degree of bridging character.^[38]^ Moreover, despite the extensive use of Grignard reagents in organometallic chemistry, it is surprising that compound **3** seems to be the only Mg‐based MOLP comprised of neutral fragments.^[39]^

**Table 1:** Selected structural parameters obtained from X‐ray diffraction studies.

MOLP	*d*_RhM_ [Å]	Σ(*r*_cov_)^[a]^ [Å]	*d*_rel_^[b]^	fsr^[c]^	*d*_RhP_^[d]^ [Å]	*d*_RhCp*_^[e]^ [Å]	PRhP [°]
**3**	2.651(3)	2.83	0.94	1.01	2.246(2)	1.958(7)	95.09(8)
**1⋅Zn(C_6_F_5_)_2_**	2.484(1)	2.64	0.94	1.01	2.253(6)	1.925(4)	93.13(2)
**1⋅ZnMe_2_**	2.618(1)	2.64	0.99	1.06	2.234(1)	1.950(5)	93.28(6)
**1⋅GeCl_2_**	2.501(1)	2.62	0.95	1.00	2.268(1)	1.978(5)	94.67(6)
**1⋅SnCl_2_**	2.687(3)	2.81	0.95	1.00	2.266(1)	1.968(4)	93.72(3)
**1⋅AlMe_3_**	2.635(4)	2.63	1.00	1.05	2.244(4)	1.964(4)	95.3(2)

[a] Σ(*r*_cov_)=sum of the covalent radii of the bonded metals.^[30]^ [b] *d*_rel_=ratio between *d*_Rh‐M_ and the sum of covalent radii. [c] fsr=formal shortness ratio=ratio between *d*_Rh‐M_ and the sum of metallic radii.^[37]^ [d] *d*_Rh‐P_=average Rh−P bond length. [e] *d*_Rh‐Cp*_=distance between Rh and the centroid of C_5_Me_5_.

**Figure 3:**
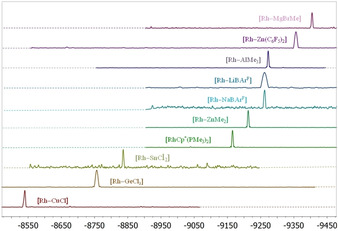
^103^Rh{^1^H} NMR spectra of **1** and Rh‐based MOLPs obtained from cross polarization experiments (HMQC). Dotted lines added to guide de eye.

As stated above, the choice of rhodium as the Lewis base was in part made attending to its NMR activity (Table 2). To observe chemical shifts associated to ^103^Rh centers we employed a cross polarization approach by means of HMQC experiments through its coupling to ^31^P nuclei (see Experimental Section for details). Considering its low sensitivity and rather wide chemical shift range (ca. 12 000 ppm),^[40]^ this strategy enormously facilitates the acquisition of ^103^Rh NMR data. The new MOLPs are characterized by ^103^Rh{^1^H} NMR resonances shifted to lower frequencies compared to precursor **1** (−9165 ppm), with **3** exhibiting a signal at −9404 ppm and the products derived from the addition of alkali metals resonating at around −9262 ppm (Figure 3).

**Table 2:** Selected NMR spectroscopic data.

Compound	^1^H, *δ* (C_5_Me_5_)	^1^H, *δ* (PMe_3_)	^1^ *J*_PRh_ [Hz]	^31^P{^1^H}, *δ*	^103^Rh{^1^H},^[a]^ *δ*
**1**	2.16	1.30	216	−7.3	−9165
**[A⋅Li]**	1.61	1.11	130	−3.0	−9261
**[A⋅Na]**	1.67	1.19	138	−3.1	−9262
**3**	1.87	1.38	172	−10.2	−9404
**1⋅Zn(C_6_F_5_)_2_**	1.59	1.06	167	−7.2	−9355
**1⋅ZnMe_2_**	1.76	1.09	192	−6.9	−9212
**1⋅GeCl_2_**	1.67	1.55	171	−7.0	−8756
**1⋅SnCl_2_**	1.67	1.56	169	−8.5	−8836
**1⋅AlMe_3_**	1.67	1.10	181	−6.9	−9272
**1⋅CuCl**	1.66	1.48	144	−3.0	−8540

[a] ^103^Rh NMR data referenced to Rh(acac)_3_.

### Synthesis of Rh^I^ MOLPs with *p*‐Block Acids

**Scheme 2:**
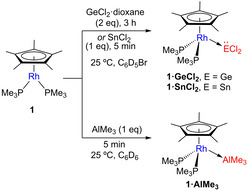
Synthesis of Rh^I^ MOLPs with tetrylenes dihalides and AlMe_3_.

Moving to the *p*‐block we examined the reactivity of **1** with widely used metalloid precursors of the group 13 and 14, more precisely GeCl_2_⋅dioxane, SnCl_2_, GaCl_3_, AlCl_3_ and AlMe_3_. Whereas tricoordinated group 14 species has been widely exploited as Lewis acids, heavier tetrylenes (i.e. :GeCl_2_, :SnCl_2_) exhibit ambiphilic behavior due to the joint presence of a lone electron pair and an empty *p* orbital. We thought of interest to access both types of MOLPs to later provide a comparison of the bonding scheme between each other. Reaction of **1** with either GaCl_3_ or AlCl_3_ resulted in the precipitation of a highly insoluble material or the formation of intractable mixtures, respectively. The latter is not surprising considering previously reported difficulties to access Rh–alane MOLPs by direct combination of the two metal fragments.^[41]^ However, addition of one equivalent of AlMe_3_ (toluene solution, 1 m) to a benzene solution of **1** resulted in clean formation of the corresponding **1⋅AlMe_3_** MOLP. The same occurs by adding GeCl_2_⋅dioxane or SnCl_2_ to bromobenzene solutions of the rhodium precursor to yield **1⋅GeCl_2_** and **1⋅SnCl_2_**, respectively, though the former required three hours for completion while the tin MOLP formed immediately. In the case of germanium, two equivalents of GeCl_2_⋅dioxane were required to achieve full consumption of **1**, presumably because the second germanium may facilitate dioxane withdrawal from the coordinating GeCl_2_ terminus (Scheme 2).

Multinuclear NMR spectroscopic analysis illustrates the formation of the new MOLPs exhibiting the same distinctive features commented above (Table 2), that is, a marked decrease of the ^1^ *J*_PRh_ coupling constant of ca. 40 Hz and a displacement to lower frequencies of the ^1^H NMR signal due to the pentamethylcyclopentadienyl ring. For the tin analogue we could also detect a broad ^119^Sn{^1^H} NMR signal at 810.7 ppm, whereas **1⋅AlMe_3_** provides a distinctive ^1^H NMR singlet at −0.1 ppm due to the Al‐bound methyl termini, with a corresponding ^13^C{^1^H} NMR signal at 1.0 ppm. Interestingly, ^103^Rh{^1^H} NMR resonances due to the tetrylene MOLPs appear upshifted by ca. 400 ppm (*δ*=−8756, **1⋅GeCl_2_**; −8836 ppm, **1⋅SnCl_2_**) compared to **1** (*δ*=−9165 ppm), contrasting with all other main‐group based MOLPs reported herein (Table 2).

Single‐crystals of compounds **1⋅GeCl_2_**, **1⋅SnCl_2_** and **1⋅AlMe_3_** amenable to X‐ray diffraction studies where grown by slow diffusion of pentane into their benzene or bromobenzene solutions, once more revealing the piano stool configuration around the rhodium center after coordination to the Lewis acids (Figure 2, Table 1). The unsupported M−M bond lengths for **1⋅GeCl_2_** (2.501(1) Å) and **1⋅SnCl_2_** (2.687(3) Å) are slightly shorter than the sum of covalent radii (*r*_Rh+Ge_=2.62; *r*_Rh+Sn_=2.81 Å),^[30]^ whereas that of **1⋅AlMe_3_** (2.635(4) Å) is identical to the expected theoretical value for a covalent interaction (2.63 Å).^[30]^ The asymmetric unit of structure **1⋅GeCl_2_** contains four independent molecules of the MOLP, being the aforementioned Rh‐Ge bond length the average for all of them. The solid‐state structures of **1⋅GeCl_2_** and **1⋅SnCl_2_** unveil a strong pyramidalization of the tetrel moiety, as seen in other related systems based on platinum.^[42]^ However, this is not the case in other metallic complexes with bound tetrels and a planar disposition around the group 14 element.^[43]^ It has been noticed that pyramidalization requires both coordination to strongly Lewis basic metals and a non‐directional lone pair,^[42d]^ features fulfilled for **1⋅ECl_2_** (E=Ge, Sn). Since the lone pair on stannylene dichloride has more pronounced *s*‐character than that in its germylene analogue, the directionality of the former is decreased and as such a higher pyramidalization is anticipated for **1⋅SnCl_2_**. In fact, the pyramidalization angle estimated by the POAV method of Haddon^[44]^ for **1⋅SnCl_2_** (26.2) surpass that of **1⋅GeCl_2_** (24.4).

To the best of our knowledge, compounds **1⋅GeCl_2_** and **1⋅SnCl_2_** represent the first examples of rhodium‐bound germylene and stannylene non‐stabilized by the coordination of a base. All prior structures containing Rh−E(II) (E=Ge, Sn) bonds involve tetrel centers bearing an additional intra‐ or intermolecular Lewis donor.^[45]^ As such, those escape the definition of MOLP investigated in this work, since base‐stabilized tetrylenes do not behave as acidic fragment any more, but as σ‐donating ligands. For its part, earlier reports describe base‐free rhodium adducts of SnCl_2_, but their dimeric nature preclude a clear understanding of the bonding situation.^[46]^ As introduced earlier, the preparation of a Rh‐alane adduct by direct combination of the two metal fragments, as reported herein, had so far been unsuccessful. The first crystallographycally characterized Rh–alane adduct was reported by Braunschweig relying on the transmetalation of the alane from [(PCy_3_)_2_Pt→AlCl_3_] to [(*η* ^5^‐C_5_H_5_)Rh(PMe_3_)_2_].[^47^, ^48^] The Rh−Al bond length in **1⋅AlMe_3_** is considerably elongated by around 0.2 Å relative to the two previously reported Rh–alane adducts based on AlCl_3_,[^41^, ^47^] as expected for the less acidic AlMe_3_. This diminished acidity may explain the absence of previous unsupported transition metal MOLPs containing trimethylaluminum, being **1⋅AlMe_3_** the first of its kind.^[49]^ Once more, this is an unexpected finding considering the extensive use of AlMe_3_ as a methylating agent or in transition metal catalyzed polymerization.

### Synthesis of Rh^I^ MOLPs with *d*‐Block Acids

**Scheme 3:**
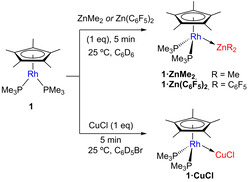
Synthesis of Rh^I^ MOLPs with electrophiles ZnMe_2_, Zn(C_6_F_5_)_2_ and CuCl.

Turning into the *d*‐block and keeping our aim to prepare Rh^I^ MOLPS with neutral main group metal Lewis acids we decided to check the reactivity of **1** with two common zinc precursors, more precisely ZnMe_2_ and Zn(C_6_F_5_)_2_. For the sake of completeness, we also examined the formation of metal adducts with simple forms of copper and silver. Complexes **1⋅ZnMe_2_** and **1⋅Zn(C_6_F_5_)_2_** were immediately formed after addition of one equivalent of the organometallic zinc substrate over a benzene solution of **1** (Scheme 3). These complexes exhibit sharp ^31^P{^1^H} NMR signals at *δ*=−6.9 (^1^ *J*_PRh_=192 Hz) and −7.2 ppm (^1^ *J*_PRh_=167 Hz), respectively. The noticeable decrease of the ^1^ *J*_PRh_ coupling constants relative to **1** evidences formation of Rh→Zn MOLPs. Their corresponding ^103^Rh{^1^H} NMR resonances appear downshifted to −9212 (**1⋅ZnMe_2_**) and −9355 (**1⋅Zn(C_6_F_5_)_2_**) ppm. Other relevant NMR spectroscopic parameters are collected in Table 2 and in the Experimental Section.

Reaction with group 11 precursors, whose acidity is also well‐recognized, proved more problematic. Reaction with CuOTf (OTf^−^=CF_3_SO_3_^−^) or AgNTf_2_ (NTf_2_^−^=(CF_3_SO_2_)_2_N^−^) resulted in complex mixtures that involve a number of rhodium compounds as inferred from the presence of several doublets in the corresponding ^31^P{^1^H} NMR spectra. In contrast, addition of one equivalent of CuCl over a bromobenzene solution of **1** cleanly provided a new species (**1⋅CuCl**) characterized by a ^31^P{^1^H} NMR doublet at −3.0 ppm (^1^ *J*_PRh_=144 Hz), once again suggesting the formation of a dative bond between the two metals (Scheme 3). The corresponding ^103^Rh{^1^H} signal resonates at −8540 ppm, shifted to higher frequencies compared to **1**. This contrasts with all other MOLPs described herein except those containing ambiphilic tetrylenes, which speaks in favor of some differences in the bonding situation between the MOLPs involving purely acidic fragments and those where some degree of back‐donation may be anticipated (i.e. those based on Ge, Sn and Cu).

Crystals of **1⋅ZnMe_2_** and **1⋅Zn(C_6_F_5_)_2_** where grown by slow diffusion of pentane into their benzene solutions. The larger acidity of the fluorinated zinc moiety is reflected in a shorter Rh‐Zn bond length of 2.484(1) Å in **1⋅Zn(C_6_F_5_)_2_** compared to that in **1⋅ZnMe_2_** (*d*_RhZn_=2.618(1) Å), attesting as well that steric effects may be less relevant (Figure 2). Nonetheless, both Rh−Zn distances account for less than the sum of the corresponding covalent radii (2.64 Å),^[30]^ suggesting a strong metal–metal interaction. These two complexes constitute the first unsupported MOLPs exhibiting a dative Rh→Zn bond and constructed around neutral fragments.[^38^, ^50^] Structures alike these are presumably relevant intermediates during Rh^I^‐catalyzed Negishi coupling reactions.[^50e^, ^51^] Mechanistic studies have permitted to isolate a Rh/Zn complex derived from insertion of the rhodium center into one of the Zn−C bonds in diphenylzinc,^[51b]^ whose likely precursor consist in a Lewis adduct akin to **1⋅ZnMe_2_** or **1⋅Zn(C_6_F_5_)_2_**. Related to this, formation of a [Rh^I^]→ZnCl_2_ MOLP was postulated as a deactivation product during catalysis, although their molecular formulation could not be elucidated.

Regarding the copper adduct, attempts to grow single crystals of **1⋅CuCl** were unfruitful, partly because of the low solubility of the adduct which caused rapid precipitation in most cases. This fact, along with non‐definitive diffusion spectroscopic studies, prevented us to obtain a clear picture of its molecular structure. In principle, both a monomeric or dimeric nature could be proposed. To discern between these two possibilities, we made use of DFT calculations. However, attempts to optimize a dimeric species of type [(*η* ^5^‐C_5_Me_5_)Rh(PMe_3_)_2_Cu(*μ*‐Cl)]_2_ resulted in cleavage of the chloride bridges, supporting an unbridged formulation for **1⋅CuCl**. It is interesting to note that this species represents a rare case of Rh→Cu MOLP, with prior complexes bearing a Rh−Cu bond typically relying on the stability conferred by bridging ligands,^[52]^ the use of cationic copper fragments^[53]^ or the coordination of the neutral copper halide as a bridging motif.^[54]^

### Computational analysis of Rh→M bonding in Rh^I^ MOLPs

Insight into the nature of the Rh→M interactions in the Rh^I^−M adducts has been obtained from DFT calculations, analysis of the calculated electron densities of the adducts within the Atoms In Molecules theory (AIM)^[55]^ and Natural Bonding Orbitals (NBO) analysis.[^56^, ^57^, ^58^] Optimized geometries of the adducts in bulk solvent were obtained by DFT methods (SMD‐ωB97XD/6‐31 g(d,p)/SDD level)[^59^, ^60^, ^61^, ^62^, ^63^, ^64^] with the Gaussian09 software.^[65]^ Although it can be argued that DFT‐optimized geometries with a solvent model may not represent appropriately the solid state structures, it must be highlighted that our model is in good agreement with the X‐ray diffraction geometries available (RMSD for all geometries is 0.58 Å) and particularly that the calculated Rh−M distances remain equal or below the sum of the covalent radii of the two atoms.[^7^, ^29^] Optimized geometries for the Na, Li and Cu adducts were also calculated in halogenated benzene. In the case of the Li and Na species, the BAr_F_^−^ anion was excluded from the calculations to yield Rh−M distances of 2.46 and 2.76 Å respectively. When the BAr_F_^−^ was introduced in the Na system, the Rh−Na distance increased only slightly to 2.77 Å, still shorter than the sum of the covalent radii of Rh and Na. The CuCl adduct was considered as a monomeric species and the calculations afforded a Rh−M distance of 2.37 Å (∑_cov radii_=2.74 Å).

**Figure 4:**
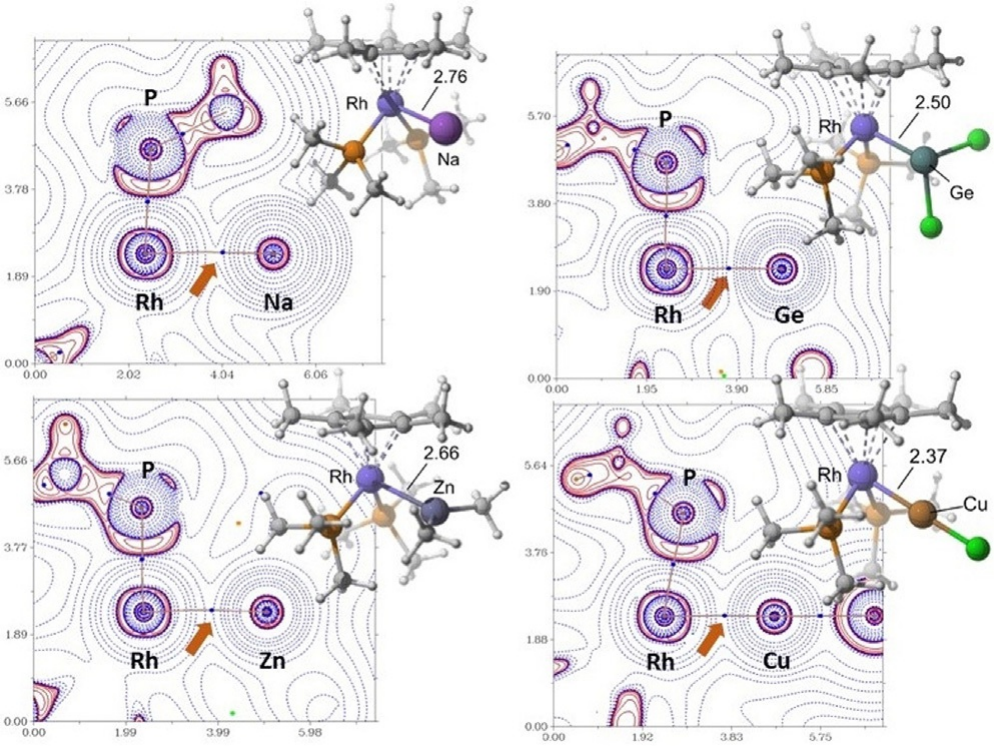
BCPs (blue dots) and bond paths (orange trace) of the electron density of **1⋅Na** and **1⋅GeCl_2_**, **1⋅ZnMe_2_** and **1⋅CuCl** superimposed on the function *L*=−∇^2^ *ρ_b_* in one of the M‐Rh‐P planes. The orange arrows point to the Rh−M BCPs. Dotted blue and solid red contour lines are for positive and negative values of *L*. The optimized geometries of the adducts are also shown.^[78]^ Distances are in Å.

Topological analysis of the electron density was carried out with the AIM methods and the Multiwfn software[^66^, ^67^] from wavefunctions calculated at the SMD‐ωB97XD/6‐311++g(2d,p)/Sapporo‐TZP level[^68^, ^69^, ^70^, ^71^, ^72^] with the previously optimized geometries. This study located bond critical points (BCPs) in the electron density and unique bond paths connecting the Rh and M atoms for all adducts (Figure 4 and SC1).

The existence of BCP and bond paths between two atoms has been interpreted as the necessary condition for them to form a chemical bond and several indicators based on the electron density have been used in the literature to characterize interatomic interactions.[^55^, ^73^] Namely, the Laplacian of the electron density at the BCP, ∇^2^ *ρ_b_*, and the total energy density, *H_b_*, as the sum of the electronic potential and kinetic energy densities, *G_b_* and *V_b_*. Thus, for open‐shell interactions (pure covalent bonds) ∇^2^ *ρ_b_*<0 (the electron density is locally concentrated) and for closed‐shell interactions ∇^2^ *ρ_b_* >0 (the electron density is locally depleted). Closed‐shell interactions are also characterized by electron densities at the BCPs, *ρ_b_*, of the order of 0.01 a.u., at least one order of magnitude smaller than in open‐shell interactions. Moreover, it has been argued that the sufficient condition for a bond to be considered covalent is *H_b_*<0, independently of the sign of the Laplacian.[^74^, ^75^] A class of intermediate or partially covalent bonds^[76]^ have thus been characterized as having 2>|*V_b_*|/*G_b_*>1. Shared (metal–metal) and donor–acceptor (metal–ligand) interactions fall within this class.^[77]^

**Figure 5:**
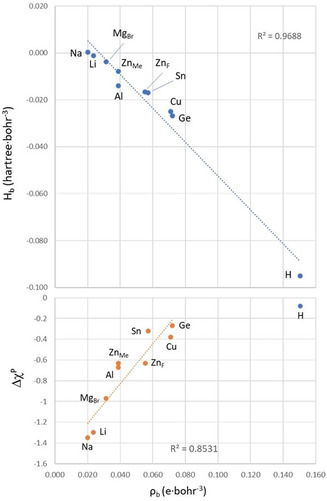
Correlation of the total energy density, and electron density, *ρ_b_*, at Rh−M BCPs of the Rh^I^−M adducts and the Rh−H BCP of **2** (above); and correlation of the Pauling electronegativity difference (Δ*χ* ^p^=*χ* ^P^ (M or H)−*χ* ^P^ (Rh)) with the electron density, *ρ_b_*, at Rh−M BCPs (below).

As shown in Table 3 (and Table S3 in the Supporting Information), the values of *ρ_b_* for our Rh−M interactions are small, ranging from 0.020 a.u. for **1⋅Na** to 0.072 a.u. for **1⋅GeCl_2_**. This, in addition to positive values for ∇^2^ *ρ_b_*, is in agreement with closed‐shell interactions between the Rh and M atoms.^[55]^ For the sake of comparison, Rh−P BCPs’ have *ρ_b_* values close to 0.1 a.u and ∇^2^ *ρ_b_* >0. Also, *ρ_b_* at the Rh−H bond of the Rh^III^ hydride **2** has a value of 0.150 a.u. and ∇^2^ *ρ_b_* >0. Arguably,^[24a]^ the magnitude of *ρ_b_* and *H_b_* can be used to assess the strength of an interaction.^[79]^ In this case, *ρ_b_* follows the order Na^+^<Li^+^<MgBr_2_<ZnMe_2_≈AlMe_3_<Zn(C_6_F_5_)_2_≈SnCl_2_<CuCl≈GeCl_2_≪H, and it correlates with *H_b_*,^[80]^ which interestingly, is negative for all species except for that with the smallest *ρ_b_*, **1⋅Na** (Figure 5).

**Table 3:** QTAIM indicators at Rh^I^−M BCPs. All data are in atomic units. electron density, *ρ*_b_ (*e*⋅bohr^−3^); total energy density *H_b_* (hartree⋅bohr^−3^); Laplacian of the electron density ∇^2^ *ρ_b_* (*e*⋅bohr^−5^); ratio between the absolute electronic potential energy and kinetic energy densities |*V_b_*|/*G_b_* ; delocalization index between Rh and M atoms, *δ*(Rh,M) [*e*].

		*ρ*_b_	*H_b_*	∇^2^ *ρ_b_*	|*V_b_*|/*G_b_*	*δ*(Rh,M)
	**Li^+^**	0.024	−0.001	0.078	1.059	0.099
***s***	**Na^+^**	0.020	0.000	0.070	0.981	0.142
	**MgBr_2_** ^[a]^	0.0316	−0.003	0.095	1.120	0.224
	**AlMe_3_**	0.039	−0.014	0.026	1.683	0.260
***p***	**GeCl_2_**	0.072	−0.027	0.022	1.830	0.851
	**SnCl_2_**	0.057	−0.017	0.037	1.649	0.775
***d***	**ZnMe_2_**	0.039	−0.008	0.073	1.300	0.370
	**Zn(C_6_F_5_)2**	0.055	−0.017	0.069	1.437	0.545
	**CuCl**	0.071	−0.025	0.150	1.399	0.639

[a] Calculations at the SMD‐ωB97XD/6‐311+g(d,p)/def2‐TZVP(ECP) level (numerical values do not vary much when this level of theory was applied to other adducts).

These results suggest that the least electronegative atoms (Li, Na, and Mg), with the smallest *ρ_b_* and *H*_b_ close to zero, form predominantly ionic interactions with Rh (although with some degree of electron sharing as it shall be discussed below), whereas the covalent character becomes more prominent as the electronegativity of the element bound to Rh increases and their electronegativity difference decreases (Δχ^p^=χ^P^ (M or H)–χ^P^ (Rh)). Indeed, reasonable correlations have been found between Δχ^p^ and *ρ_b_* or *H_b_* as shown in Figure 5 for *ρ_b_* (and Figure S4 for *H_b_*). These correlations highlight a general trend, but they obviously fail to account for the complexity of the interactions. For example, they do not reflect the different acidity of the two Zn fragments and do not include the Rh−H bond of **2**, since its associated *H_b_* relative to those of the Rh−M bonds is higher than the corresponding electronegativity difference.

Another parameter that has been considered in this study is the delocalization index between the Rh and M, or H atoms, *δ*(Rh,M), which accounts for the extent of electron sharing between the atomic basins[^81^, ^82^] and can be considered an AIM equivalent to orbital‐based bond orders. For a covalent bond, such as the H−H or C−H bonds, *δ*(C,H) is close to 1, whereas purely ionic interactions have delocalization indices close to zero. Table 3 shows *δ*(Rh,M) values stretching from less than 0.022 electrons for the adducts with s‐block metals to close to 0.8 electrons for the adducts with the two tetrylenes, attesting the higher covalent character of the latter interactions. The value calculated for the Rh−H bond of **2**, a covalent bond, is 0.91 electrons. Thus, the same trends as those emerging from *ρ_b_* and *H_b_* are observed for *δ*(Rh,M) including a linear dependence with Δ*χ* ^p^ (Figure S4).

When the Laplacian of the electron density is considered, all adducts yield positive values at the Rh−M (and Rh−H) BCPs, which is indicative of close‐shell interactions. In this case, no correlations arose between the Laplacian and other magnitudes derived from the electron density. Some correlations between the Laplacian and the electron density or the electronegativity difference have been found in coordination compounds^[79]^ and their absence in this case may reflect the different nature of the various Rh−M interactions of this work, as shall be discussed below from an orbital perspective. Nevertheless, we can classify these interactions in at least two groups according to |*V*_b_|/*G*_b_ values (vide supra). One includes the adducts with *s*‐block metals, which have |*V*_b_|/*G*_b_ values close to 1 (*H_b_*≈0), characteristic of interactions with very low covalent character (for **1⋅Na** |*V*_b_|/*G*_b_ =0.98 a.u.), and a second group contains the remaining adducts with *p*‐ and *d*‐block metal, with |V_*b*_|/G_*b*_ values that range from 1.30 a.u. for **1⋅ZnMe_2_** to 1.83 a.u. for **1⋅GeCl_2_**, typical of more covalent, intermediate interactions. For the sake of comparison Rh←P bonds in these systems, classical donor–acceptor interactions, have associated |V_*b*_|/G_*b*_ values of about 1.5–1.6 a.u. The higher values for |*V*_b_|/*G*_b_ have been found for the three *p*‐block metals, with the value for the Rh−Ge interaction approaching the |*V*_b_|/*G*_b_ ≥2 (∇^2^ *ρ_b_*≤0) limit for shared‐shell (pure covalent) interactions. The |*V*_b_|/*G*_b_ and ∇^2^ *ρ_b_* values for the Rh−H bond of **2** are 1.975 and 0.010 a.u. respectively.

Natural bonding orbital (NBO) analysis was performed at the DFT SMD‐ωB97XD/6–311 g(2d,p)/def2‐TZVP(ECP) level.[^83^, ^84^] The NBO method creates a pattern of localized bonds and lone pairs that is a Lewis‐type description for the molecule. These natural bonding orbitals may not achieve double occupancy, and the departures from the “electron pair” can be rationalized in terms of partial occupation of “non‐Lewis” orbitals and donor–acceptor interactions between molecular fragments. Each NBO can be associated with a natural localized molecular orbital (NLMO), which is exactly doubly occupied and results from incorporation of mixings with non‐Lewis orbital.^[85]^ Thus, when an NBO is identified as the donor orbital in an interaction, the corresponding NLMO informs about the degree of mixing with the acceptor orbital. In addition, donor–acceptor stabilization energies (Δ*E_ij_*) can be calculated that are related to the strength of the interaction.

**Figure 6:**
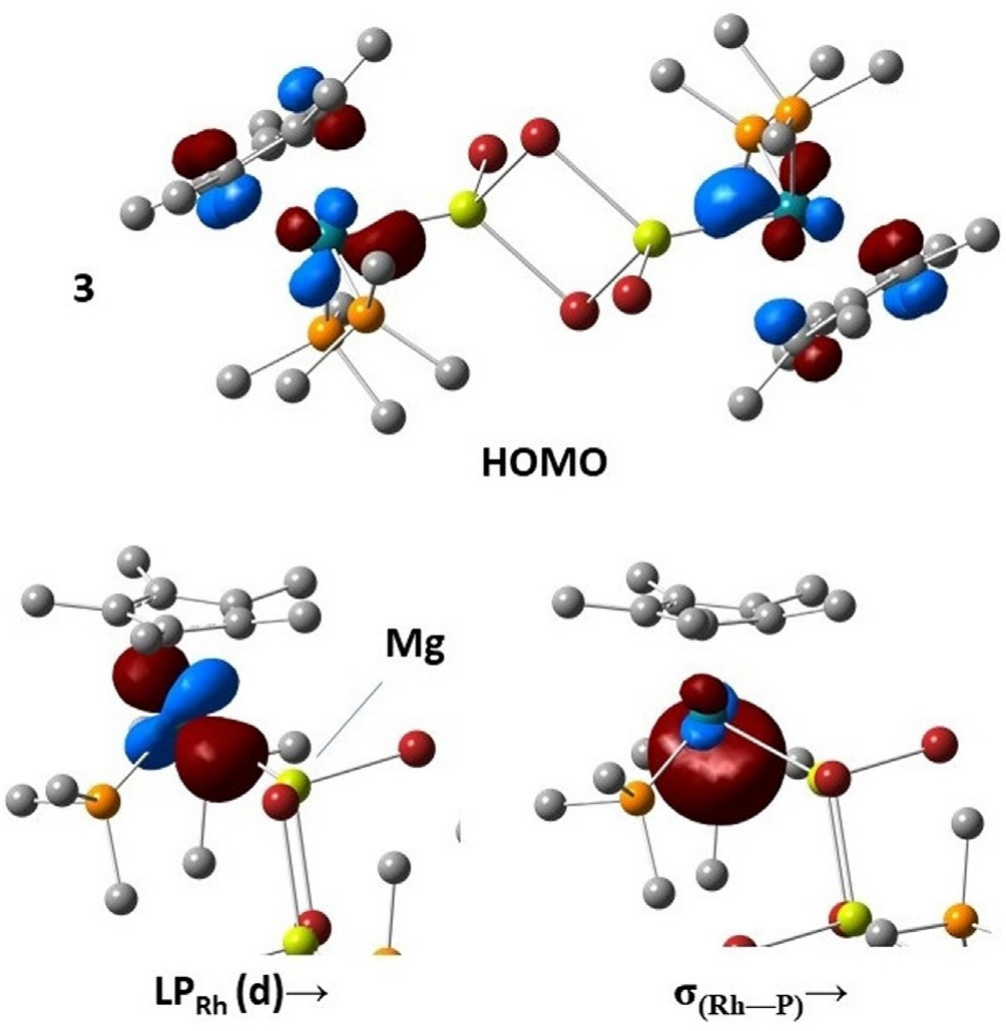
HOMO (0.05 a.u. isosurface), and one LP_Rh_ and σ_(Rh‐P)_ NBO (0.06 a.u. isosurface) involved as donors in donor‐acceptor interactions with one of the Mg atoms (yellow sphere) of **3**.

**Figure 7:**
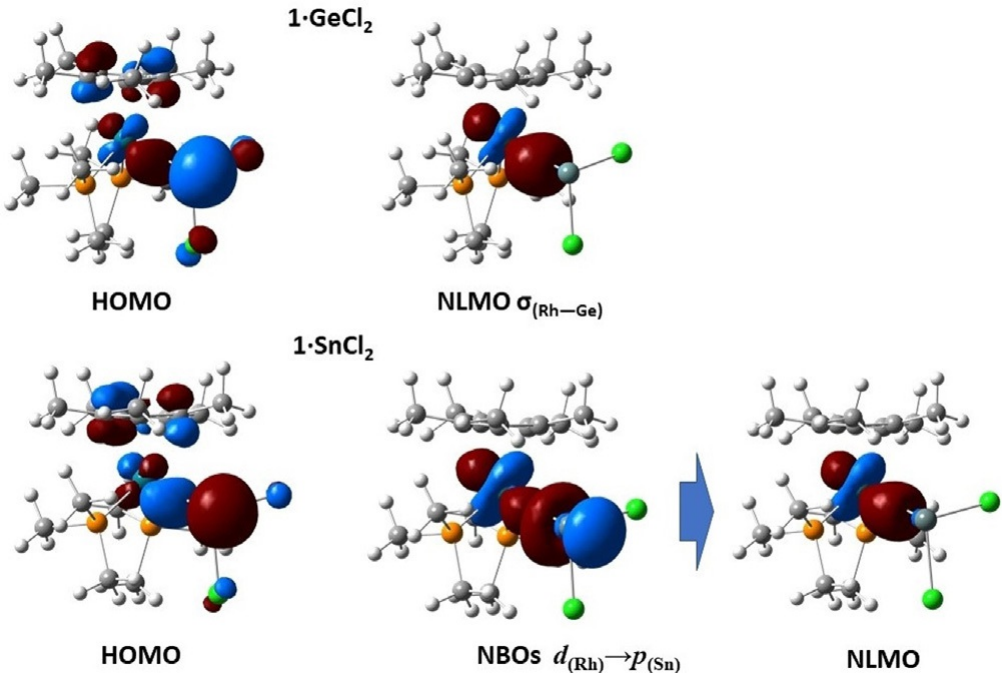
HOMOs (0.06 a.u. isovalue), and localized orbitals (0.05 a.u. isovalue) relevant to the Rh–tetrylene interactions in **1⋅GeCl_2_** and **1⋅SnCl_2_**. Notice the orbital mixing in the HOMOs compared to Figure 6.

Table 4 summarizes relevant donor–acceptor interactions and Wiberg bond orders (WBO) from the NBO analysis of the Rh^I^→M bonds. Typical NBO terminology has been used to name the different types of NBOs, such as LP for lone pair, and LV for lone vacancy, which refers to an empty valence orbital localized on one atom. Also, the main atomic orbital contribution to the LVs has been included in parenthesis. The NLMO column indicates the percentage of non‐Lewis orbitals from the acceptor atom that are mixed with the parent donor NBO. This section does not aim at being comprehensive, but to illustrate representative interactions and to offer a qualitative picture of the Rh−M bonding. For instance, more than one LP_Rh_→LV interaction has been located for most systems whereas only the most important is shown. Data for σ_Rh‐P_→LV interactions correspond to the average values of the interaction with the two Rh−P bonds in each adduct. Finally, back donation is by far one minor contribution to the Rh–tetrylene interactions, but it has been highlighted to illustrate the ambiphilic behavior of GeCl_2_ and SnCl_2_ in these adducts. Figure 6 and Figure 7 show examples of relevant NBOs and NLMOs for the above interactions.

**Table 4:** Relevant NBO results including major donor–acceptor interactions.

		WBO	Donor NBO/occupancy [*e*]	Acceptor NBO/occupancy [*e*]	Δ*E_ij_* kcal mol^−1^	NLMO
***s***	**Li^+^**	0.034	LP_Rh_ (*d*)/1.95	LV (2*s*) Li/0.05	6.0	0.39 % Li
			σ_Rh‐P_/1.90		22.5	
	**Na^+^**	0.034	LP_Rh_ (*d*)/1.95	LV (3*s*) Na/0.05	6.7	0.33 % Na
			σ_Rh‐P_/1.90		19.3	
	**MgBr_2_**	0.138	LP_Rh_ (*d*)/1.82	LV (3*s*) Mg/0.38	28.7	3.66 % Mg
			σ_Rh‐P_/1.89		41.3	0.99 % Mg
						
***p***	**AlMe_3_**	0.311	LP_Rh_ (*d*)/1.75	LV (3*sp* ^3^) Al/0.36	33.7	9.81 % Al
			σ_Rh‐P_/1.84		80.6	3.11 % Al
	**GeCl_2_** ^**[a]**^	0.514	–	–	–	–
			LP_Ge_ (*s*)/1.97	σ*_Rh‐P_/0.49	9.78	0.89 % Rh
	**SnCl_2_**	0.446	LP_Rh_ (*d*)/1.69	LV (5*p*) Sn/0.60	55.2	15.00 % Sn
			σ_Rh‐P_/1.84		62.8	2.68 % Sn
			LP_Sn_ (*s*)/1.97	σ*_Rh‐P_/0.40	10.12	0.72 % Rh
						
***d***	**ZnMe_2_**	0.097	LP_Rh_ (*d*)/1.84	LV (4*s*) Zn/0.55	15.2	2.06 % Zn
			σ_Rh‐P_/1.89		17.9	0.43 % Zn
	**Zn(C_6_F_5_)_2_**	0.204	LP_Rh_ (*d*)/1.77	LV (4*s*) Zn/0.54	41.7	5.39 % Zn
			σ_Rh‐P_/1.88		46.6	0.97 % Zn
	**CuCl**	0.210	LP_Rh_ (*d*)/1.76	LV (4*s*) Cu/0.42	28.5	6.10 % Cu
			σ_Rh‐P_/1.88		35.1	1.41 % Cu

[a] The Rh−Ge bond is not described in terms of donor–acceptor interactions (vide infra).

The NBO analysis locates 4 LPs, almost pure *d* orbitals, on the Rh atoms of all adducts, except for **1⋅GeCl_2_** and the hydride **2**, for which only 3 *d* LPs where found. This agrees with a Rh^I^ formulation and *d* ^8^ electron count for most adducts and the expected Rh^III^, *d* ^6^, formulation for the hydride. In the case of **1⋅GeCl_2_**, a Rh^III^/Ge^0^ formulation cannot be assumed. Instead, we propose that the [(*η* ^5^‐C_5_Me_5_)Rh(PMe_3_)_2_] moiety forms one dative covalent bond with GeCl_2_, the latter acting effectively as an Z‐type ligand,^[22a]^ as will be discussed in more detail later.

Inspection of the Rh *d* LPs of the formally Rh^I^ adducts shows that at least one of them is populated by 1.82 electrons or less, except for **1⋅Li** and **1⋅Na**, for which the relevant lowest occupied Rh LPs have 1.95 electrons each. The occupancy is higher for the adducts of the more electropositive elements and lower for the adducts of the more electronegative ones, with the lowest occupancy found for **1⋅SnCl_2_** at 1.69 electrons. This reflects, once more, a higher degree of electron sharing in the adducts with the electronegative atoms. These Rh LPs are delocalized onto LV NBOs of the acceptor metal atoms. Thus, for *s*‐block atoms, the acceptor LV is mostly a valence s orbital, and the corresponding interaction can be described as *d*_(Rh)_→*s*_(M)_. The occupancy of the acceptor orbital and the major donor‐acceptor stabilization (or delocalization) energies (Δ*E_ij_*) for these interactions are: 0.05 electrons and 6.00 kcal mol^−1^ for **1⋅Li**; 0.05 electrons and 6.71 kcal mol^−1^ for **1⋅Na**; and 0.40 electrons and 28.7 kcal mol^−1^ for the MgBr_2_ adduct, **3**.

However, in the above species, as well as in the remaining adducts considered, the Rh→M interaction is dominated, at least in terms of delocalization energies, not by Rh‐localized *d* orbitals, but by electron donation from the σ_(Rh‐P)_ bonds,^[31b]^ which have about 72 % P (*sp*) and 28 % Rh (*sd*) character. For the Li, Na and MgBr_2_ adducts the σ_(Rh‐P)_→*s*_(M)_ interaction have Δ*E_ij_* of 22.5, 19.3, 41.3 kcal mol^−1^ respectively.

The NBO description of the Rh−M bonding in the adducts with *p*‐block acceptor atoms is more varied than above. Thus, *d*_(Rh)_→*sp* ^*3*^_(Al)_ and σ_(Rh‐P)_→*sp* ^*3*^_(Al)_ donor–acceptor interactions were located for **1⋅AlMe_3_**, with the latter being the major contribution in terms of delocalization energy (Δ*E_ij_* are 33.7 and 80.6 kcal mol^−1^ respectively). The occupancies of the donor NBOs are 1.75 and 1.84 electrons for the Rh LP and the Al LV, a valence *sp* ^3^ hybrid, respectively. The Rh−M interactions in the adducts with the two tetrylenes, **1⋅SnCl_2_** and **1⋅GeCl_2_**, which were assigned the highest covalent character according to the AIM analysis, are described very differently by the NBO analysis: whereas the donor–acceptor description is used for the former, one bonding NBO was localized between Rh and Ge in the latter (Figure 7). Close inspection of the NLMO associated with the donor NBO of the *d*_(Rh)_→*p*_(Sn)_ interaction in **1⋅SnCl_2_** (Δ*E_ij_*=55.2 kcal mol^−1^) reveals that it has the highest mixing of acceptor metal orbitals of all analogous NLMOs in this study, with 81.9 % Rh and 15 % Sn composition,^[86]^ whereas the NLMO associated with the σ_(Rh‐Ge)_ NBO of **1⋅GeCl_2_** has an even higher mixing of Ge orbitals, although it is heavily weighted towards the Rh atom: 71 % Rh (s*d* ^*2*^) and 23 % Ge (*p*), with about 2 % mixing from each P atom. This can be compared with the σ_(Rh‐H)_ NBO of **2**, which has about 55 % Rh character and 45 % H character. The bonding in this case is pure covalent from the localized orbital perspective. Nevertheless, the σ_(Rh‐P)_→*p*_(Sn)_ interaction is also dominant in **1⋅SnCl_2_**,^[31b]^ with Δ*E_ij_*=62.8 kcal mol^−1^. The involvement of the Rh−P bonds in the Rh−Ge interaction of **1⋅GeCl_2_** is described in terms of donor–acceptor interactions: σ_(Rh‐Ge)_→σ*_(Rh‐P)_ and σ*_(Rh‐Ge)_←σ_(Rh‐P),_ and in the mixing of P orbitals in the NLMO associated to the σ_(Rh‐Ge)_ NBO. According to these results, the interaction in **1⋅GeCl_2_** is best described as a dative covalent bond with the Rh fragment acting as an L ligand, and a similar description, with a lower degree of electron donation/sharing, could be used for **1⋅SnCl_2_**, that is, both can be equally described as MOLPs.

In addition, it is interesting to note that both tetrylenes have LPs which are mostly filled valence *s* orbitals, which back donate electron density onto antibonding σ*_(Rh‐P)_ NBOs.^[31b]^ Back‐donation to the [(*η* ^5^‐C_5_Me_5_)Rh(PMe_3_)_2_] is a minor contribution to the Rh−Ge and it es negligible, when detected (Δ*E_ij_*≤1 kcal mol^−1^), in the remaining cases.

In the adducts with *d*‐block acceptor atoms, the donor‐acceptor interaction description has also been chosen. The Zn and Cu atoms of **1⋅ZnMe_3_**, **1⋅Zn(C_6_F_5_)_2_** and **1⋅CuCl** accept electron density onto their 4s valence orbitals from Rh LP (*d*) and σ_(Rh‐P)_ NBOs. The degree of interaction, based on bond order, occupancy of the donor and acceptor orbitals and donor–acceptor stabilization energies is intermediate between those of *s*‐block metals containing adducts and those of *p*‐block metal containing adducts, for which is greatest.

**Figure 8:**
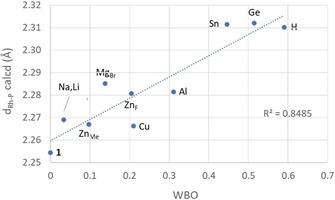
Calculated Rh−P distances versus Rh−M(H) Wiberg bond orders.

The magnitude of the Rh−M orbital interactions is reflected in the WBOs, which roughly follow the same trends as *ρ_b_* and *δ*(Rh,M), described above. Interestingly, some of these trends can be used to explain, at least qualitatively the variation of the Rh−P distances, which are shorter for adducts with smaller WBOs for their Rh−M bonds (or *ρ_b_* and *δ*(Rh,M)) and longer for adducts with larger WBOs (Figure 8). As the Rh^I^→M interactions become more important, there is a greater involvement of σ_(Rh‐P)_ orbitals (and in some cases weak back donation onto σ*_(Rh‐P)_), therefore weakening the Rh−P bonds.

## Conclusions

The choice of [(*η* ^5^‐C_5_Me_5_)Rh(PMe_3_) as a Lewis base for the synthesis of unsupported MOLPs has proved highly successful. We have prepared up to nine Rh‐based bimetallic compounds of this kind, providing X‐ray diffraction structures for those containing fragments MgMeBr, Zn(C_6_F_5_)_2_, ZnMe_2_, GeCl_2_, SnCl_2_ and AlMe_3_. It is surprising that despite the wide use of some of these Lewis acidic fragments, their corresponding MOLPs represent highly unusual examples of unsupported M−M bonding, particularly in cases like those with a Rh→Mg (**3**) or a Rh→Al (**1⋅AlMe_3_**) dative bonds. The growing interest on MOLPs is reflected by increased number of studies focusing either on accessing new structures or computationally investigating families of compounds already prepared, whereas a combined effort on a series of MOLPs is still lacking. We provide here a comprehensive computational investigation on the Rh−M bonding of the prepared Rh MOLPs, with several sound correlations found for relevant parameters associated to the metal‐to‐metal bond. For instance, the more electronegative atoms (Ge, Sn, Al) tend to form more covalent bonds with rhodium, whereas the ionic character becomes more prominent in the least electronegative (Li, Na, Mg). Nevertheless, we have quantified some degree of electron sharing for all investigated MOLPs. Curiously, the Rh→M bond is dominated by electron donation from the Rh−P σ‐bonds rather than from a filled Rh *d*‐orbital to the acidic site, which results in other relevant correlation between Wiberg Bond Orders and Rh−P bond lengths. Overall, we believe that this combined experimental/computational approach to Rh‐based MOLPs will aid in the development of other related systems for the advancement of this growing field.

## Experimental Section

**General considerations**: All preparations and manipulations were carried out using standard Schlenk and glove‐box techniques, under an atmosphere of argon and of high purity nitrogen, respectively. All solvents were dried, stored over 4 Å molecular sieves, and degassed prior to use. Toluene (C_7_H_8_) and *n*‐pentane (C_5_H_12_) were distilled under nitrogen over sodium. [D_6_]Benzene were dried over molecular sieves (4 Å). Tin dichloride was dried by vigorous stirring with acetic anhydride, while copper(I) chloride by co‐evaporation with toluene and drying under vacuum. Other chemicals were commercially available and used as received. For elemental analyses a LECO TruSpec CHN elementary analyzer, was utilized.

**NMR Spectroscopy**: Solution NMR spectra were recorded on Bruker AMX‐300, DRX‐400 and DRX‐500 spectrometers. Spectra were referenced to external SiMe_4_ (*δ*: 0 ppm) using the residual proton solvent peaks as internal standards (^1^H NMR experiments), or the characteristic resonances of the solvent nuclei (^13^C NMR experiments), whereas ^31^P was referenced to H_3_PO_4_. Spectral assignments were made by routine one‐ and two‐dimensional NMR experiments where appropriate.^103^Rh NMR was acquired at 15.9 MHz using an observe 5 mm triple resonance broadband probe (broadband inner coil and doubly tuned ^1^H/^31^P outer coil) with 90° pulses of 37.5 μs and 30.0 μs for ^103^Rh and ^31^P, respectively. ^103^Rh chemical shifts, *δ*, are given in ppm relative to *Ξ*=3.186447^[87]^ (reference compound Rh(acac)_3_, where acac stands for [CH_3_COCHCOCH_3_]^−^) and derived indirectly from the ^31^P‐^103^Rh HMQC experiments by four pulse ^31^P−^103^Rh HMQC experiments with ^1^H decoupling during acquisition. Note that despite the fact that IUPAC recommends the use of Rh(acac)_3_ as the reference, the alternative *Xi* value *Ξ*=3.160000 for Rh metal has been commonly employed in the literature. The experiments were optimized using the ^1^ *J*_RhP_ values obtained from the corresponding ^31^P{^1^H} spectra. The transmitter frequency offset and the spectral width were varied to ensure that no signals were folded. 2D data were zero filled and processed with exponential line broadening of 10 Hz in the direct F2 dimension, and unshifted sine‐bell window function in the indirect F1 dimension.

**General synthesis of rhodium MOLPs. Method A**: A solid mixture of **1** (30 mg, 0.077 mmol) and the corresponding Lewis acid (0.077 mmol: GeCl_2_⋅dioxane, 36 mg; SnCl_2_, 15 mg; CuCl, 7.6 mg; Zn(C_6_F_5_)_2_, 30 mg) is placed in a Schlenk flask and dissolved in toluene (4 mL; for Zn(C_6_F_5_)_2_) or bromobenzene (4 mL; for GeCl_2_, SnCl_2_ and CuCl) under argon atmosphere. The solution is stirred for one hour at 25 °C and pentane (10 mL) is subsequently added. The resulting solid is filtrated, dried under vacuum and washed with pentane to provide the resultant MOLPs as orange to brown solids in moderate to good yields (vide infra). **Method B**. A toluene (4 mL) solution of **1** (30 mg, 0.077 mmol) placed in a Schlenk flask was charged with a solution of the corresponding Lewis acid (ZnMe_2_, AlMe_3_ or MeMgBr, 1 m in toluene or Et_2_O, 77 μL, 0.077 mmol) and stirred for one hour at 25 °C. Then pentane (10 mL) is added and the resulting solid filtrated, dried under reduced pressure and washed with pentane to provide the resultant MOLPs as orange to brown solids in moderate to good yields (vide infra). In the case of ZnMe_2_ drying of the MOLP is carried out by a flow of argon, since under reduced pressure ZnMe_2_ is readily eliminated. Single crystals of compounds **1⋅GeCl_2_**, **1⋅SnCl_2_**, **1⋅AlMe_3_**, **1⋅ZnMe_2_**, **1⋅Zn(C_6_F_5_)_2_** and **3** were grown from slow diffusion of pentane into their benzene or bromobenzene solutions. The analogous procedures carried out in J. Young NMR tubes between **1** (14 mg, 0.036 mmol) and equimolar amounts of the corresponding Lewis acids lead in all cases to formation of the reported MOLPs in quantitative spectroscopic yields.

**Compound 1⋅GeCl_2_**: ^1^H NMR (400 MHz, C_6_D_5_Br, 298 K): *δ* 1.67 (s, 15 H, C_5_Me_5_), 1.55 ppm (t, ^2^ *J*_HP_=4.5 Hz, 18 H, PMe_3_). ^13^C{^1^H} NMR (101 MHz, C_6_D_5_Br, 298 K): *δ* 102.7 (s, *C_5_*Me_5_), 18.9 (t, ^1^ *J*_CP_=16 Hz, PMe_3_), 9.9 ppm (s, C_5_ *Me_5_*). ^31^P{^1^H} NMR (162 MHz, C_6_D_5_Br, 298 K): *δ*−7.0 ppm (d, ^1^ *J*_PRh_=171 Hz). ^103^Rh{^1^H} NMR (15.94 MHz, C_6_D_5_Br, 298 K): *δ*−8756 ppm. Anal. Calcd. for C_16_H_33_GeCl_2_P_2_Rh: C, 36.0; H, 6.2. Found: C, 36.3; H, 6.1. Yield: 31 mg, 76 %.

**Compound 1⋅SnCl_2_**: ^1^H NMR (400 MHz, C_6_D_5_Br, 298 K): *δ* 1.67 (s, 15 H, C_5_Me_5_), 1.56 ppm (t, ^2^ *J*_HP_=4.1 Hz, 18 H, PMe_3_). ^13^C{^1^H} NMR (101 MHz, C_6_D_5_Br, 298 K): *δ* 101.7 (s, *C_5_*Me_5_), 19.5 (t, ^1^ *J*_CP_=16 Hz, PMe_3_), 10.0 ppm (s, C_5_ *Me_5_*). ^31^P{^1^H} NMR (162 MHz, C_6_D_5_Br, 298 K): δ−8.5 ppm (d, ^1^ *J*_PRh_=169 Hz). ^119^Sn{^1^H} NMR (149 MHz, C_6_D_5_Br, 298 K): *δ* 810.7 ppm (br s). ^103^Rh{^1^H} NMR (15.94 MHz, C_6_D_5_Br, 298 K): *δ*−8836 ppm. Anal. Calcd. for C_16_H_33_Cl_2_P_2_RhSn: C, 33.1; H, 5.7. Found: C, 33.4; H, 6.1. Yield: 29 mg, 69 %.

**Compound 1⋅CuCl**: ^1^H NMR (400 MHz, C_6_D_5_Br, 298 K): *δ* 1.66 (s, 15 H, C_5_Me_5_), 1.48 ppm (br s, 18 H, PMe_3_). ^13^C{^1^H} NMR (101 MHz, C_6_D_5_Br, 298 K): *δ* 103.0 (s, *C_5_*Me_5_), 18.4 (t, ^1^ *J*_CP_=16 Hz, PMe_3_), 10.3 ppm (s, CH_3_). ^31^P{^1^H} NMR (162 MHz, C_6_D_5_Br, 298 K): *δ*−3.0 ppm (d, ^1^ *J*_PRh_=144 Hz). ^103^Rh{^1^H} NMR (15.94 MHz, C_6_D_5_Br, 298 K): *δ*−8540 ppm. Anal. Calcd. for C_16_H_33_CuClP_2_Rh: C, 39.3; H, 6.8. Found: C, 39.5; H, 6.9. Yield: 23 mg, 66 %.

**Compound 1⋅AlMe_3_**: ^1^H NMR (400 MHz, C_6_D_6_, 298 K): *δ* 1.67 (s, 15 H, CH_3_), 1.10 (t, ^2^ *J*_HP_=4.0 Hz, 18 H, PMe_3_), −0.06 ppm (s, 9 H, Al(CH_3_)_3_). ^13^C{^1^H} NMR (101 MHz, C_6_D_6_, 298 K): *δ* 100.9 (s, *C_5_*Me_5_), 21.0 (t, ^2^ *J*_CP_=15 Hz, PMe_3_), 11.3 (s, C_5_ *Me_5_*), 1.0 ppm (s, Al(CH_3_)_3_). ^31^P{^1^H} NMR (162 MHz, C_6_D_6_, 298 K): *δ*−6.9 ppm (d, ^1^ *J*_PRh_=181 Hz). ^103^Rh{^1^H} NMR (15.94 MHz, C_6_D_6_, 298 K): *δ*−9272 ppm. Anal. Calcd. for C_19_H_42_AlP_2_Rh: C, 49.3; H, 9.2. Found: C, 49.4; H, 9.3. Yield: 28 mg, 83 %.

**Compound 3**: ^1^H NMR (400 MHz, C_6_D_6_, 25 °C) *δ*: 1.87 (s, 15 H, C_5_Me_5_), 1.38 ppm (vt, 18 H, ^2^ *J*_HP_=6.4 Hz, PMe_3_). Signal due to CH_3_Mg could not be unambiguously identified. ^13^C{^1^H} NMR (101 MHz, C_6_D_6_, 25 °C) *δ*: 99.3 (*C*_5_Me_5_), 21.9 (vt, ^1^ *J*_CP_=12 Hz, PMe_3_), 11.3 (C_5_ *Me_5_*), 3.1 ppm (CH_3_Mg). ^31^P{^1^H} NMR (162 MHz, C_6_D_6_, 25 °C) *δ*: −10.2 ppm (d, ^1^ *J*_PRh_=172 Hz). ^103^Rh{^1^H} NMR (15.9 MHz, C_6_D_6_, 25 °C) *δ*: −9404 ppm. Anal. Calcd. for C_16.25_H_33.75_Br_1.75_MgP_2_Rh: C, 34.1; H, 5.9. Found: C, 34.4; H, 6.4. Yield: 28 mg, 83 %.

**Compound 1⋅Zn(C_6_F_5_)_2_**: ^1^H NMR (400 MHz, C_6_D_6_, 25 °C) *δ*: 1.59 (s, 15 H, C_5_Me_5_), 1.06 ppm (vt, 18 H, ^2^ *J*_HP_=3.7 Hz, PMe_3_). ^13^C{^1^H} NMR (101 MHz, C_6_D_6_, 25 °C) *δ*: 140–135.0 (br, C_6_F_5_), 98.8 (*C*_5_Me_5_), 20.6 (vt, ^1^ *J*_CP_=16 Hz, PMe_3_), 10.2 (C_5_ *Me_5_*). ^13^C{^1^H} NMR (101 MHz, [D_8_]toluene, −10 °C) *δ*: 144.6 (dd, C_6_F_5_), 135.3 (br t, C_6_F_5_), 128.9 (t, C_6_F_5_), 97.0 (s, *C*_5_Me_5_), 91.5 (C_ipso_(C_6_F_5_)), 18.3 (vt, ^1^ *J*_CP_=16 Hz, PMe_3_), 8.2 ppm (C_5_ *Me_5_*). ^13^C signals due to C_6_F_5_ fragments partly resolved at −10 °C, but a fully unambiguous assignment could not be made; see spectra below. ^19^F{^1^H} NMR (376 MHz, C_6_D_6_, 25 °C) *δ*: −161.3 (t, ^1^ *J*_CF_=21 Hz, *m*‐C_6_F_5_), −158.1 (t, ^1^ *J*_CF_=20 Hz, 2F, *p*‐C_6_F_5_), −115.0 ppm (d, ^1^ *J*_CF_=23 Hz, 4F, *o*‐C_6_F_5_). ^31^P{^1^H} NMR (162 MHz, C_6_D_6_, 25 °C) *δ*: −7.2 ppm (d, ^1^ *J*_PRh_=167 Hz). ^103^Rh{^1^H} NMR (15.9 MHz, C_6_D_6_, 25 °C) *δ*: −9355 ppm. Anal. Calcd. for C_28_H_33_F_10_P_2_Rh: C, 42.6; H, 4.2. Found: C, 42.2; H, 4.6. Yield: 28 mg, 83 %.

**Compound 1⋅ZnMe_2_**: ^1^H NMR (400 MHz, C_6_D_6_, 25 °C) *δ*: 1.76 (s, 15 H, C_5_Me_5_), 1.09 (br vt, 18 H, ^2^ *J*_HP_=3.7 Hz, PMe_3_), −0.41 ppm (s, 6 H, ZnMe_2_). ^13^C{^1^H} NMR (101 MHz, C_6_D_6_, 25 °C) *δ*: 97.4 (s, *C*_5_Me_5_), 21.8 (vt, ^1^ *J*_CP_=14 Hz, PMe_3_), 10.9 (s, C_5_ *Me_5_*), −5.1 ppm (ZnMe_2_). ^31^P{^1^H} NMR (162 MHz, C_6_D_6_, 25 °C) *δ*: −6.9 ppm (d, ^1^ *J*_PRh_=192 Hz). ^103^Rh{^1^H} NMR (15.9 MHz, C_6_D_6_, 25 °C) *δ*: −9212 ppm. Anal. Calcd. for C_18_H_39_P_2_RhZn: C, 44.5; H, 8.1. Found: C, 45.0; H, 7.6. Yield: 28 mg, 83 %.

**X‐Ray structural characterization of new compounds**: CCDC 1996856, 1996860, 1996858, 1996859, 1996857 and 1996855 for **1⋅GeCl_2_**, **1⋅SnCl_2_**, **1⋅AlMe_3_**, **1⋅ZnMe_2_**, **1⋅Zn(C_6_F_5_)_2_** and **3**, respectively, contain the supplementary crystallographic data for this paper. These data are provided free of charge by The Cambridge Crystallographic Data Centre.

## Conflict of interest

The authors declare no conflict of interest.

## Biographical Information

*Jesús Campos obtained his PhD (2012) in the group of Prof. Carmona (Sevilla) working on fundamental organometallic chemistry, including a visiting stay in the group of Prof. Brookhart (UNC). His postdoctoral studies took place at the Universities of Yale and Oxford within the groups of Profs. Crabtree and Aldridge, respectively. In 2016 he moved back to the University of Sevilla as a Marie Curie fellow and one year later he was appointed Tenured Scientist of the Spanish National Research Council (CSIC). Since then his group has focused on the use of organometallic complexes as a platform to investigate new modes of chemical cooperation under the umbrella of an ERC Starting Grant project*.

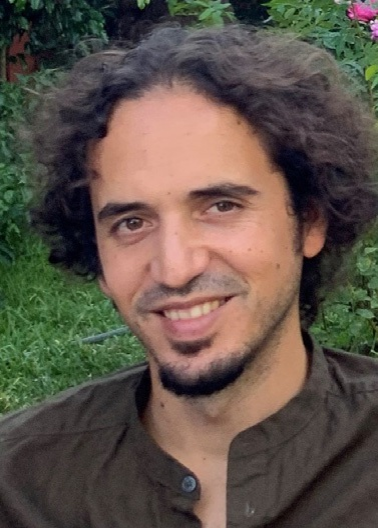

## Supporting information

As a service to our authors and readers, this journal provides supporting information supplied by the authors. Such materials are peer reviewed and may be re‐organized for online delivery, but are not copy‐edited or typeset. Technical support issues arising from supporting information (other than missing files) should be addressed to the authors.

Supplementary
