# Supplementary material for: Metal‐only Lewis Pairs of Rhodium with s, p and d‐Block Metals
Source: Chemistry. 2020 Nov 9;26(70):16833–45. doi: 10.1002/chem.202003167 (PMC7756578; doi:10.1002/chem.202003167)
Supplement: Supplementary file 1 — Supplementary [file CHEM-26-16833-s001.pdf]

Chemistry–A European Journal

Supporting Information

Metal-only Lewis Pairs of Rhodium with *s*, *p* and *d*-Block Metals

Sonia Bajo+, Macarena G. Alférez+, María M. Alcaide, Joaquín López-Serrano,* and Jesús Campos*[a]

SUPPORTING INFORMATION

1. Synthesis and characterization of rhodium compounds. S2
2. X-Ray structural characterization of new compounds S3
3. NMR spectra of new compounds S6
4. Additional computational details.. S23
5. References S30

1. Synthesis and characterization of rhodium compounds.

Compound 1 [(η^5-C_5Me_5)Rh(PMe_3)$_2$]. We used a slight modification of the procedure previously reported by Werner.[1] A sodium amalgam was prepared by dissolving Na metal (172 mg, 7.5 mmol) onto mercury (4.5 mL) under argon atmosphere. Diethyl ether (20 mL), PMe_3 (6.8 mL, 6.66 mmol) and a toluene (5 mL) solution of [(η^5-C_5Me_5)$RhCl_2$]$_2$[2] (927 mg, 1.5 mmol) were subsequently added stepwise. The mixture was stirred for 8 hours after which it was filtrated and extracted with pentane (20 mL). The red solution was concentrated to *ca*. 5 mL and stored at -78 ºC. Rhombic brown crystals of **1** were obtained after 5 days (750 mg, 60%). ^{1}H NMR (300 MHz, C_6D_6, 25 ºC) δ: 2.17 (s, 15 H, C_5Me_5), 1.3 (vt, 18 H, PMe_3). ^{31}P {^{1}H} NMR (121 MHz, C_6D_6, 25 ºC) δ: -6.6 (d, $^1J_{PRh}$ = 216 Hz). ^{103}Rh {^{1}H} NMR (15.94 MHz, C_6D_6, 25 ºC) δ: -9165.

Compound [(η^5-C_5Me_5)RhH(PMe_3)$_2$]PF_6. The rhodium hydride was best prepared by adding NH_4PF_6 (50 mg, 0.12 mmol) over a THF (5 mL) solution of **1** (21 mg, 0.12 mmol). The solution was stirred during 30 minutes, time after which addition of pentane (20 mL) caused precipitation of **2**. The recorded spectroscopic data matched those previously reported by Werner[1]. ^{1}H NMR (400 MHz, THF, 25 ºC) δ: 1.99 (s, 15 H, C_5Me_5), 1.62 (br vt, 18 H, PMe_3, $^2J_{HP}$ = 5.1Hz), -13.35 (td, RhH, $^2J_{HP}$= 23.4 Hz, $^1J_{HRh}$ = 34.9 Hz). ^{13}C {^{1}H} NMR (100 MHz, THF, 25 ºC,) δ: 11.0 (C_5*Me*$_5$), 20.7 (vt, $^1J_{CP}$ = 16 Hz, PMe_3), 103.0 (*C*$_5$Me$_5$). ^{31}P {^{1}H} NMR (162 MHz, THF, 25 ºC) δ: -1.4 (dd, $^1J_{PRh}$ = 137 Hz, $^2J_{PP}$ = 14 Hz).

Addition of MBAr$_F$ (M = Li, Na) to 1. A J. Young NMR tube was charged with **1** (7 mg, 0.018 mmol), MBAr^F (M = Li, Na; 16 mg, 0.018 mmol) and deuterated bromobenzene (0.5 mL). Complete consumption of **1** towards the postulated **[A·M]** compounds was instantaneous (100% NMR yield). Reactions at higher concentrations proved inconvenient due to reduced solubility. Selected spectroscopic data: **[A·Na].** ^{1}H NMR (400 MHz, C_6D_5Br, 25 ºC): δ 8.40 (s, 8 H, *o*-Ar), 7.81 (s, 4 H, *p*-Ar), 1.66 (s, 15 H, C_5Me_5), 1.17 (br, 18 H, PMe_3). ^{13}C{^{1}H} NMR (101 MHz, C_6D_5Br, 25 ºC): δ 162.2 (br, *ipso*-Ar), 135.2 (*o*-Ar), 117.9 (*p*-Ar), 101.8 (s, *C*$_5$Me$_5$), 20.3 (t, $^1J_{CP}$ = 17 Hz, PMe_3), 10.6 (s, C_5*Me*$_5$). ^{31}P{^{1}H} NMR (202 MHz, C_6D_5Br, 25 ºC): δ -3.1 (d, $^1J_{PRh}$ = 138). ^{103}Rh{^{1}H} NMR (15.94 MHz, C_6D_5Br, 25 ºC): δ -9262. **[A·Li].** ^{1}H NMR (400 MHz, C_6D_5Br, 25 ºC): δ 8.96 (s, 8 H, *o*-Ar), 8.86 (s, 4 H, *p*-Ar), 1.61 (s, 15 H, C_5Me_5), 1.11 (br, 18 H, PMe_3). ^{31}P{^{1}H} NMR (202 MHz, C_6D_5Br, 25 ºC): δ -3.0 (d, $^1J_{PRh}$ = 130).^{103}Rh{^{1}H} NMR (15.94 MHz, C_6D_5Br, 298 K): δ -9261.

2. X-Ray structural characterization of new compounds

Crystallographic details. Low-temperature diffraction data were collected on a Bruker D8 Quest APEX-III single crystal diffractometer with a Photon III detector and a IμS 3.0 microfocus X-ray source (**1·GeCl$_2$**, **1·SnCl$_2$**, **1·AlMe$_3$**, **1·ZnMe$_2$**, **1·Zn(C$_6$F$_5$)$_2$**, **3**) at the Instituto de Investigaciones Químicas, Sevilla. Data were collected by means of ω and φ scans using monochromatic radiation λ(Mo Kα1) = 0.71073 Å. The diffraction images collected were processed and scaled using APEX-III or APEX-II software. The structures were solved with SHELXT and was refined against F^2 on all data by full-matrix least squares with SHELXL.[3] All non-hydrogen atoms were refined anisotropically. Hydrogen atoms were included in the model at geometrically calculated positions and refined using a riding model, unless otherwise noted. The isotropic displacement parameters of all hydrogen atoms were fixed to 1.2 times the U value of the atoms to which they are linked (1.5 times for methyl groups). **1·GeCl$_2$**, **1·ZnMe$_2$** and **1·AlMe$_3$** were refined as inversion twins. For the latter anisotropic displacement parameters of the terminal methyl group of PMe$_3$ ligands were restrained to be similar to their phosphorus nuclei by using SIMU and DELU commands. Structure **3** was refined as a twinned structure with 68:32 components and present substitutional disorder between a bromide and a methyl group at the terminal Mg-X fragment (Br:Me, 74:26).

The full numbering scheme of all the reported structures can be found in the full details of the X-ray structure determination (CIF), which is included as Supporting Information.

Table S1. Crystal data and structure refinement for compounds **1·GeCl$_2$**, **1·SnCl$_2$** and **1·AlMe$_3$**.

	**1·GeCl$_2$**	**1·SnCl$_2$**	**1·AlMe$_3$**
formula	$C_{16}H_{33}Cl_2GeP_2Rh$	$C_{16}H_{33}Cl_2P_2RhSn$	$C_{19}H_{42}AlP_2Rh$
fw	533.76	579.86	462.35
cryst.size, mm	0.18 × 0.14 × 0.10	0.12 × 0.09 × 0.07	0.20 × 0.10 × 0.09
crystal system	Monoclinic	Monoclinic	Orthorhombic
space group	$P2_1$	$P2_1/n$	$Pna2_1$
a, Å	8.7747 (5)	8.8303 (3)	17.2718 (16)
b, Å	29.4171 (19)	44.1554 (12)	9.1353 (8)
c, Å	16.9592 (8)	17.1551 (5)	14.9420 (11)
α, deg	90	90	90
β, deg	94.002 (2)	92.967 (1)	90
γ, deg	90	90	90
V, Å^3	4366.9 (4)	6679.9 (3)	2357.6 (3)
T, K	193	193	193
Z	8	12	4
ρ_{calc}, g cm^{-3}	1.624	1.730	1.303
μ, mm^{-1} (MoKα)	2.52	2.24	0.90
F(000)	2160	3456	976
absorption corrections	multi-scan, 0.63-0.75	multi-scan, 0.59-0.75	multi-scan, 0.61-0.75
θ range, deg	2.3 – 29.1	2.2 – 29.6	2.4 – 25.7
no. of rflns measd	44756	116036	14836
R_{int}	0.047	0.034	0.056
no. of rflns unique	18847	18751	4420
no. of params / restraints	837 / 1	628 / 0	219 / 49
R_1 ($I > 2\sigma(I)$) [a]	0.037	0.035	0.057
R_1 (all data)	0.047	0.042	0.073
wR_2 ($I > 2\sigma(I)$)	0.070	0.077	0.140
wR_2 (all data)	0.073	0.079	0.152
Diff.Fourier.peaks min/max, eÅ^{-3}	-0.59 / 0.89	-1.50 / -1.07	-1.31 / 1.00
CCDC number	1996856	1996860	1996858

Table S2. Crystal data and structure refinement for compounds **1·ZnMe₂**, **1·Zn(C₆F₅)₂** and **3**.

	**1·ZnMe$_2$**	**1·Zn(C$_6$F$_5$)$_2$**	**3**
formula	$C_{18}H_{39}P_2RhZn$	$C_{31}H_{36}F_{10}P_2RhZn$	$C_{32.52}H_{67.55}Br_{3.48}Mg_2P_4Rh_2$
fw	485.71	828.82	1115.06
cryst.size, mm	0.22 × 0.20 × 0.12	0.22 × 0.20 × 0.16	0.12 × 0.08 × 0.04
crystal system	Monoclinic	Monoclinic	Monoclinic
space group	$P2_1$	$P2_1/n$	$P2_1/c$
a, Å	8.6079 (5)	10.6975 (5)	9.9631 (5)
b, Å	15.1035 (9)	19.1905 (9)	15.0180 (9)
c, Å	9.4286 (7)	16.6640 (9)	14.9008 (9)
α, deg	90	90	90
β, deg	112.528 (2)	101.283 (2)	101.406 (2)
γ, deg	90	90	90
V, Å^3	1132.27 (13)	3354.8 (3)	2185.5 (2)
T, K	193	193	193
Z	2	4	2
ρ_{calc}, g cm^{-3}	1.425	1.641	1.694
μ, mm^{-1} (MoKα)	1.93	1.38	4.13
F(000)	504	1668	1117
absorption corrections	multi-scan, 0.64-0.75	multi-scan, 0.61-0.75	multi-scan, 0.02-0.04
θ range, deg	2.3 – 25.7	2.2 – 30.6	2.0 – 25.1
no. of rflns measd	13448	162363	3862
R_{int}	0.052	0.045	-
no. of rflns unique	4302	10275	3862
no. of params / restraints	213 / 1	417 / 17	222 / 0
R_1 ($I > 2\sigma(I)$) [a]	0.031	0.032	0.056
R_1 (all data)	0.036	0.048	0.069
wR_2 ($I > 2\sigma(I)$)	0.058	0.081	0.144
wR_2 (all data)	0.060	0.099	0.150
Diff.Fourier.peaks min/max, eÅ^{-3}	-0.33 / 0.69	-0.70 / 0.77	-0.98 / 2.33
CCDC number	1996859	1996857	1996855

3. NMR spectra of new compounds

We report here multinuclear NMR spectra of all new compounds. In some cases those are the ones from freshly prepared samples prior to work-up, for which quantitative spectroscopic yields were typically observed. In particular, for compounds **1·GeCl$_2$**, **1·Zn(C$_6$F$_5$)** and **3**, using a freshly prepared solution permits to acquire spectra with a higher signal/noise ratio, since the isolated MOLPs exhibit reduce solubility. In the case of **1·ZnMe$_2$** work-up was problematic due to the volatility of ZnMe$_2$. For all others, the reported spectra are from isolated solids.

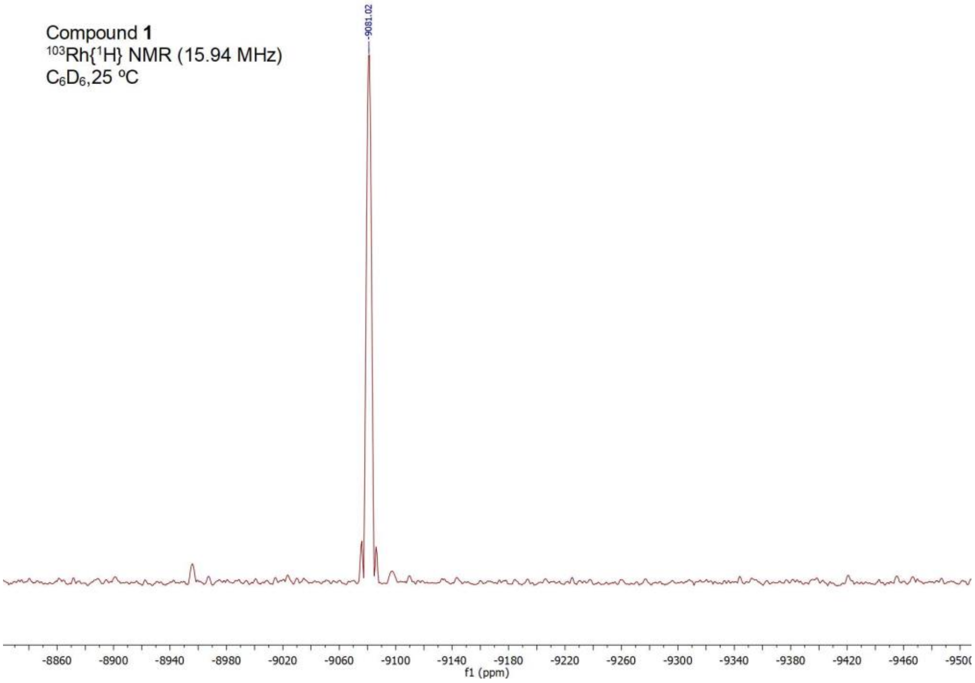

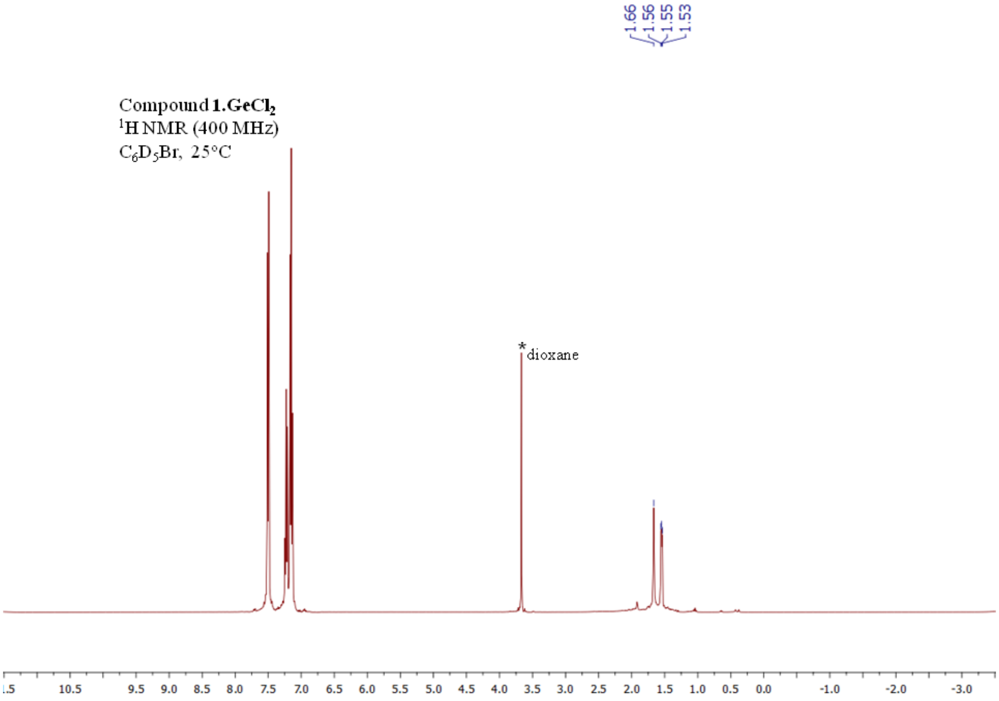

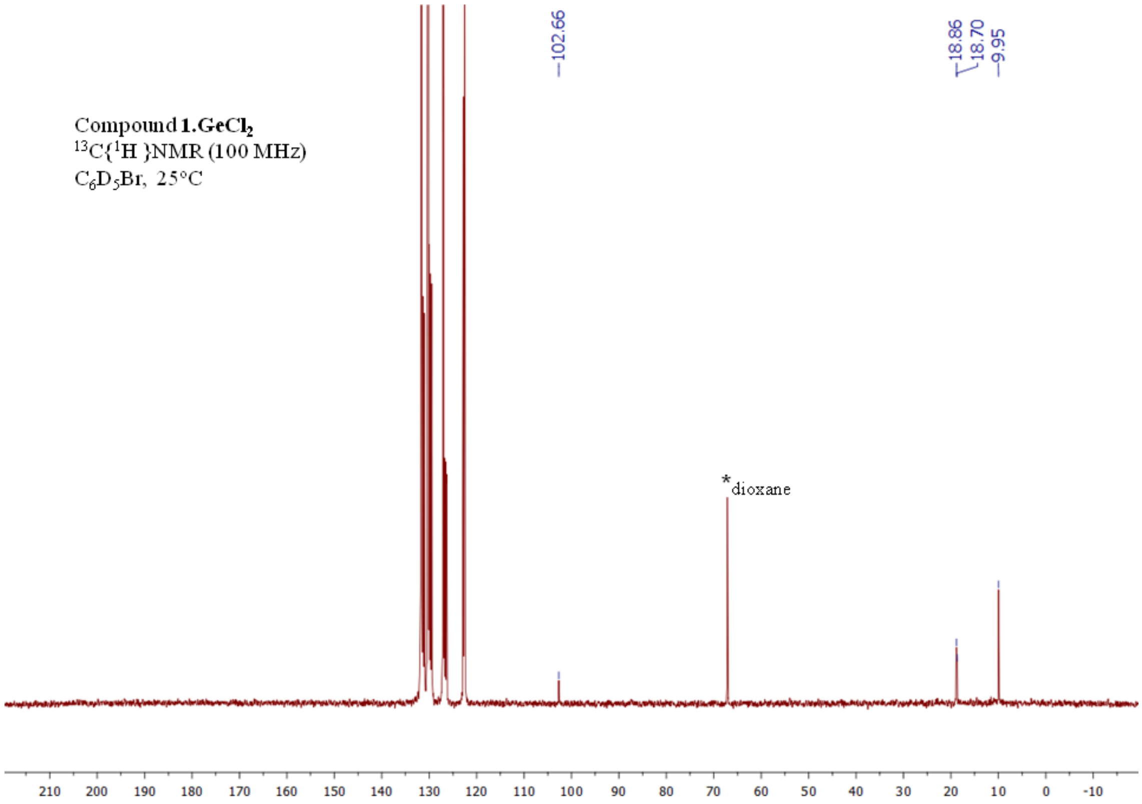
Compound 1.GeCl2
13C{1H }NMR (100 MHz)
C6D5Br, 25°C
102.66
18.86
18.70
9.95
*dioxane
210 200 190 180 170 160 150 140 130 120 110 100 90 80 70 60 50 40 30 20 10 0 -10

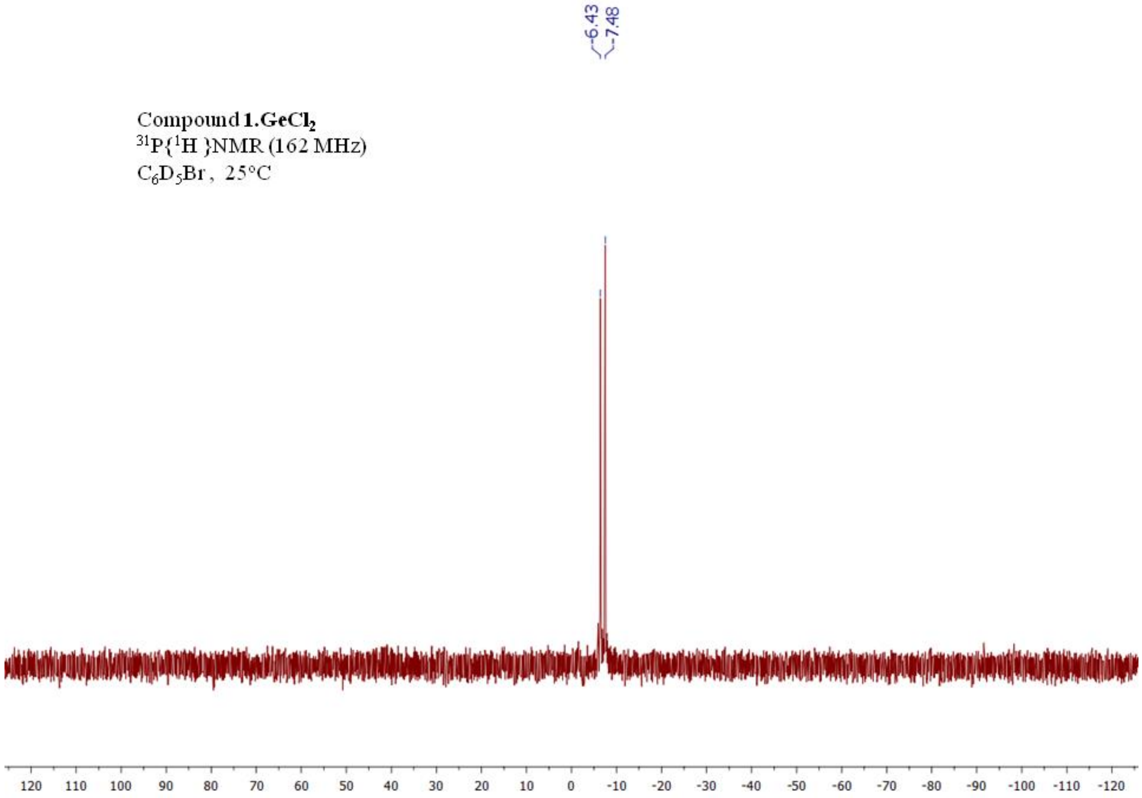
Compound 1.GeCl2
31P{1H }NMR (162 MHz)
C6D5Br, 25°C
-6.43
-7.48
120 110 100 90 80 70 60 50 40 30 20 10 0 -10 -20 -30 -40 -50 -60 -70 -80 -90 -100 -110 -120

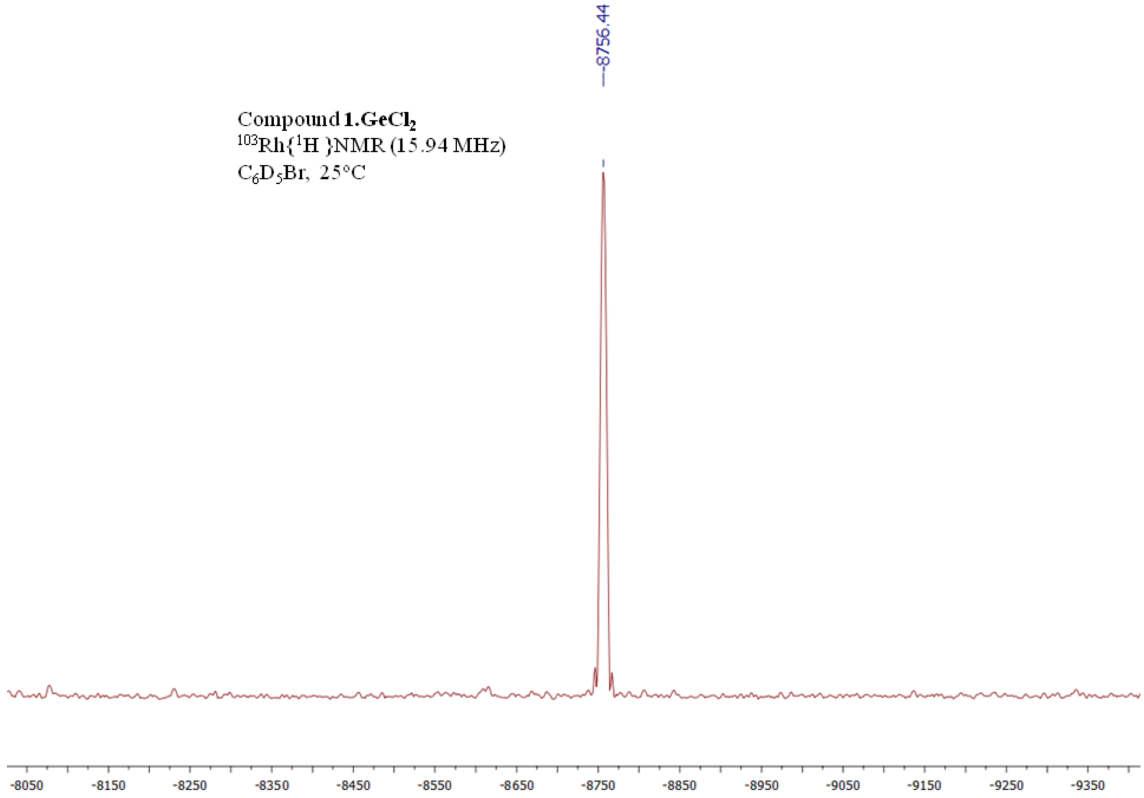
-8756.44
Compound **1.GeCl$_2$**
^{103}Rh{^{1}H }NMR (15.94 MHz)
C_6D_5Br, 25°C
-8050 -8150 -8250 -8350 -8450 -8550 -8650 -8750 -8850 -8950 -9050 -9150 -9250 -9350

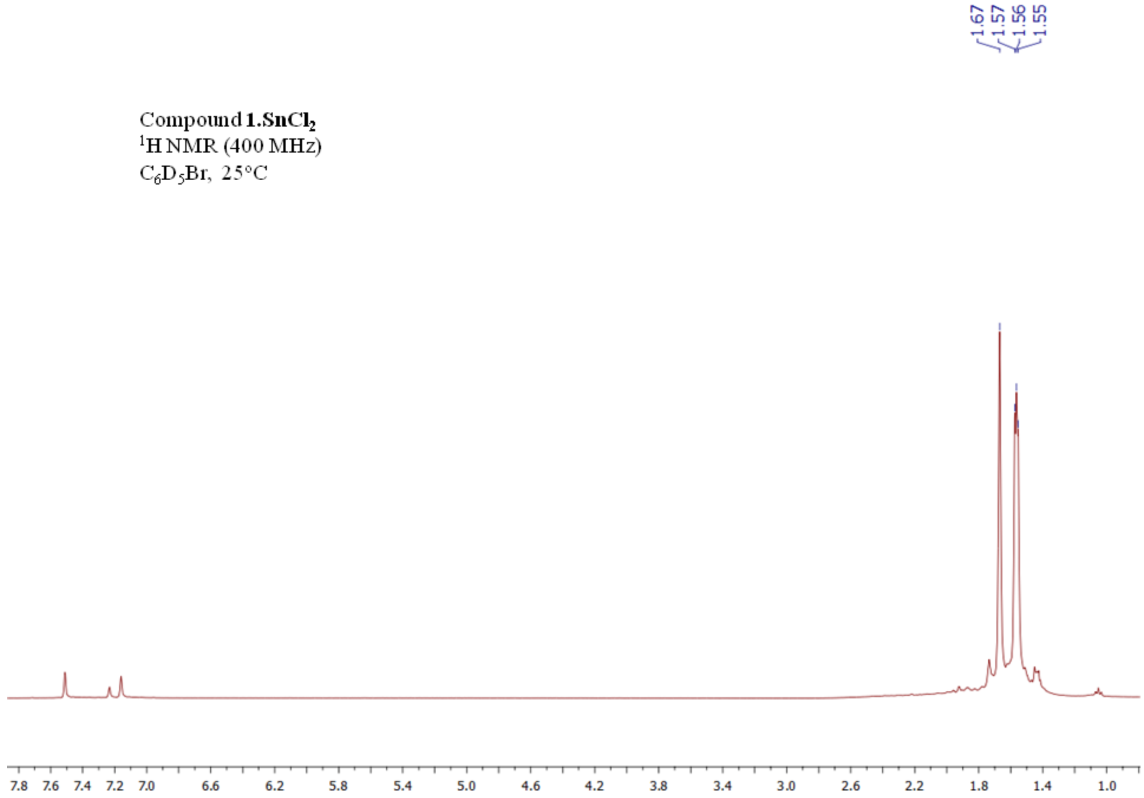
1.67
1.57
1.56
1.55
Compound **1.SnCl$_2$**
^{1}H NMR (400 MHz)
C_6D_5Br, 25°C
7.8 7.6 7.4 7.2 7.0 6.6 6.2 5.8 5.4 5.0 4.6 4.2 3.8 3.4 3.0 2.6 2.2 1.8 1.4 1.0

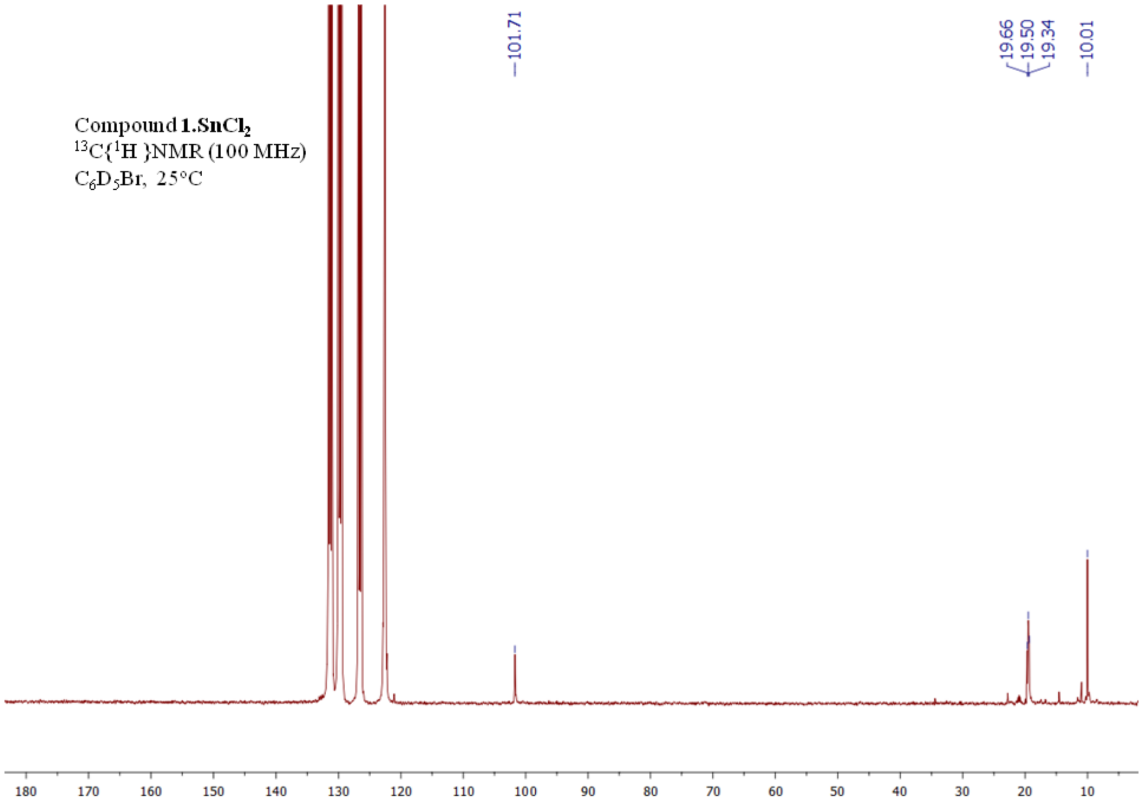

101.71
19.66
19.50
19.34
10.01
Compound 1.SnCl$_2$
$^{13}C\{^1H\}$NMR (100 MHz)
C_6D_5Br, 25°C
180 170 160 150 140 130 120 110 100 90 80 70 60 50 40 30 20 10

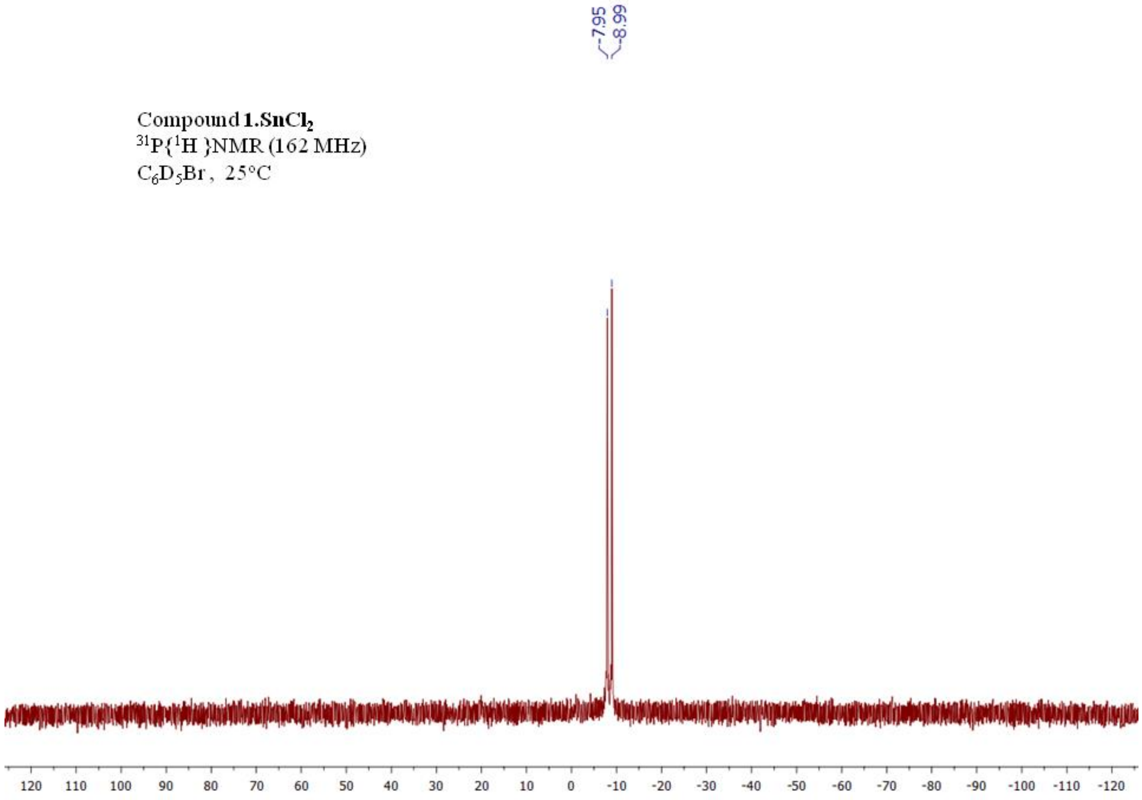

-7.95
-8.99
Compound 1.SnCl$_2$
$^{31}P\{^1H\}$NMR (162 MHz)
C_6D_5Br, 25°C
120 110 100 90 80 70 60 50 40 30 20 10 0 -10 -20 -30 -40 -50 -60 -70 -80 -90 -100 -110 -120

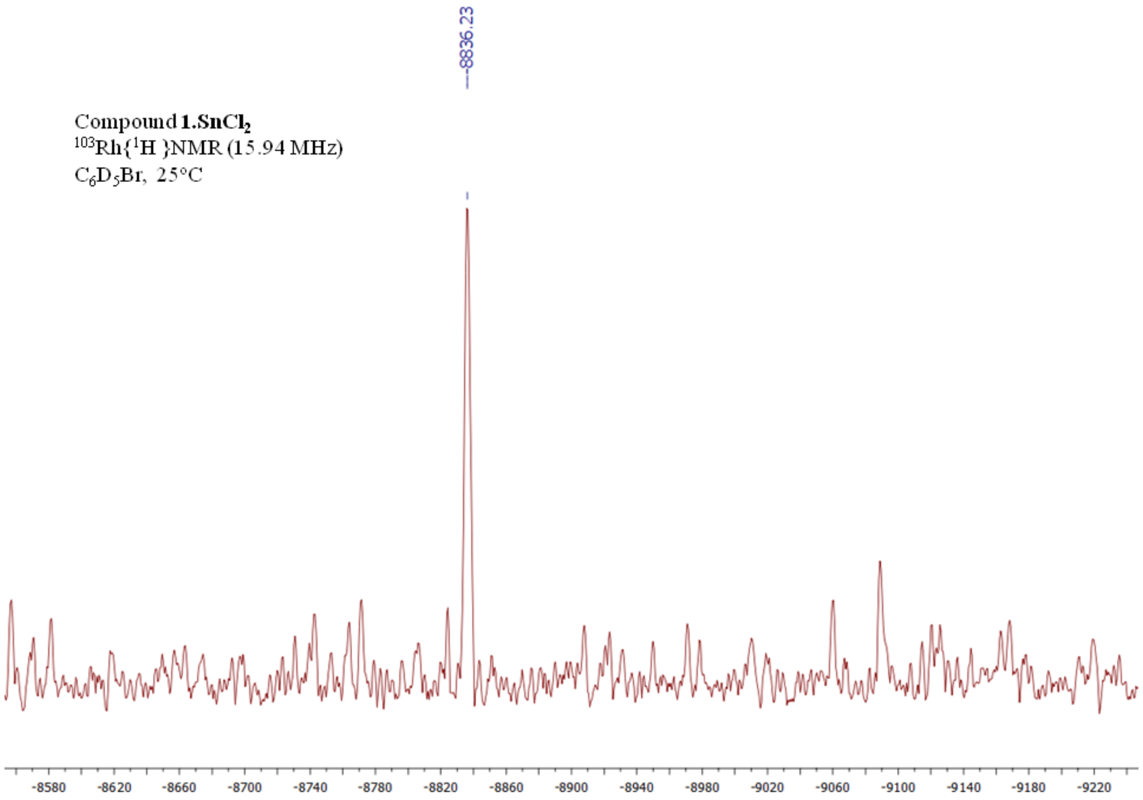
-8836.23
Compound 1.SnCl2
103Rh{1H }NMR (15.94 MHz)
C6D5Br, 25°C
-8580 -8620 -8660 -8700 -8740 -8780 -8820 -8860 -8900 -8940 -8980 -9020 -9060 -9100 -9140 -9180 -9220

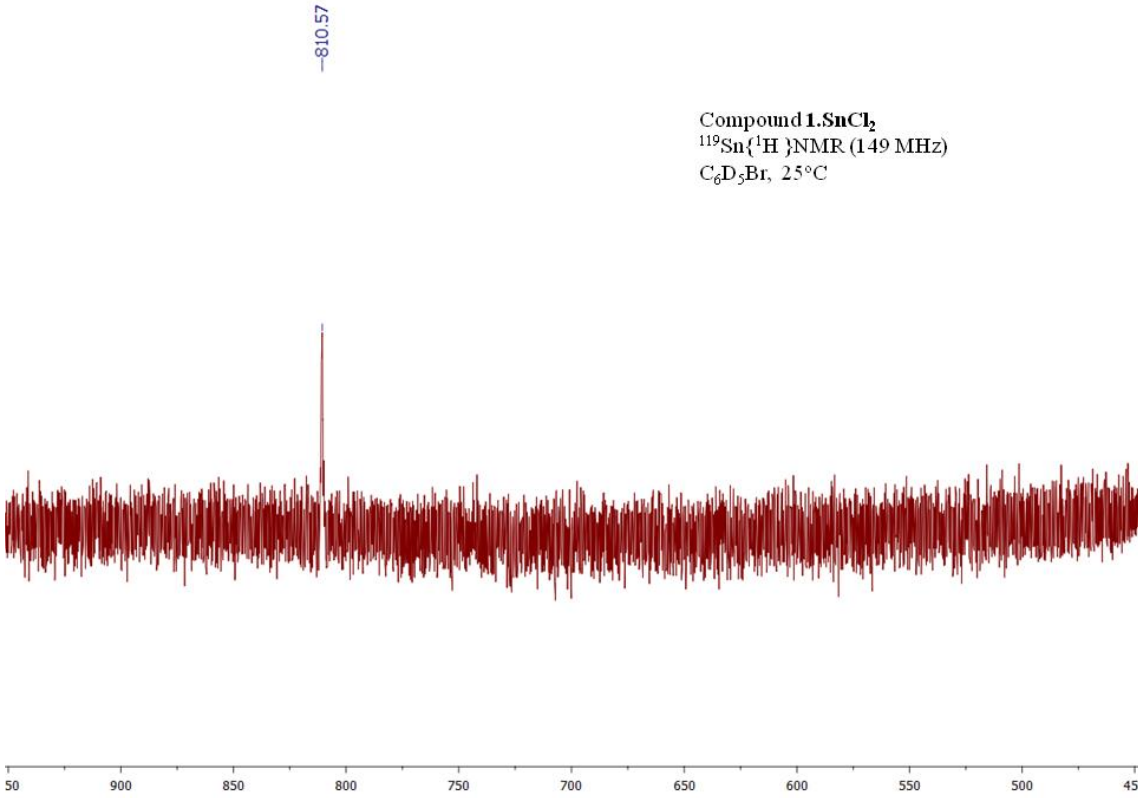
-810.57
Compound 1.SnCl2
119Sn{1H }NMR (149 MHz)
C6D5Br, 25°C
50 900 850 800 750 700 650 600 550 500 45

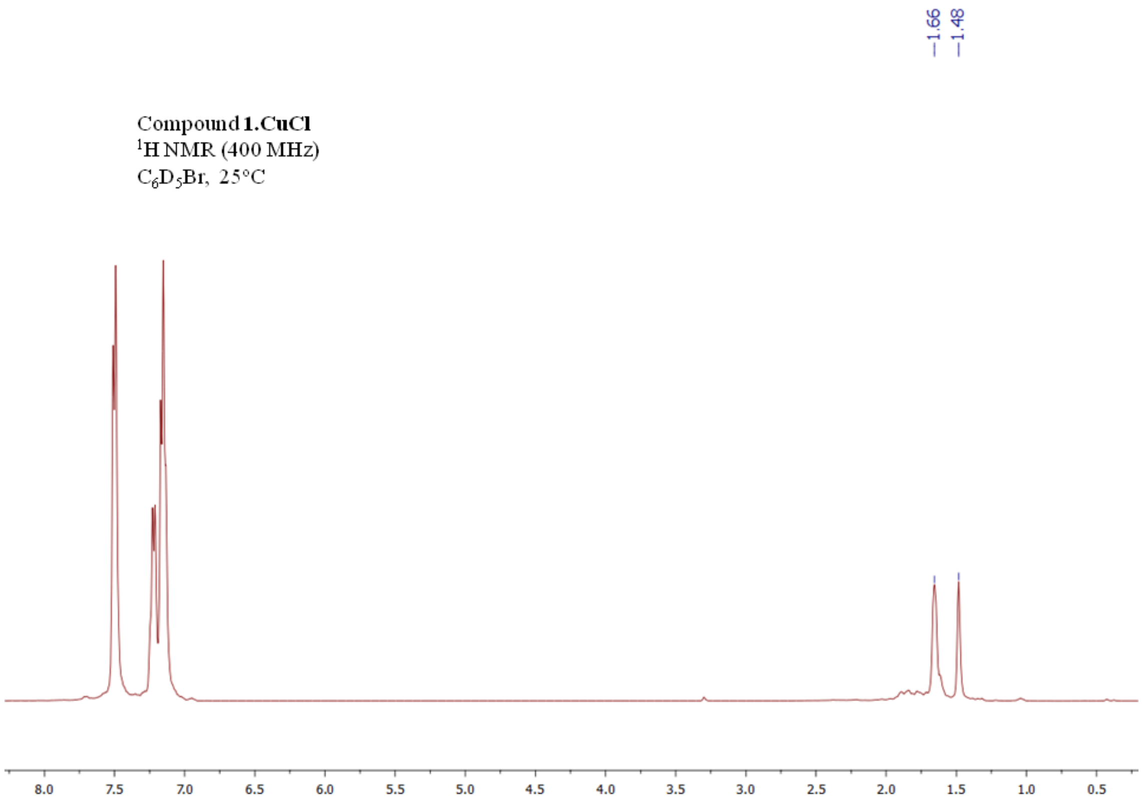
1.66
1.48
Compound 1.CuCl
1H NMR (400 MHz)
C6D5Br, 25°C
8.0 7.5 7.0 6.5 6.0 5.5 5.0 4.5 4.0 3.5 3.0 2.5 2.0 1.5 1.0 0.5

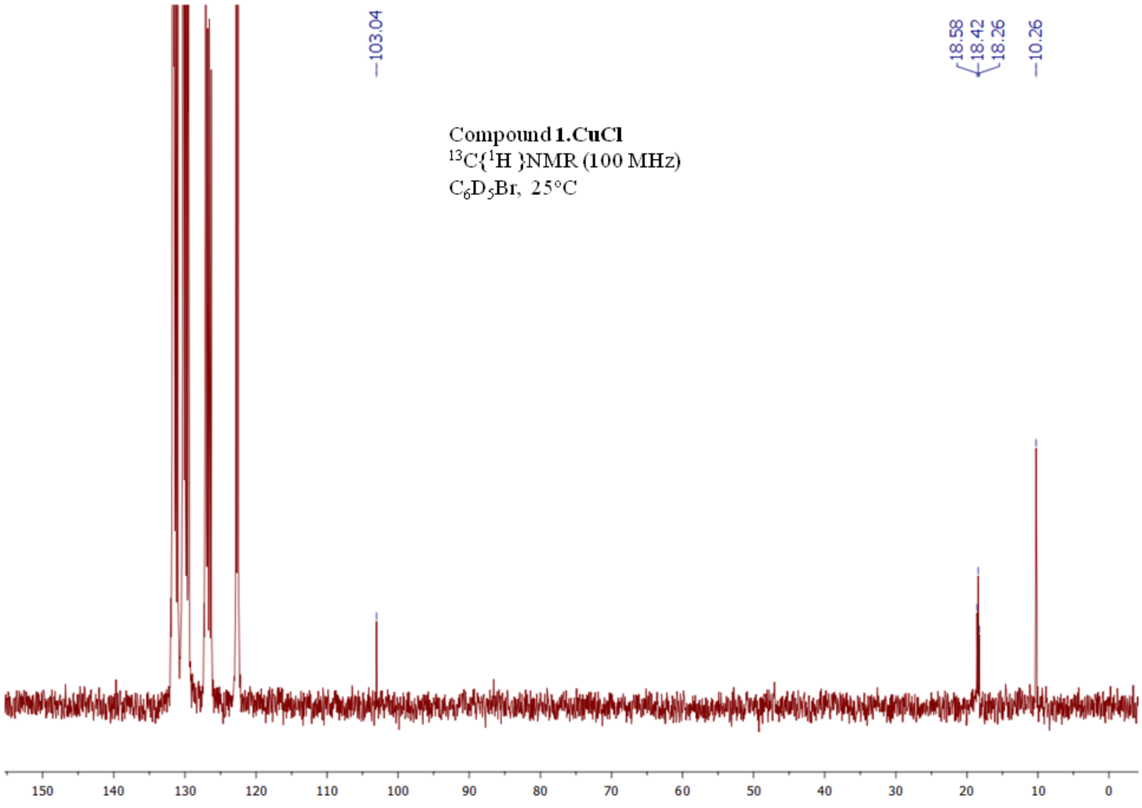
103.04
18.58
18.42
18.26
10.26
Compound 1.CuCl
13C{1H }NMR (100 MHz)
C6D5Br, 25°C
150 140 130 120 110 100 90 80 70 60 50 40 30 20 10 0

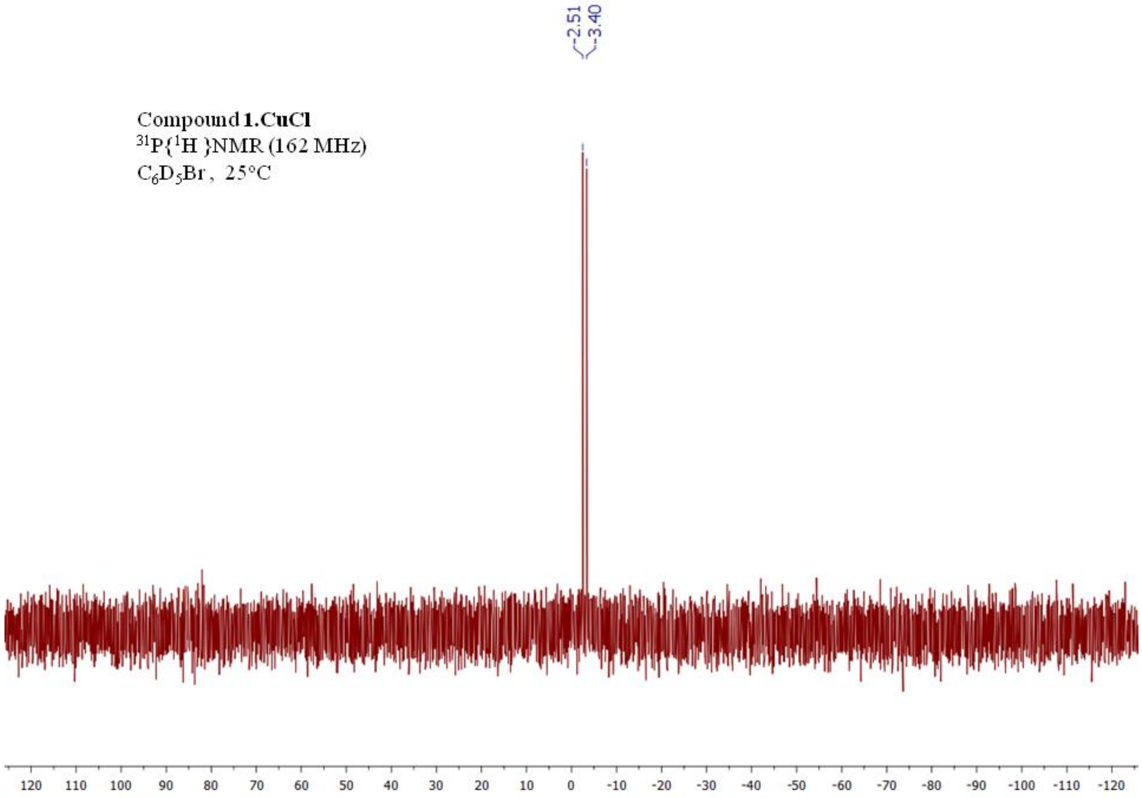
-2.51
-3.40
Compound 1.CuCl
$^{31}P\{^1H\}$NMR (162 MHz)
C_6D_5Br, 25°C
120 110 100 90 80 70 60 50 40 30 20 10 0 -10 -20 -30 -40 -50 -60 -70 -80 -90 -100 -110 -120

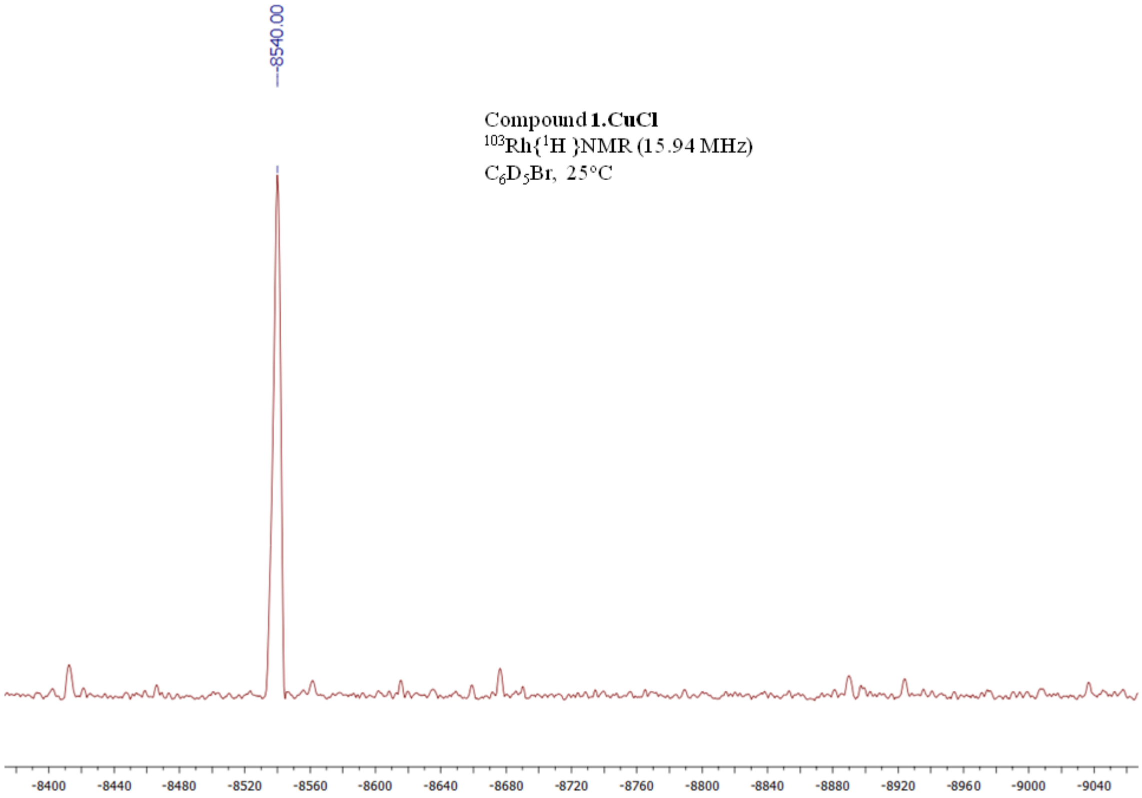
-8540.00
Compound 1.CuCl
$^{103}Rh\{^1H\}$NMR (15.94 MHz)
C_6D_5Br, 25°C
-8400 -8440 -8480 -8520 -8560 -8600 -8640 -8680 -8720 -8760 -8800 -8840 -8880 -8920 -8960 -9000 -9040

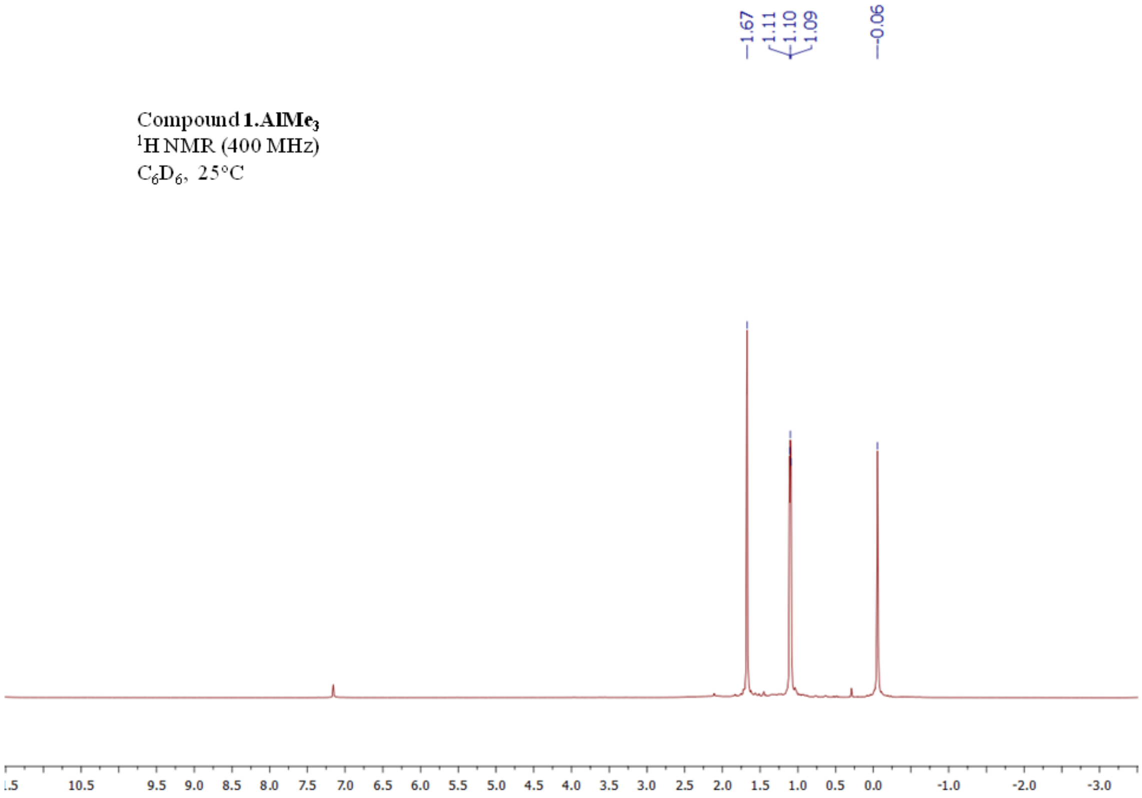

1.67
1.11
1.10
1.09
0.06
Compound **1.AlMe$_3$**
^{1}H NMR (400 MHz)
C_6D_6, 25°C
.5
10.5
9.5
9.0
8.5
8.0
7.5
7.0
6.5
6.0
5.5
5.0
4.5
4.0
3.5
3.0
2.5
2.0
1.5
1.0
0.5
0.0
-1.0
-2.0
-3.0

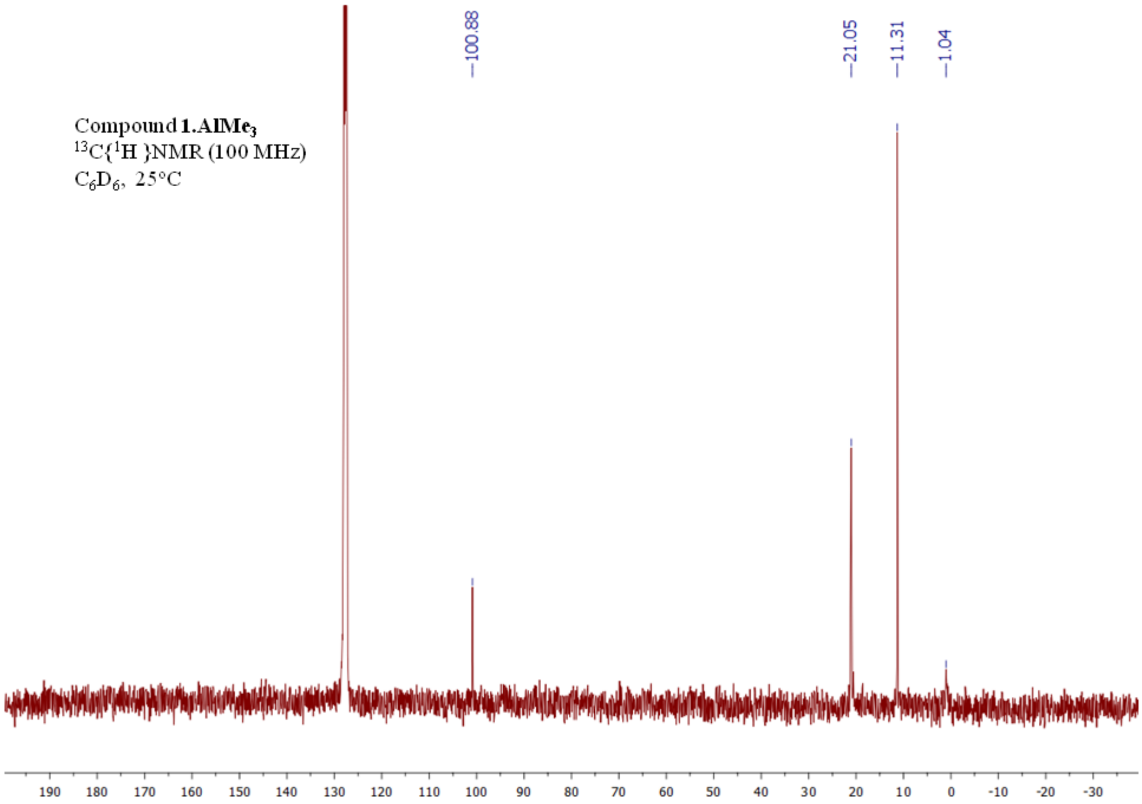

100.88
21.05
11.31
1.04
Compound **1.AlMe$_3$**
$^{13}C\{^1H\}$NMR (100 MHz)
C_6D_6, 25°C
190
180
170
160
150
140
130
120
110
100
90
80
70
60
50
40
30
20
10
0
-10
-20
-30

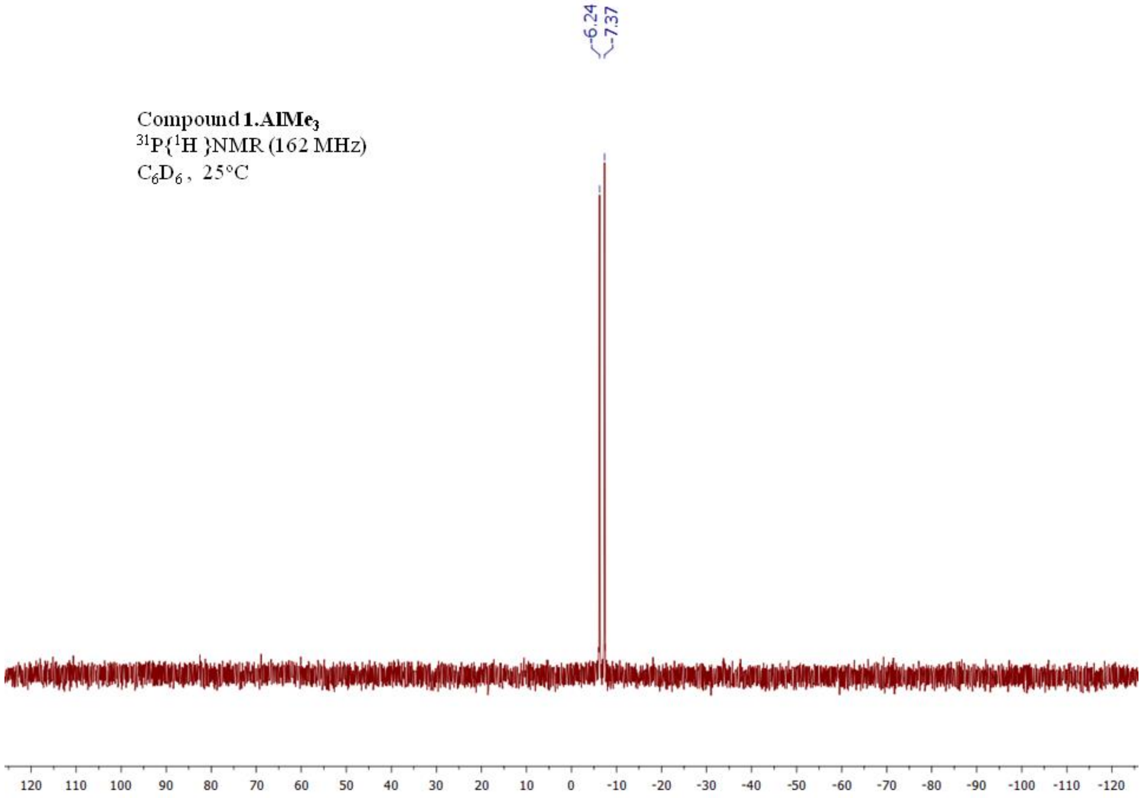
Compound 1.AlMe3
31P{1H }NMR (162 MHz)
C6D6 , 25°C
-6.24
-7.37
120 110 100 90 80 70 60 50 40 30 20 10 0 -10 -20 -30 -40 -50 -60 -70 -80 -90 -100 -110 -120

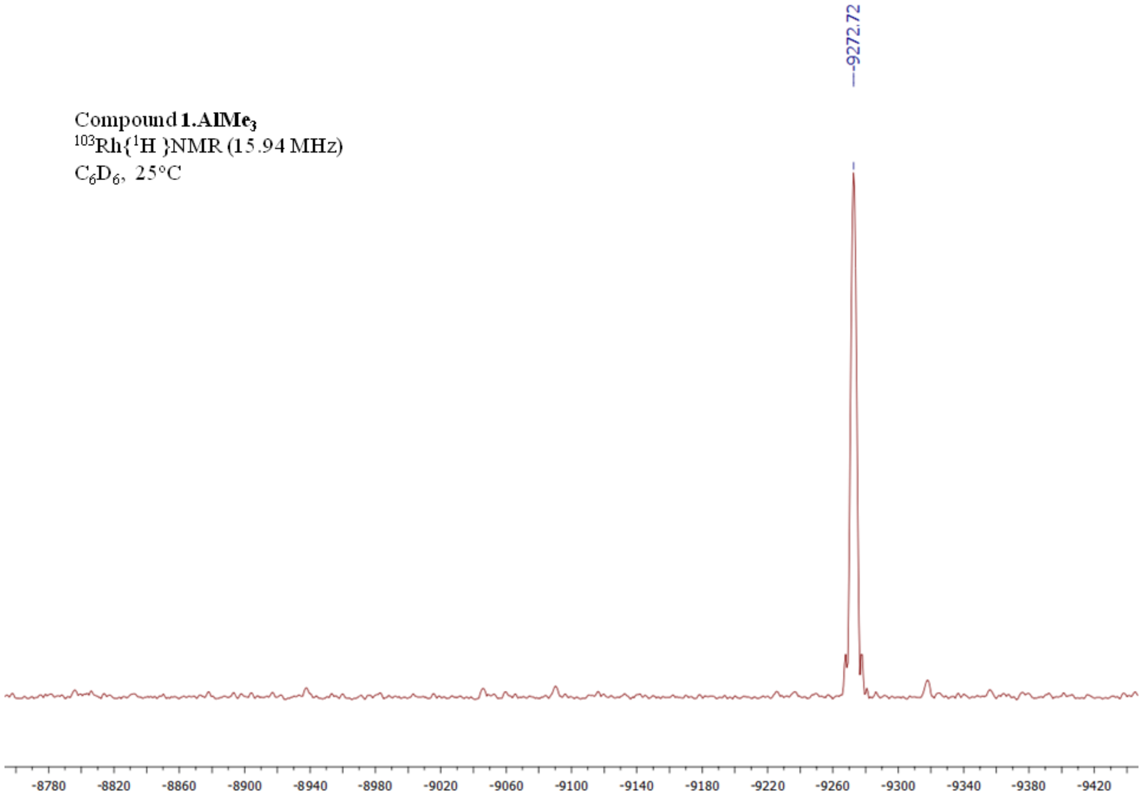
Compound 1.AlMe3
103Rh{1H }NMR (15.94 MHz)
C6D6, 25°C
-9272.72
-8780 -8820 -8860 -8900 -8940 -8980 -9020 -9060 -9100 -9140 -9180 -9220 -9260 -9300 -9340 -9380 -9420

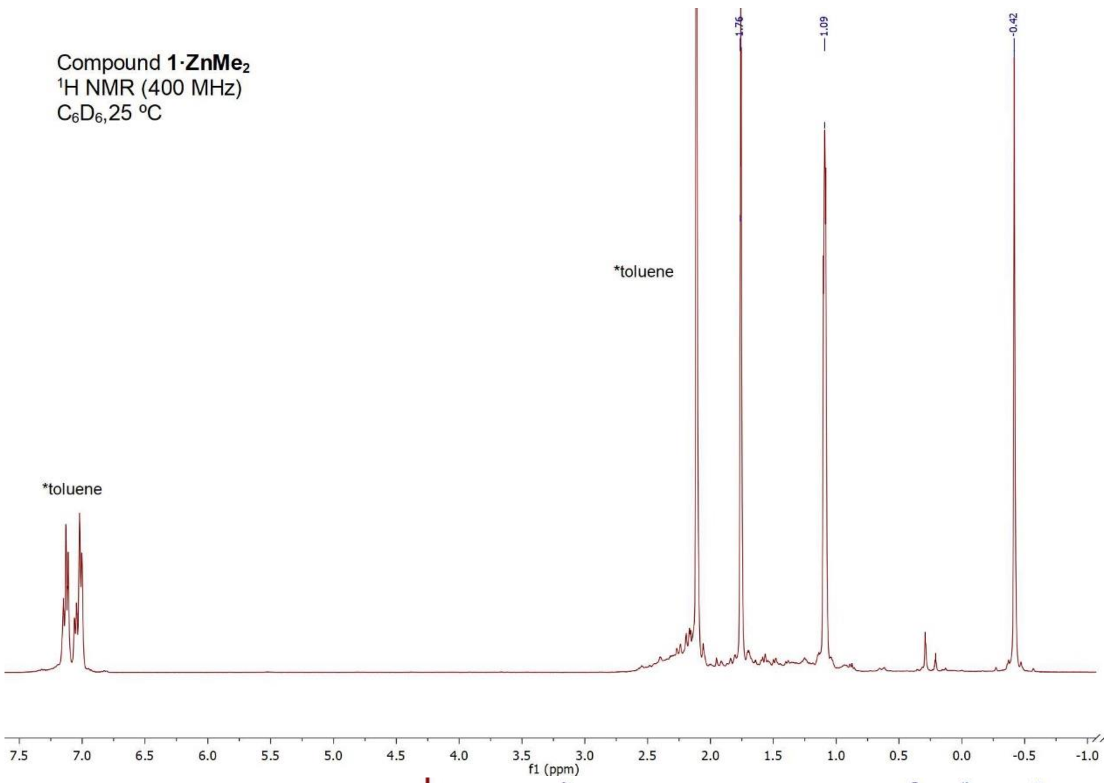
Compound 1·ZnMe2
1H NMR (400 MHz)
C6D6,25 ºC
*toluene
*toluene
1.76
1.09
-0.42
f1 (ppm)

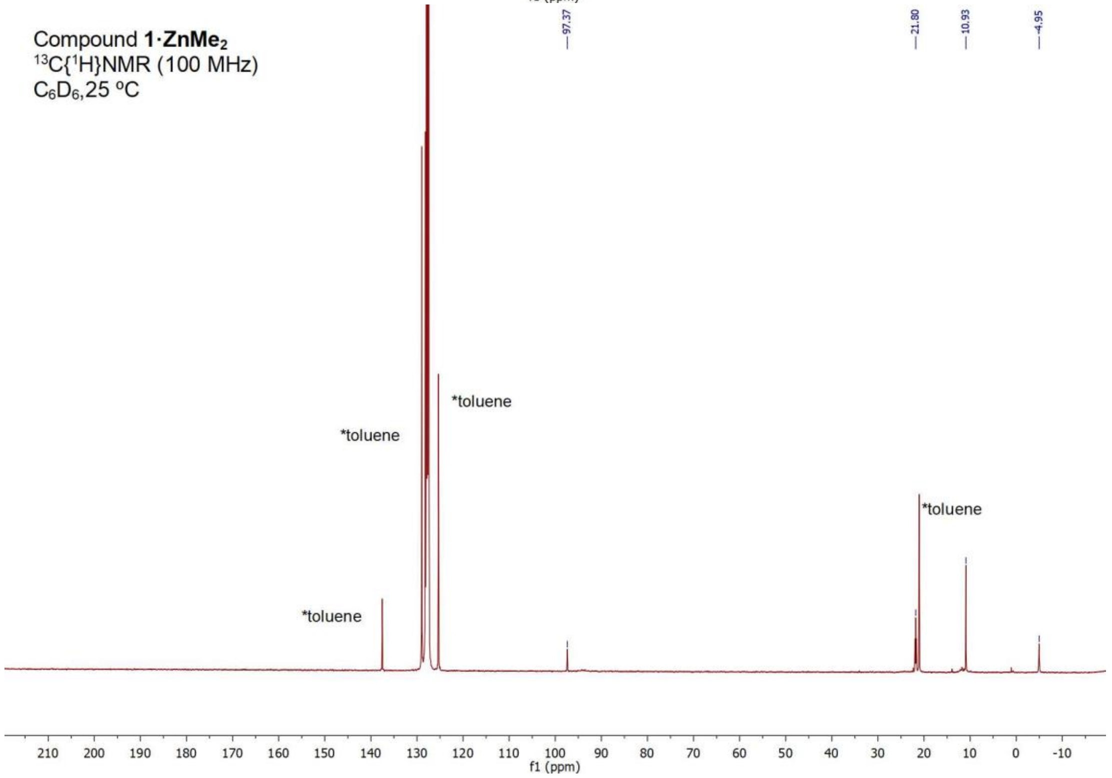
Compound 1·ZnMe2
13C{1H}NMR (100 MHz)
C6D6,25 ºC
97.37
21.80
10.93
-4.95
*toluene
*toluene
*toluene
*toluene
f1 (ppm)

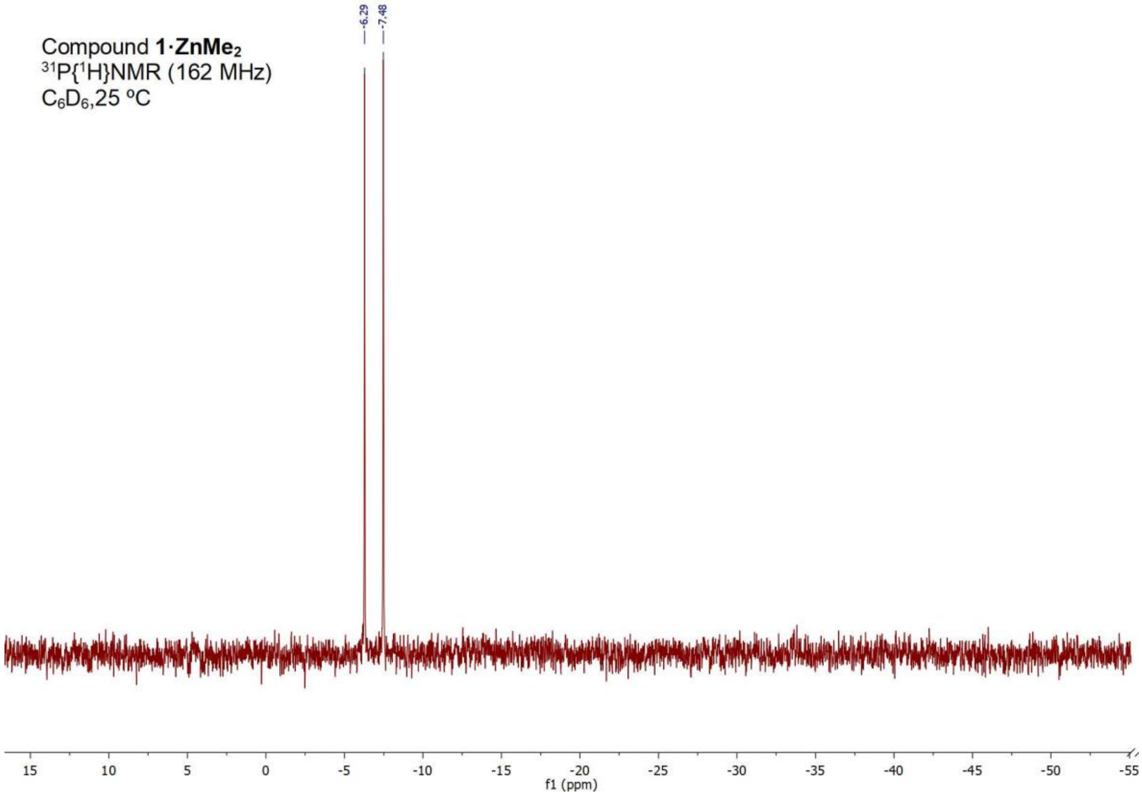
Compound 1·ZnMe2
31P{1H}NMR (162 MHz)
C6D6,25 ºC
-6.29
-7.48
15 10 5 0 -5 -10 -15 -20 -25 -30 -35 -40 -45 -50 -55
f1 (ppm)

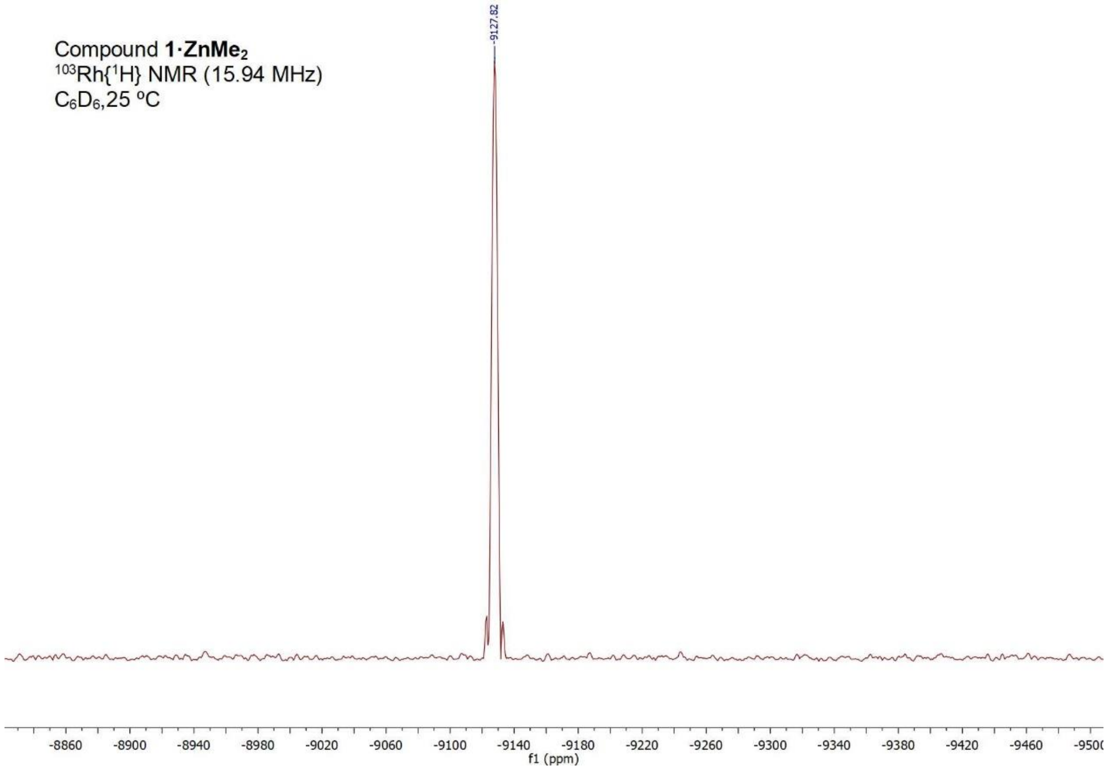
Compound 1·ZnMe2
103Rh{1H} NMR (15.94 MHz)
C6D6,25 ºC
-9127.82
-8860 -8900 -8940 -8980 -9020 -9060 -9100 -9140 -9180 -9220 -9260 -9300 -9340 -9380 -9420 -9460 -9500
f1 (ppm)

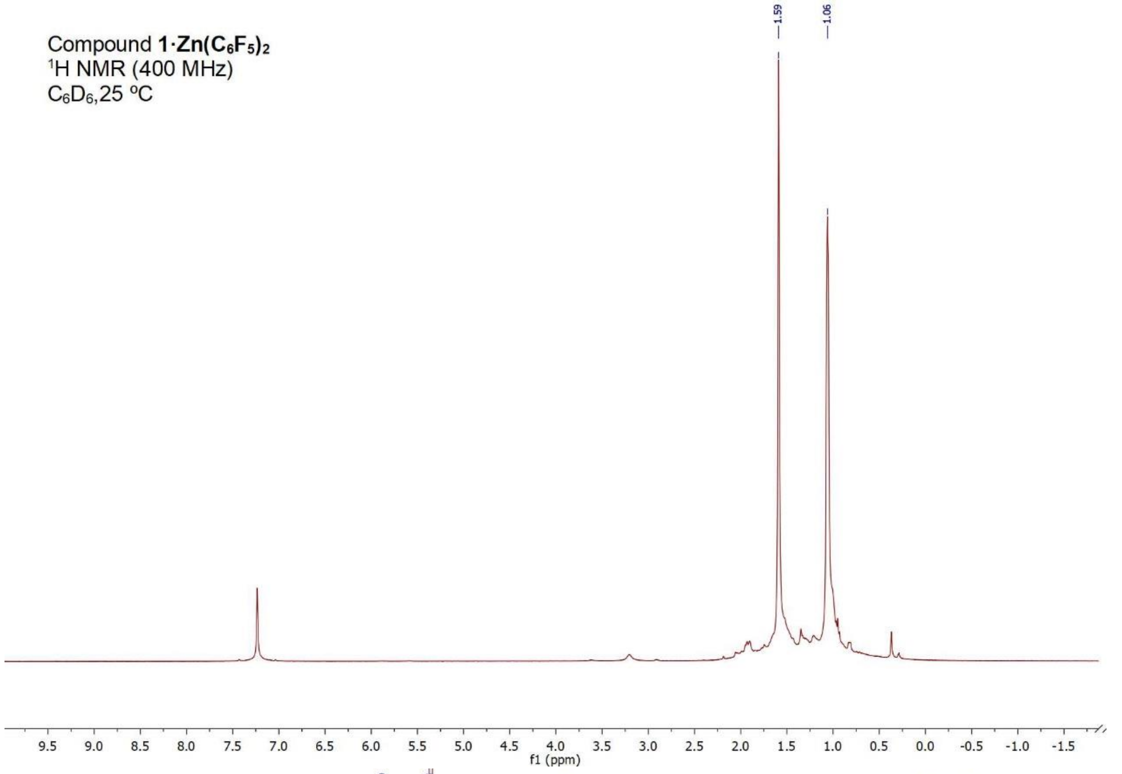
Compound 1·Zn(C6F5)2
1H NMR (400 MHz)
C6D6,25 ºC
1.59
1.06
9.5 9.0 8.5 8.0 7.5 7.0 6.5 6.0 5.5 5.0 4.5 4.0 3.5 3.0 2.5 2.0 1.5 1.0 0.5 0.0 -0.5 -1.0 -1.5
f1 (ppm)

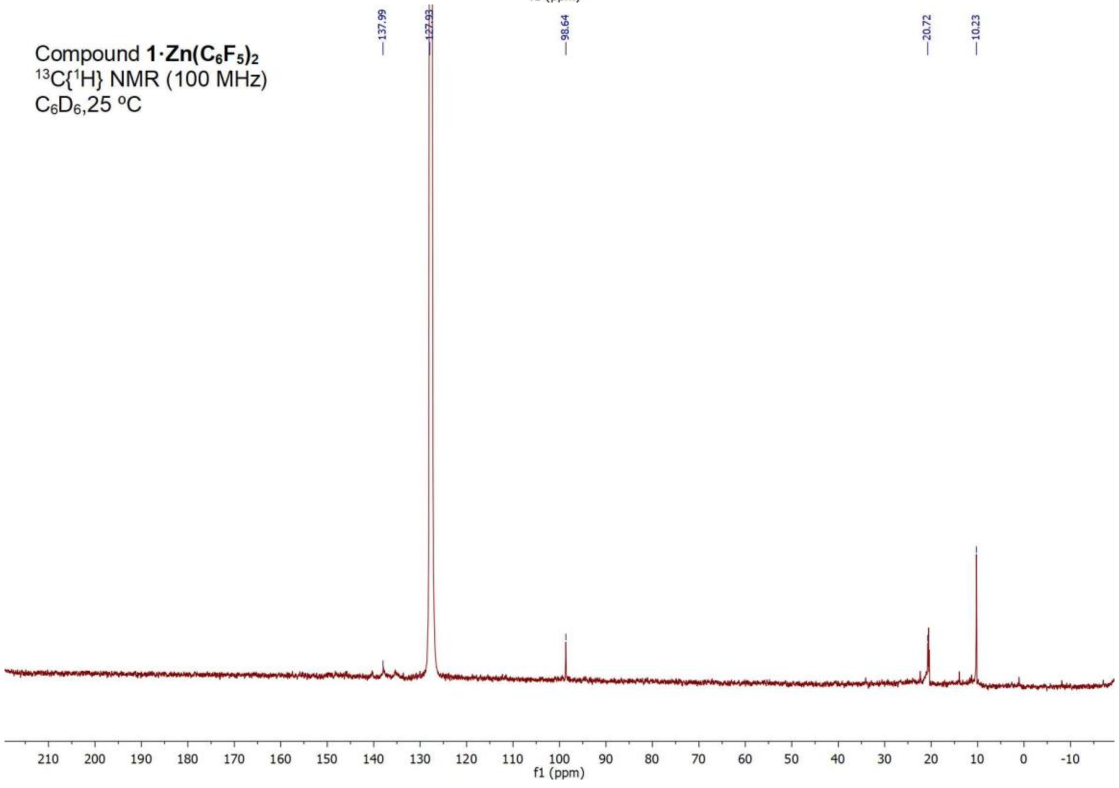
Compound 1·Zn(C6F5)2
13C{1H} NMR (100 MHz)
C6D6,25 ºC
137.99
127.93
98.64
20.72
10.23
210 200 190 180 170 160 150 140 130 120 110 100 90 80 70 60 50 40 30 20 10 0 -10
f1 (ppm)

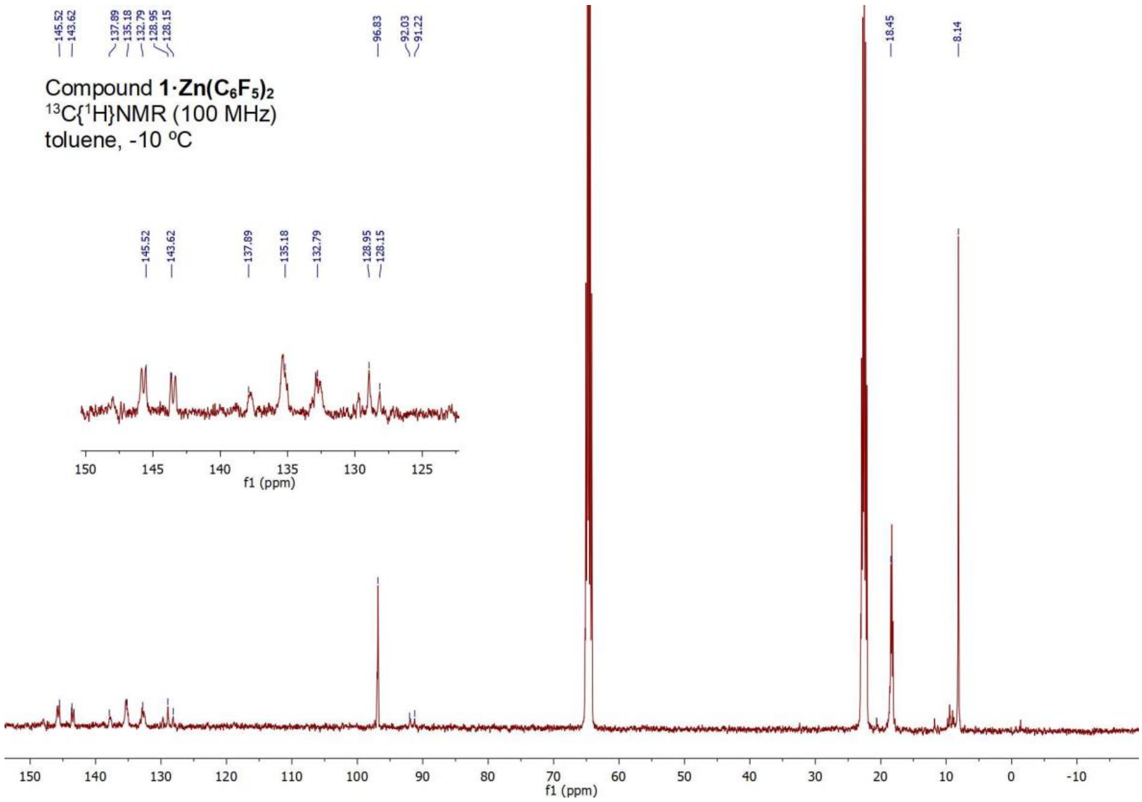

Figure. Carbon NMR sprectrum of RhCp*(PMe)$_3$Zn(C_6F_5)$_2$. With a apliation of the signal corresponding with the carbon of C_6F_5 ligand.

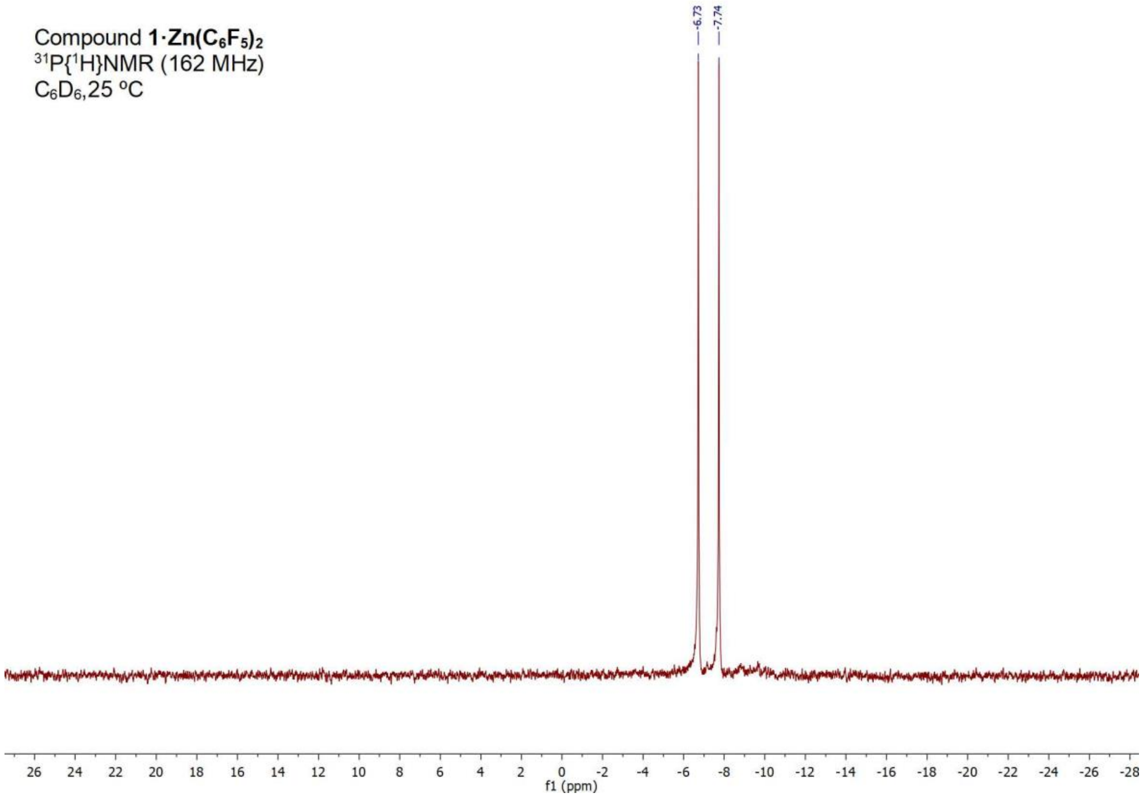

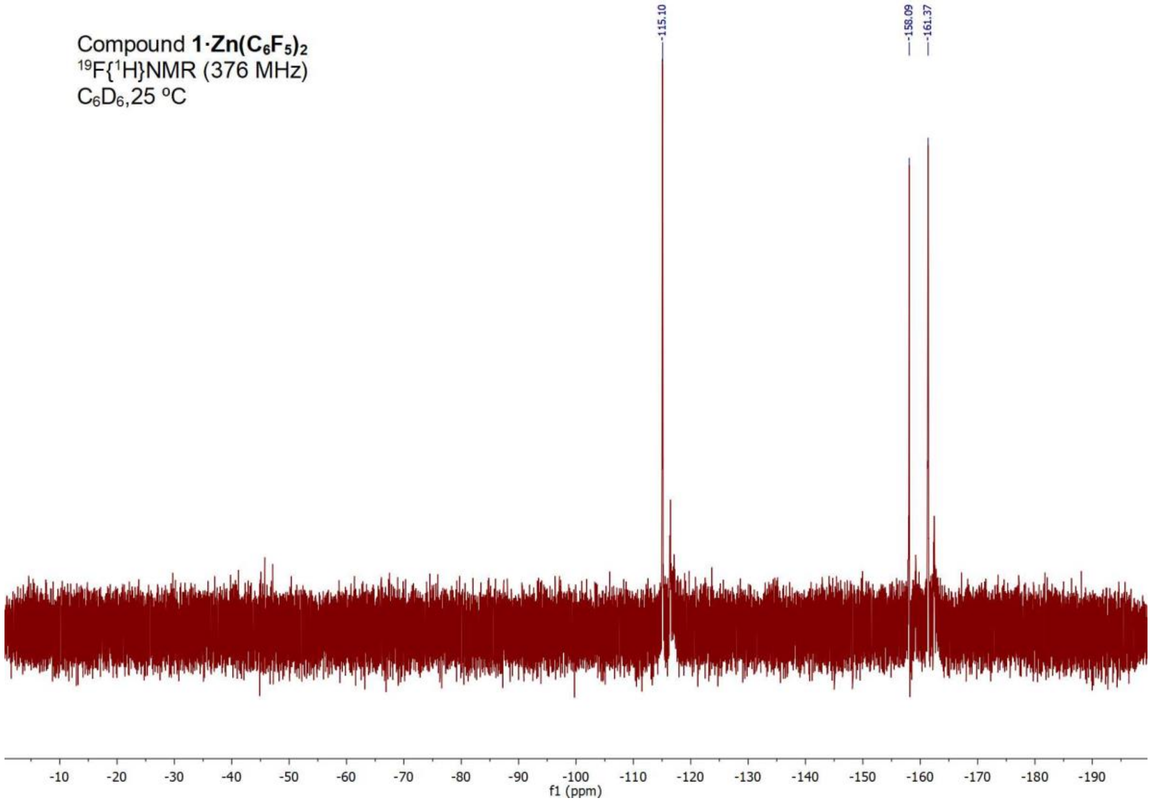

Compound 1·Zn(C6F5)2
19F{1H}NMR (376 MHz)
C6D6, 25 ºC
-115.10
-158.09
-161.37
f1 (ppm)

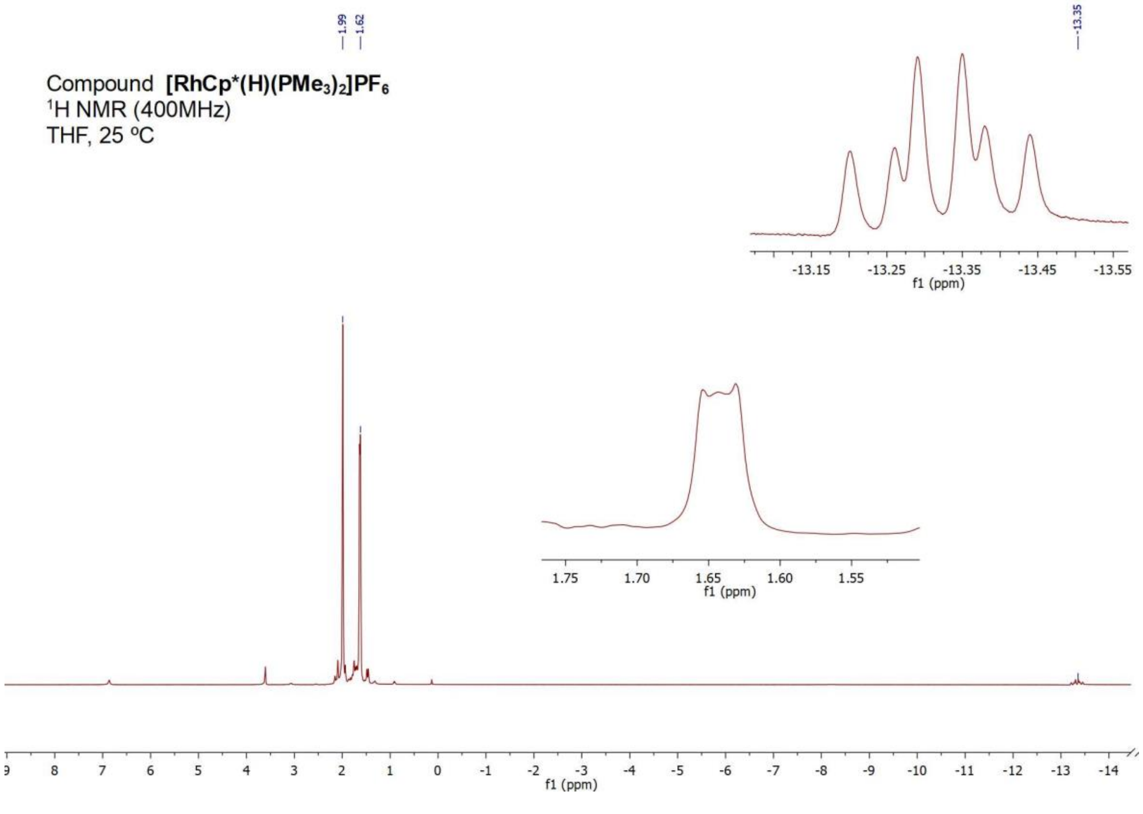

Compound [RhCp*(H)(PMe3)2]PF6
1H NMR (400MHz)
THF, 25 ºC
1.99
1.62
-13.35
f1 (ppm)

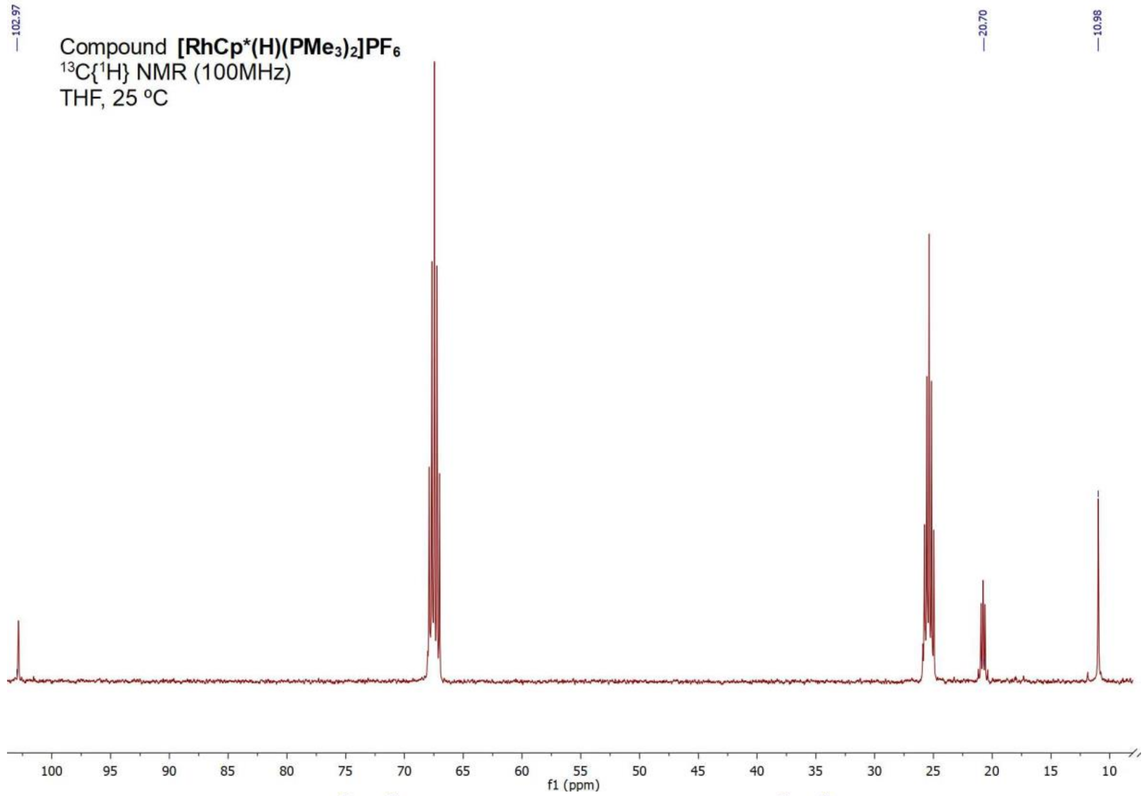
Compound [RhCp*(H)(PMe3)2]PF6
13C{1H} NMR (100MHz)
THF, 25 ºC
102.97
20.70
10.98
f1 (ppm)

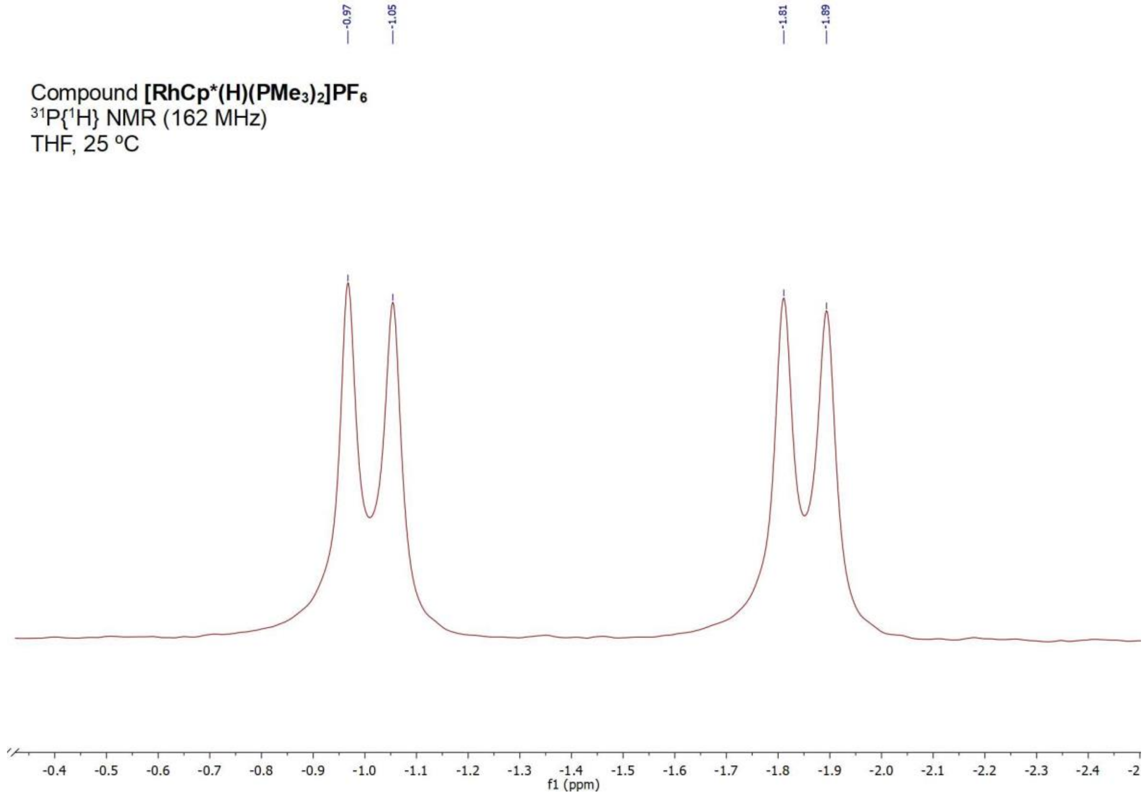
-0.97
-1.05
-1.81
-1.89
Compound [RhCp*(H)(PMe3)2]PF6
31P{1H} NMR (162 MHz)
THF, 25 ºC
f1 (ppm)

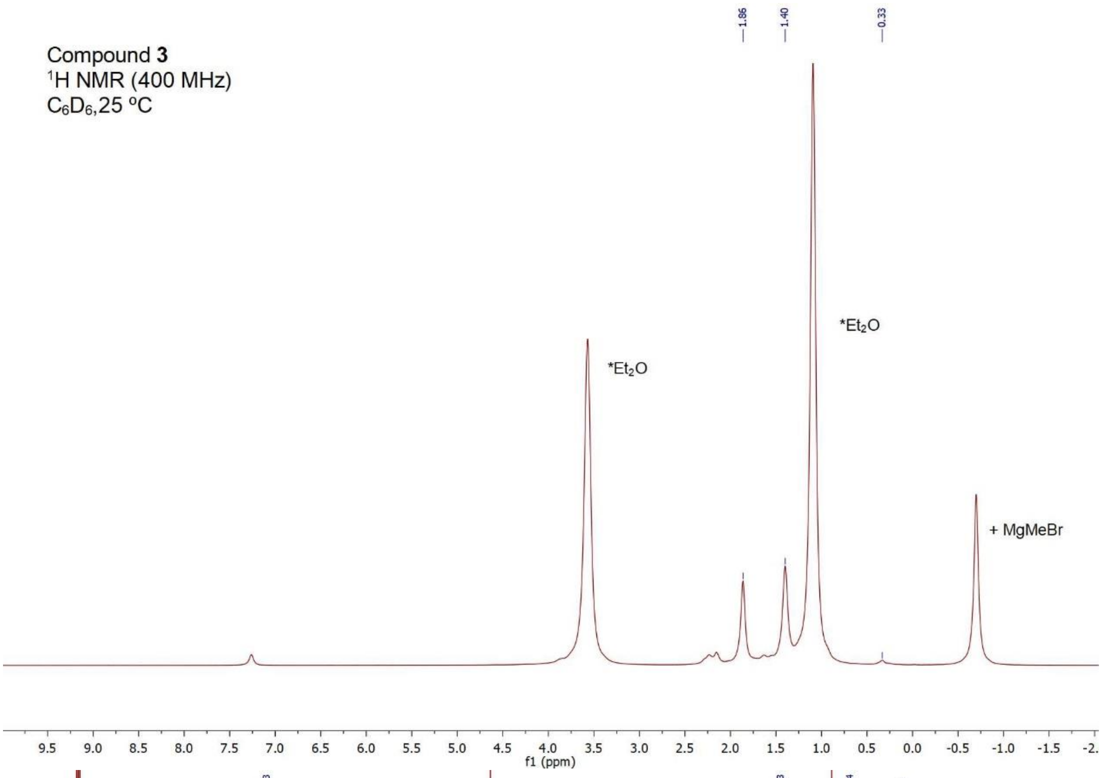
Compound 3
1H NMR (400 MHz)
C6D6,25 ºC
1.86
1.40
0.33
*Et2O
*Et2O
+ MgMeBr
f1 (ppm)

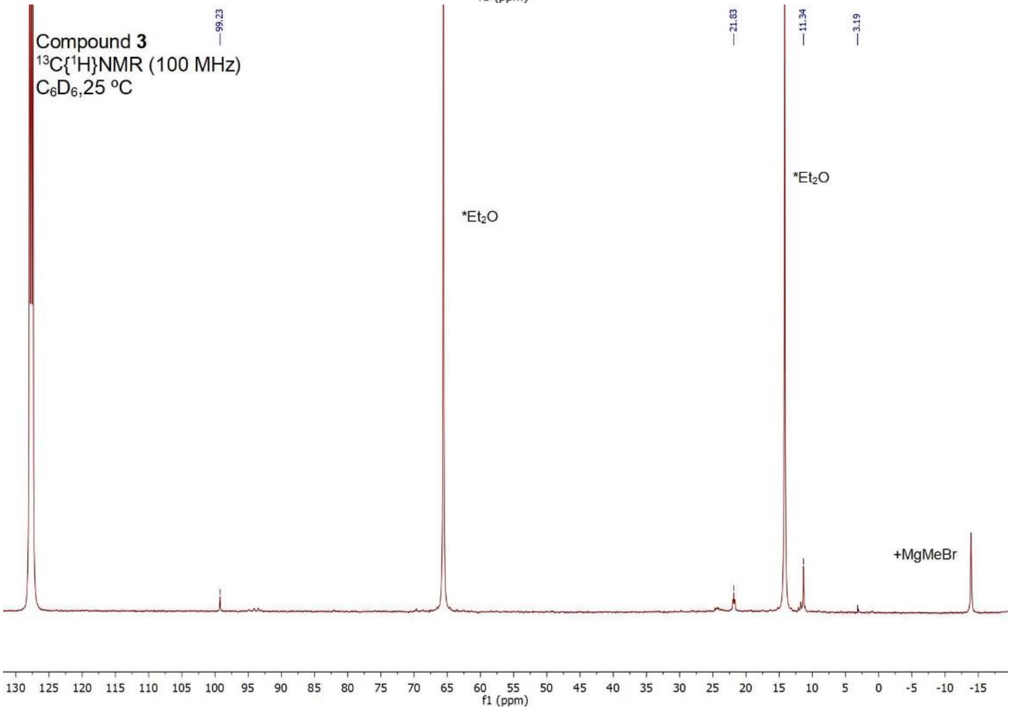
Compound 3
13C{1H}NMR (100 MHz)
C6D6,25 ºC
99.23
21.83
11.34
3.19
*Et2O
*Et2O
+MgMeBr
f1 (ppm)

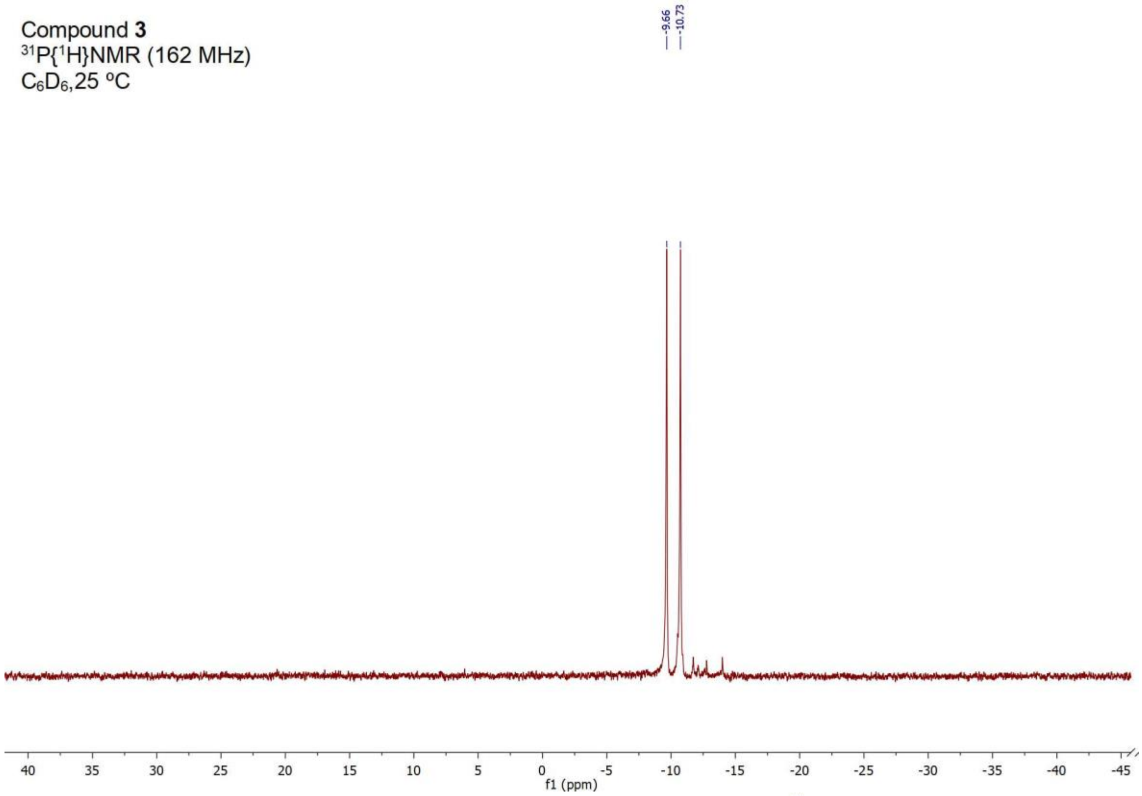
Compound 3
$^{31}P\{^1H\}$NMR (162 MHz)
C_6D_6,25 ºC
-9.66
-10.73
40
35
30
25
20
15
10
5
0
-5
-10
-15
-20
-25
-30
-35
-40
-45
f1 (ppm)

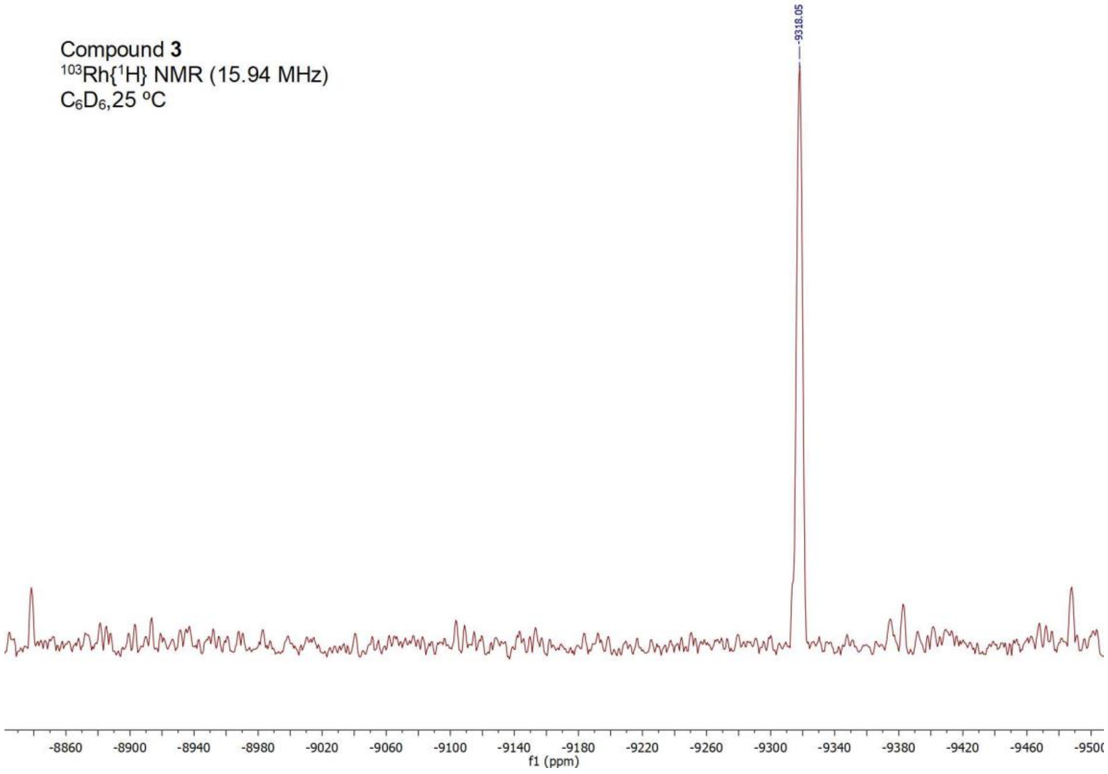
Compound 3
$^{103}Rh\{^1H\}$ NMR (15.94 MHz)
C_6D_6,25 ºC
-9318.05
-8860
-8900
-8940
-8980
-9020
-9060
-9100
-9140
-9180
-9220
-9260
-9300
-9340
-9380
-9420
-9460
f1 (ppm)

4. Additional Computational Results

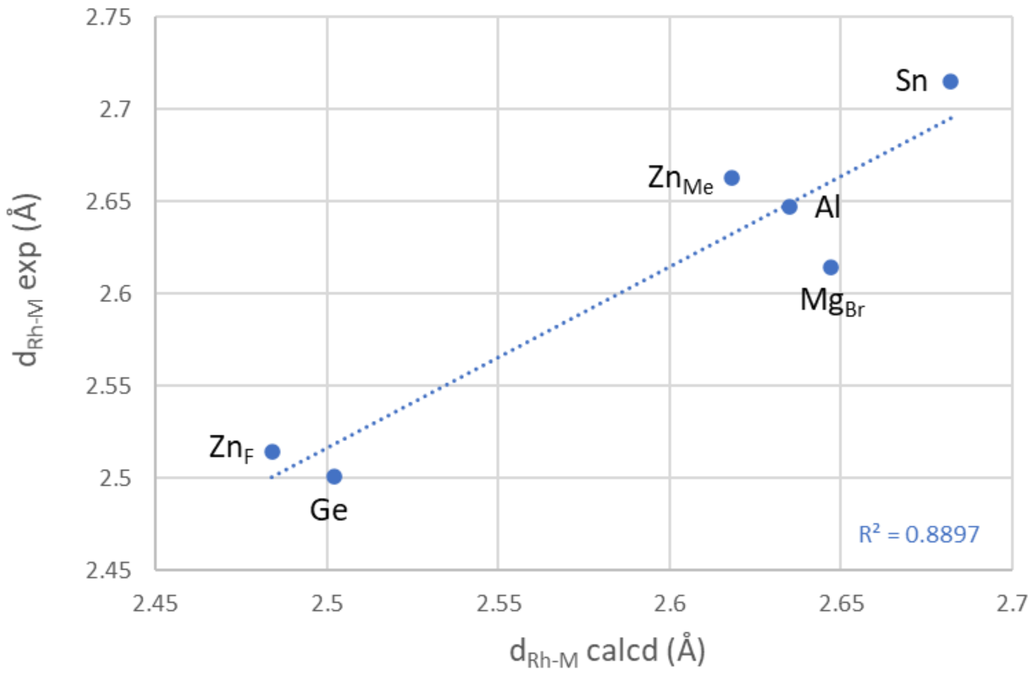

Figure S1. Correlation between the experimental and calculated Rh—M distances.

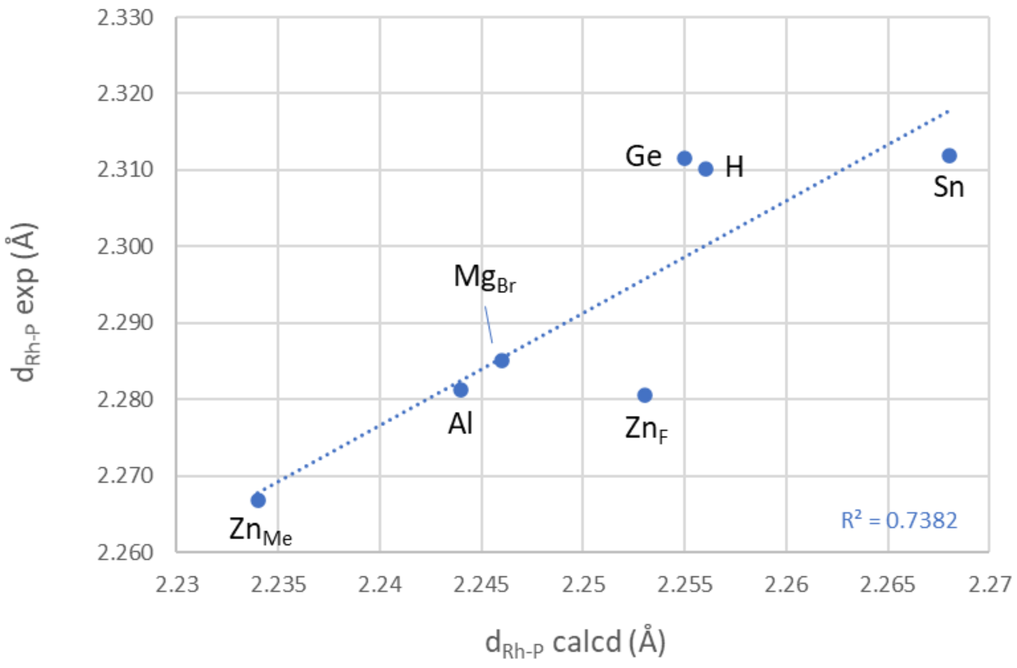

Figure S2. Correlation between the experimental and calculated Rh—P distances.

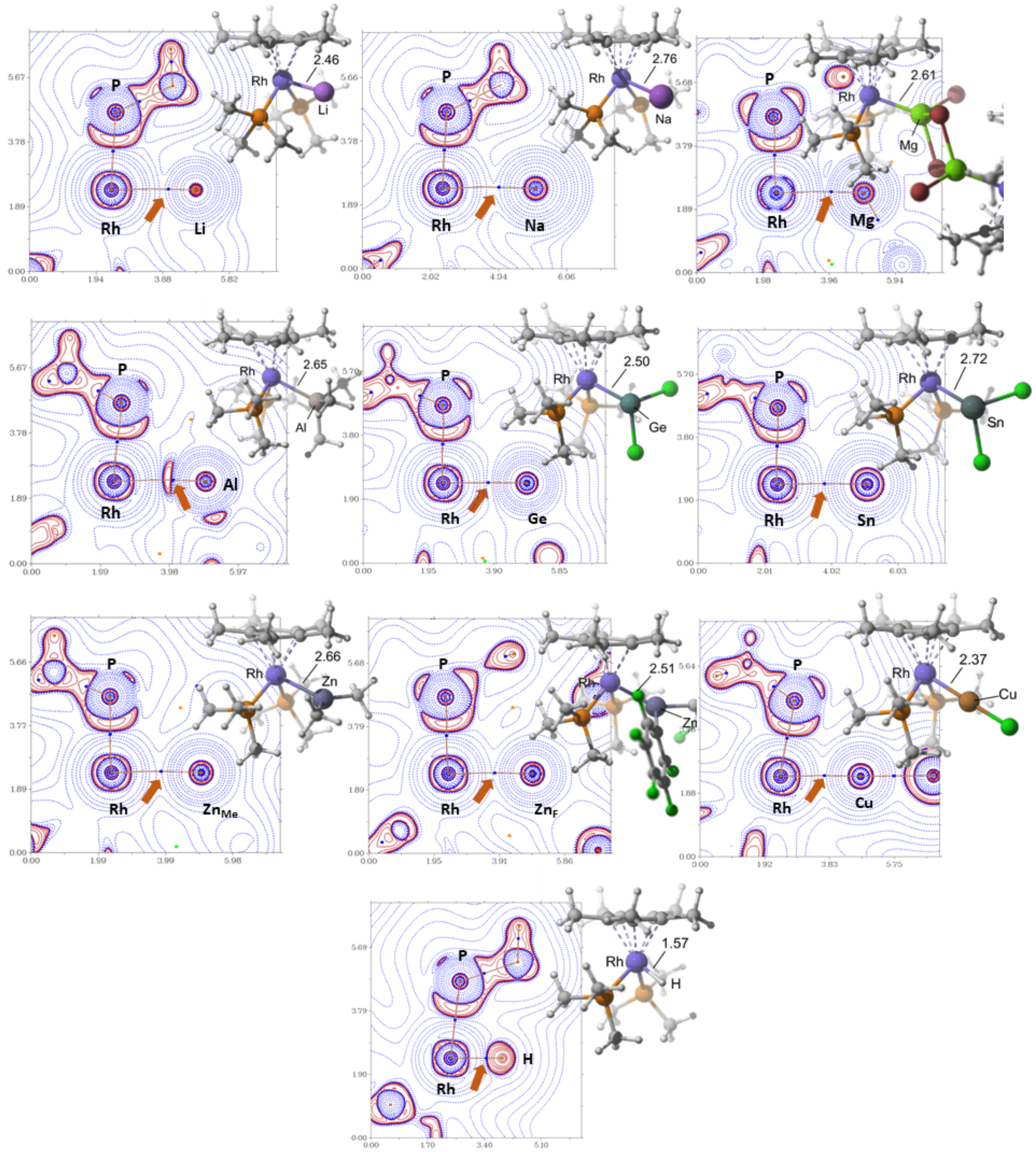

Figure S3. BCPs (blue dots) and bond paths (orange trace) of the electron density of the species considered in this study superimposed on the function $\boldsymbol{L} = - \nabla^2\boldsymbol{\rho_b}$ in one of the M—Rh—P planes. The orange arrows point to the Rh—M BCPs. Dotted blue and solid red contour lines are for positive and negative values of $\boldsymbol{L}$. The optimized geometries of the adducts are also shown. Distances are in Å.

Table S3. QTAIM indicators at Rh(I)—M BCPs. All data are in atomic units unless said otherwise. $\boldsymbol{\rho_b}$ electron density (e·bohr^{-3}); $\mathbf{H_b}$ total energy density (hartree·bohr^{-3}); $\nabla^2\boldsymbol{\rho_b}$ Laplacian of the electron density (e·bohr^{-5}); $|\mathbf{V_b}|/\mathbf{G_b}$ ratio between the absolute electronic potential energy and kinetic energy densities; λ_i eigenvalues of the Hessian matrix; $\boldsymbol{\varepsilon_b}$ (ellipticity) ratio between the largest and smallest negative eigenvalues of the Hessian - 1.

	bond	$\rho(r)$	$\rho(r)$ (e·Å^{-3})	G_b	V_b	H_b	$\|V_b\|/G_b$	$G_b/\rho(r_b)$	$\nabla^2\rho_b$	$\nabla^2\rho_b$ (e·Å^{-5})	λ_3	λ_2	λ_1	ε_b
**RhCp (1)**	Rh-P (average)	0.1066	0.7192	0.0870	-0.1329	-0.0459	1.527	0.8166	0.1647	3.9694	0.3283	-0.0832	-0.0804	0.035
**H (hydride)**	Rh-H	0.1505	1.0153	0.0974	-0.1924	-0.0950	1.975	0.6475	0.0098	0.2366	0.5288	-0.2602	-0.2587	0.006
	Rh-P (average)	0.1014	0.6842	0.0703	-0.1122	-0.0420	1.597	0.6930	0.1132	2.7281	0.2825	-0.0854	-0.0839	0.019
**Li^+**	Rh-Li	0.0236	0.1590	0.0208	-0.0220	-0.0012	1.059	0.8832	0.0783	1.8881	0.1244	-0.0238	-0.0222	0.071
	Rh-P (average)	0.1076	0.7262	0.0846	-0.1314	-0.0468	1.553	0.7861	0.1514	3.6482	0.3212	-0.0871	-0.0826	0.054
**Na^+**	Rh-Na	0.0201	0.1359	0.0172	-0.0169	0.0003	0.981	0.8530	0.0700	1.6867	0.0994	-0.0152	-0.0143	0.066
	Rh-P (average)	0.1047	0.7063	0.0830	-0.1273	-0.0443	1.534	0.7928	0.1546	3.7250	0.3173	-0.0833	-0.0794	0.048
**$MgBr_2$**	Rh-Mg (average)	0.0316	0.2136	0.0269	-0.0301	-0.0032	1.120	0.8489	0.0945	2.2780	0.1422	-0.0241	-0.0236	0.023
**$AlMe_3$**	Rh-Al	0.0390	0.2631	0.0206	-0.0347	-0.0141	1.683	0.5292	0.0261	0.6295	0.0855	-0.0304	-0.0290	0.050
	Rh-P (average)	0.1061	0.7157	0.0799	-0.1257	-0.0458	1.572	0.7537	0.1368	3.2956	0.3114	-0.0898	-0.0849	0.058
**$GeCl_2$**	Rh-Ge	0.0721	0.4866	0.0324	-0.0593	-0.0269	1.830	0.4491	0.0220	0.5296	0.1406	-0.0600	-0.0586	0.024
	Rh-P (average)	0.1024	0.6913	0.0731	-0.1160	-0.0429	1.588	0.7133	0.1205	2.9045	0.2911	-0.0876	-0.0830	0.055
**$SnCl_2$**	Rh-Sn	0.0571	0.3853	0.0261	-0.0430	-0.0169	1.649	0.4563	0.0366	0.8810	0.1158	-0.0399	-0.0393	0.013
	Rh-P (average)	0.1022	0.6899	0.0741	-0.1168	-0.0427	1.576	0.7252	0.1257	3.0301	0.2943	-0.0865	-0.0820	0.055
**$Zn(Me)_2$**	Rh-Zn	0.0389	0.2626	0.0261	-0.0339	-0.0078	1.300	0.6705	0.0731	1.7612	0.1307	-0.0289	-0.0287	0.008
**$Zn(C_6F_5)_2$**	Rh-Zn	0.0553	0.3732	0.0379	-0.0545	-0.0166	1.437	0.6860	0.0691	1.6644	0.1756	-0.0460	-0.0442	0.042
**CuCl**	Rh-Cu	0.0709	0.4785	0.0625	-0.0874	-0.0249	1.399	0.8817	0.1504	3.6244	0.2813	-0.0665	-0.0644	0.032

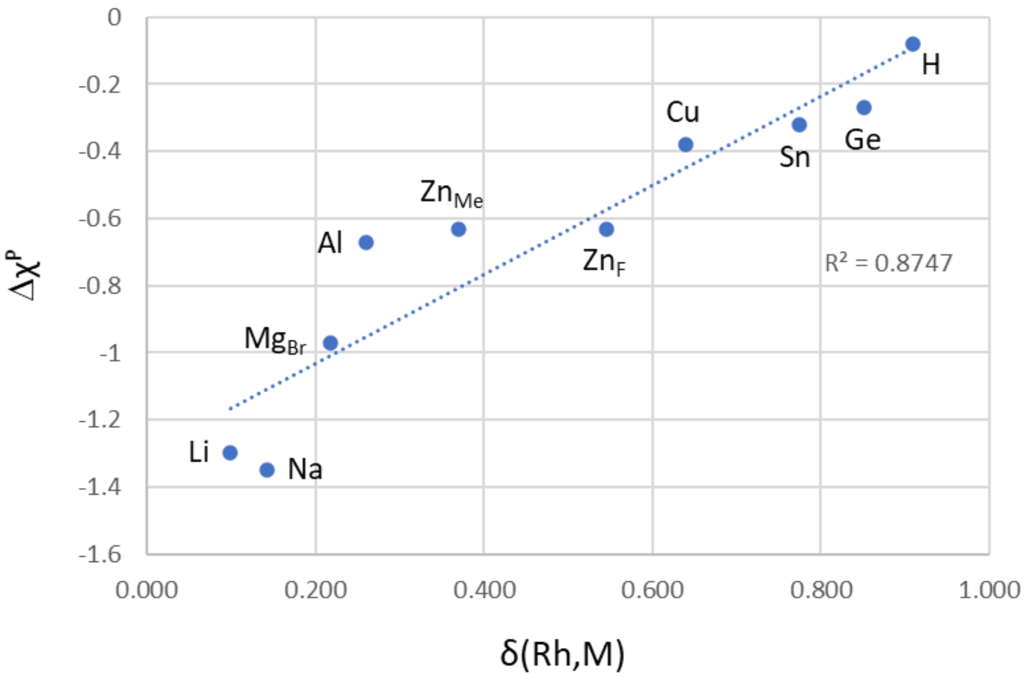

Figure S4. Correlation of the Pauling electronegativity difference ($\Delta\chi^P = \chi^P(M\ or\ H) - \chi^P(Rh)$) with the delocalization indices between Rh and M (H) atoms of the species considered in this study.

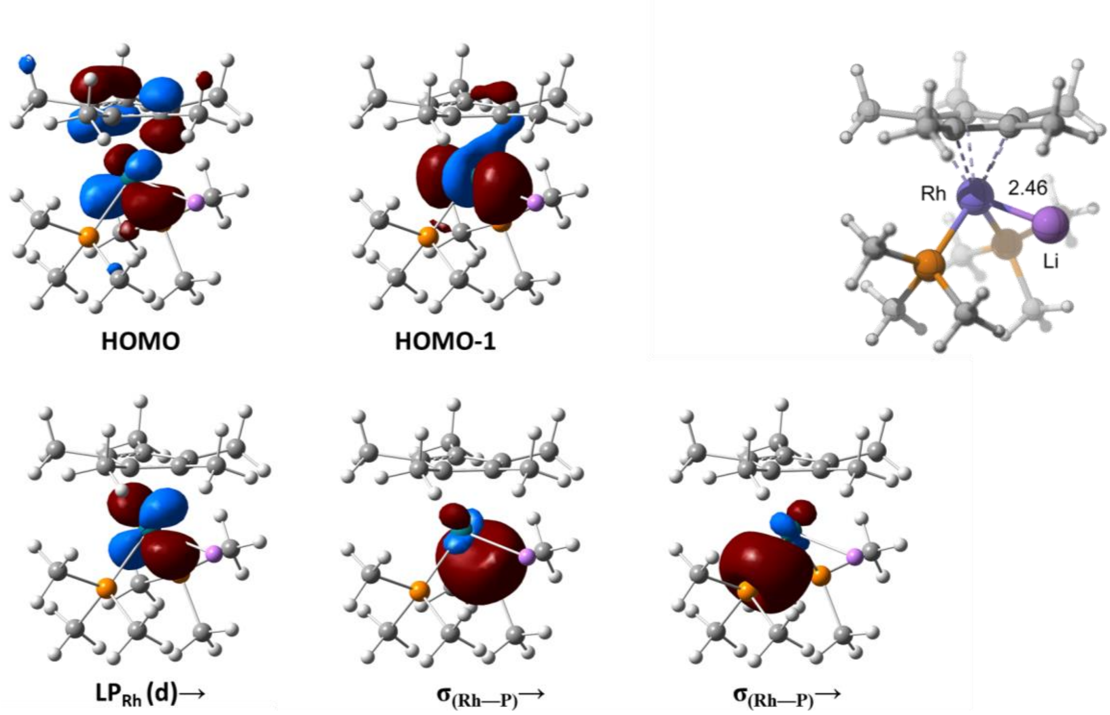

Figure S5. Relevant MOs and (isosurface value 0.05 a.u.) NBOs (isosurface value 0.06 a.u.) for **1·Li**.

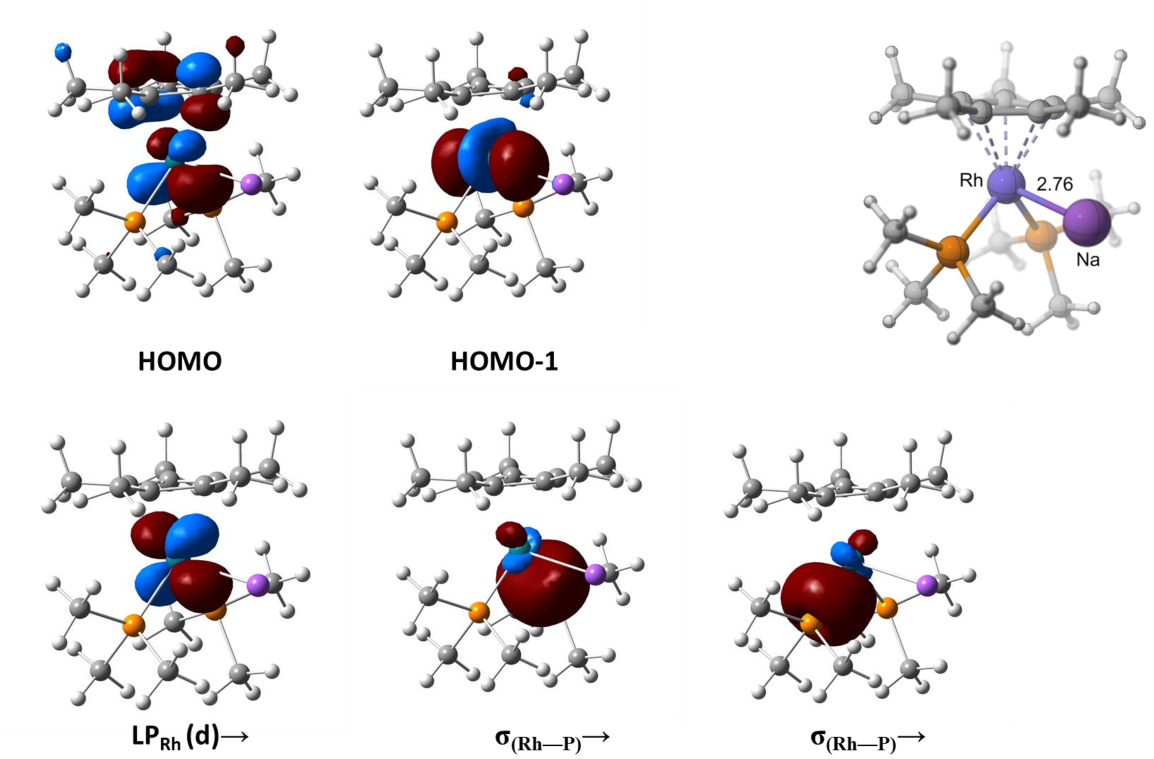

Figure S6. Relevant MOs and (isosurface value 0.05 a.u.) NBOs (isosurface value 0.06 a.u.) for **1·Na**.

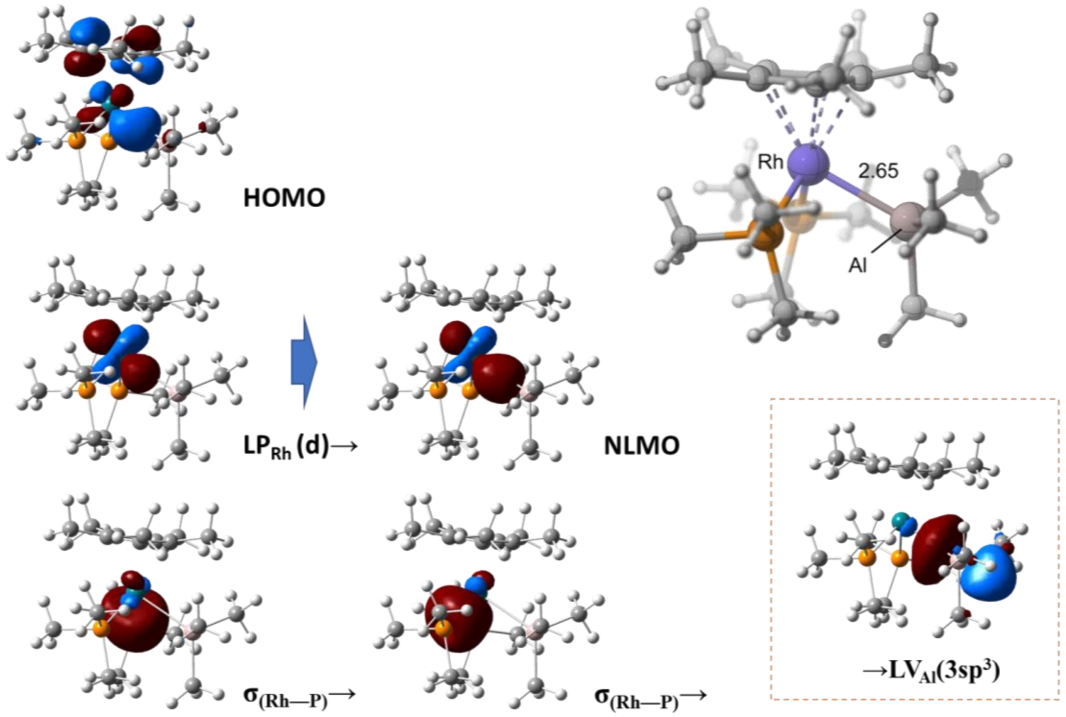

Figure S7. HOMO (isosurface value 0.05 a.u.) and relevant NBOs and associated NLMO (isosurface value 0.06 a.u.) for **1·AlMe$_3$**.

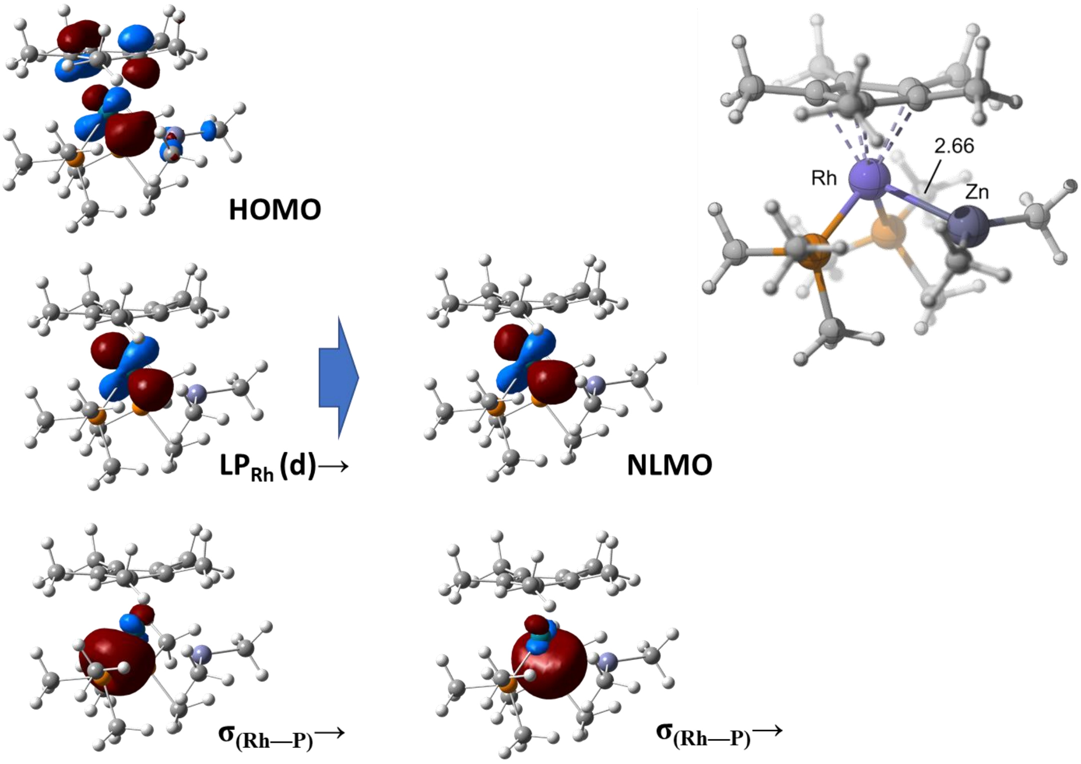

Figure S8. HOMO (isosurface value 0.05 a.u.) and relevant NBOs and associated NLMO (isosurface value 0.06 a.u.) for **1·ZnMe$_2$**.

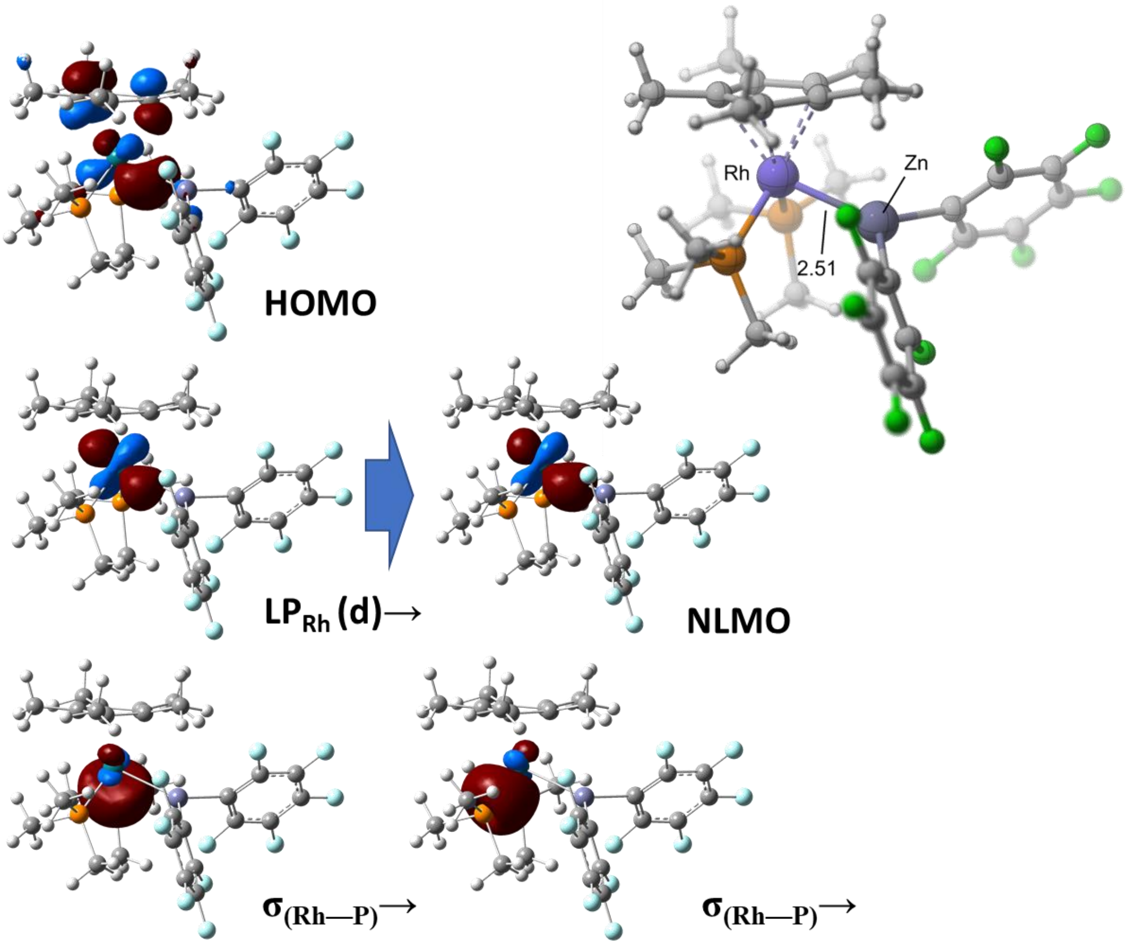

Figure S9. HOMO (isosurface value 0.05 a.u.) and relevant NBOs and associated NLMO (isosurface value 0.06 a.u.) for **1·Zn(C$_6$F$_5$)$_2$**.

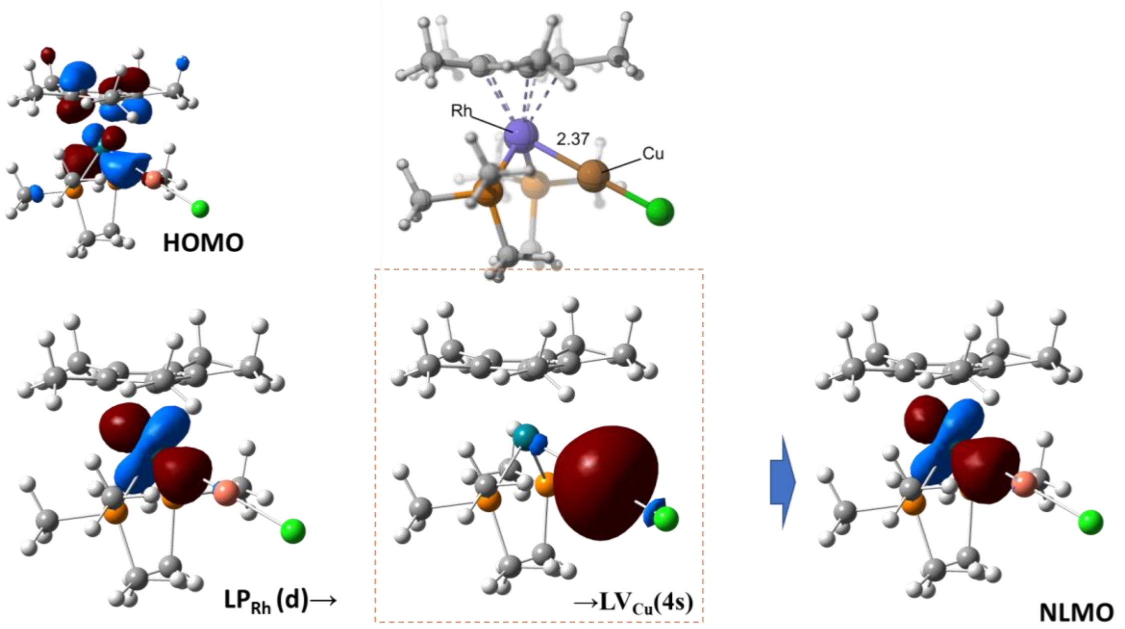

Figure S10. HOMO (isosurface value 0.05 a.u.) and relevant NBOs and associated NLMO (isosurface value 0.06 a.u.) for **1·CuCl**.

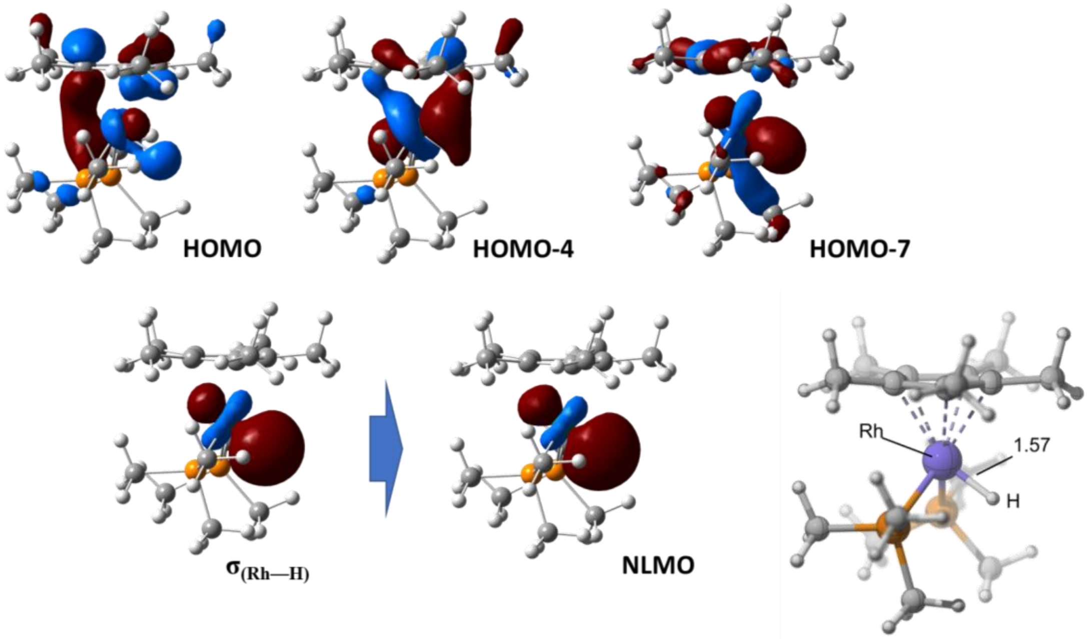

Figure S11. Relevant MOs (isosurface value 0.05 a.u.) and NBO and associated NLMO (isosurface value 0.06 a.u.) for the Rh hydride **2**.

5. References

[1] H. Werner, B. Klingert, B. *Chem. Ber*. **1983**, *116*, 1450- 1462.
[2] J. W. Kang, K. Mosley, P. M. Maitlis, *Chem. Commun.* **1968**, *21*, 1304–1305.
[3] G. M. Sheldrick, *Acta Cryst*. **2008,** *A64*, 112–122.
